# Climate‐Induced Genomic Selection: Genetic Structure Variation of *Cornus kousa* subsp. *chinensis* and Its Adaptive Reaction to Future Climate Change

**DOI:** 10.1111/eva.70281

**Published:** 2026-06-18

**Authors:** Junru Li, Qian Liu, Xi Gong, Luzhang Ruan, Bicai Guan

**Affiliations:** ^1^ College of Life Sciences Nanchang University Nanchang China

**Keywords:** adaptive evolution, annual mean temperature, climate change, *Cornus kousa* subsp. *chinensis*, genetic structure

## Abstract

Global climate change exerts a far‐reaching influence on the geographical distribution, phenological patterns, and genetic diversity of plants, presenting a grave challenge to the survival and evolution of species. Owing to its robust ecological adaptability, restricted dispersal capacity, and abundant intraspecific variation, *Cornus kousa* subsp. *chinensis* has emerged as an ideal subject for investigating the responses of woody plants to climate change. Consequently, this study employed the landscape genomics approach to evaluate the survival risks faced by *C. kousa* under the present and future (2060–2080) climate scenarios (RCP45 and RCP85). A total of 348 leaf samples were gathered from 25 populations of *C. kousa* spanning diverse regions of China. The research revealed that, under the prevailing climate conditions, the genetic diversity within the populations is remarkably abundant. Nevertheless, the POPs model forecasts that the genetic structure will undergo substantial alterations in future climate scenarios. Notably, 93.6% of the populations will be grouped into a solitary common cluster, and the genetic variation will be conspicuously homogenized. Through BayeScan and Latent Factor Mixed Model (LFMM) analyses, 67 Single Nucleotide Polymorphism (SNP) loci significantly associated with climate factors were identified. Among these loci, 90.3% are closely correlated with the annual mean temperature (Bio 1), suggesting that temperature serves as the primary driving force for adaptive selection at the genomic level. In summary, our findings not only offer valuable perspectives for the conservation of the genetic resources of the *C. kousa* population but also provide significant reference value for the formulation of subtropical forest conservation strategies.

## 1 Introduction

Anthropogenic climate change stands as one of the most prevalent perils to global biodiversity (Wu et al. 2022; Wudu et al. 2023). Propelled by the precipitous surge in the concentration of greenhouse gases within the atmosphere, the Earth's climate system is experiencing unparalleled transformations. These alterations are not merely manifested in the elevation of the global mean temperature; they also encompass the modification of precipitation regimes, the escalation in the frequency and severity of extreme weather phenomena, and the extensive degradation of ecosystems (Asif 2023; Verma 2022). For the vast majority of plant species lacking the ability of autonomous locomotion, climate, and other environmental vicissitudes present a grave challenge. The linchpin of their survival lies in whether they can endure the novel climatic conditions within their current habitats, pursue the dynamically shifting climatic niches via migration, or confront the new environmental pressures through genetic adaptation (Anderson and Song 2020). Although numerous studies have employed species distribution models (SDMs) to forecast historical alterations in the geographical distribution of species, there is a growing realization that comprehending the concomitant changes in intraspecific genetic diversity and genetic structure is not only of great significance but also pivotal for evaluating the long‐term viability and evolutionary potential of species (Lecocq et al. 2019; Hu et al. 2021). Genetic diversity serves as the fundamental cornerstone of evolution. It furnishes the essential raw materials for natural selection, empowering populations to adapt to the ever‐changing environment. Consequently, climate change is not merely an ecological occurrence; rather, it is a potent evolutionary impetus. At an accelerating tempo, it is reshaping the genetic blueprint of biodiversity (Gaitan‐Espitia and Hobday 2020).

Climate change exerts a profound genetic influence on plant populations via a multitude of interrelated mechanisms. Initially, swift environmental transformations may give rise to intense directional selection, conferring an advantage upon alleles that can enhance fitness under novel environmental circumstances. Provided that there exists sufficient pre‐existing genetic variation within the population, this process can facilitate rapid adaptive evolution. Nevertheless, if the pace of climate change outstrips the capacity for adaptive evolution, the risk of extinction will escalate (Garcia‐Costoya et al. 2023; Meester et al. 2018; Anderson and Song 2020). Second, habitat fragmentation and population decline spurred by climate change may precipitate genetic erosion. As the population size dwindles and isolation intensifies, genetic drift and inbreeding will assume increasingly prominent roles, culminating in the loss of rare alleles and a diminution of heterozygosity. The depletion of genetic diversity will undermine the population's adaptive potential, thus initiating a pernicious cycle known as the “extinction vortex” (Giraldo 2020; Nordstrom et al. 2023). Thirdly, alterations in climatic conditions may bring about shifts in gene‐flow patterns (Bontrager and Angert 2018). For instance, phenological modifications (such as variations in flowering time) may disrupt the synchronization among previously interconnected populations, thereby curbing the gene flow facilitated by pollen. Conversely, the expansion or migration of the distribution range triggered by climate change may enable previously isolated populations to come into contact. This, in turn, can foster gene flow and potentially introduce preadapted alleles into the endangered populations (Rivest et al. 2021; Zu et al. 2023). The combined impacts of selection, genetic drift, and gene flow dictate the future genetic architecture of species, subsequently influencing their capacity to endure in the context of ongoing global change (Razgour et al. 2019). Hence, it is of paramount significance to comprehend these genetic and evolutionary processes at the mechanistic level, transcending mere predictions of distributional alterations. This understanding is crucial for the formulation of effective conservation priority strategies and management measures.

Landscape genomics has evolved into a revolutionary field that amalgamates the theories and methodologies of population genetics, landscape ecology, and biogeography to directly address the aforementioned intricate issues (Rico 2019). By integrating high‐throughput genomic data with precise spatial and environmental data, this discipline empowers researchers to discern the correlation between genomic variation and environmental gradients, simultaneously eliminating the interference induced by neutral population structure and geographical factors (Cruzan and Hendrickson 2020; Cushman et al. 2018). Within this research framework, the pivotal methods, for instance, the Genome‐Environment Association (GEA) analysis, which employs tools like BayeScan and LFMM, can identify “outlier loci.” These loci exhibit an unusually strong association with specific climate variables. They are likely subject to the influence of divergent selection and serve as candidate loci for local adaptation (Caye et al. 2019). Moreover, landscape genomics is capable of forecasting future genetic scenarios. It achieves this by simulating and predicting the alterations in the distribution of adaptive genetic variation within climate‐change scenarios and by evaluating the risk of the loss of adaptive alleles or the disintegration of coadaptive gene complexes. This potent predictive capacity renders landscape genomics an essential instrument for foreseeing the evolutionary path of species amidst climate change and for pinpointing populations that can serve as the crucial adaptive diversity reservoir for species resilience (Razgour et al. 2019; Wang et al. 2023).

*Cornus kousa* subsp. *chinensis* represents an exemplary model system for investigating the influence of climate change through the application of landscape genomics. As a deciduous shrub or diminutive tree that is indigenous to the subtropical and warm‐temperate forests of central and eastern China, it inhabits a diverse array of habitats characterized by substantial environmental heterogeneity (Guan et al. 2021). This extensive spectrum of ecological adaptability suggests that the species has an evolutionary chronicle of acclimatizing to a variety of local environments, a trait that might be mirrored in its genomic architecture. More crucially, its life‐history attributes render it especially susceptible to the genetic repercussions of climate change. Prior investigations in phylogeography and population genetics, which employed chloroplast DNA and simple sequence repeat (SSR) markers, revealed that there was notable genetic divergence among the populations of this species, and gene flow was relatively circumscribed (Chen 2018). This aligns with the primary seed‐dispersal mechanism. The restricted dispersal ability, coupled with the fragmentation of natural habitats induced by human endeavors, renders it arduous for the species to keep pace with the shifting climate niche via rapid migration (Yuan et al. 2019). Remarkably, the natural range of *C. kousa* reaches into the higher‐latitude warm‐temperate zones of northern China (for instance, the southern parts of Shaanxi, Shanxi, and Gansu provinces, extending up to roughly 35° N) (Xiang and Boufford 2005). The populations in these regions are subjected to harsher winters, more pronounced seasonal temperature oscillations, and distinctive precipitation patterns when compared to those in the central subtropical belt. These environmental gradients spanning latitudes are apt to exert divergent selective forces. Consequently, this species offers an outstanding landscape‐scale framework to explore genomic reactions to climatic heterogeneity along latitudinal temperature gradients. Significantly, its physiological seed dormancy (which necessitates cold stratification) and the ability to propagate clonally through root suckers may exert a profound impact on how genetic diversity is preserved and structured under the influence of climate change (Fu et al. 2013). Consequently, *C. kousa* might highly depend on the existing genetic variation and local adaptation as the principal strategies to deal with environmental alterations. This also positions its genetic well‐being and evolutionary potential as the core concerns influencing its future survival. Nevertheless, there has yet to be a comprehensive evaluation of its genome‐wide variation pattern and its correlation with climate factors, which severely constrains our comprehension of its adaptability.

Consequently, this study employs landscape genomics to evaluate the influence of present and future climate change on the genetic architecture and adaptive capacity of *C. kousa* . The research centers on the current genome‐wide genetic diversity and population genetic structure of *C. kousa* , along with the alterations in its population genetic structure under future climate scenarios. The findings of this study will offer a crucial case reference for “the evolutionary dynamics of woody plants with restricted dispersal and significant ecological importance amidst climate change.” Moreover, they will provide valuable insights for formulating conservation strategies aimed at safeguarding the ecological and evolutionary potential of temperate forest species.

## 2 Materials and Methods

### 2.1 Sample Collection

The subject of this study is *C. kousa* within the Cornaceae family. During the period from 2022 to 2023, building upon prior research and referring to the databases of the National Specimen Information Infrastructure (NSII) of China (http://www.nsii.org.cn), the Chinese Virtual Herbarium (CVH, https://www.cvh.ac.cn), and the Global Biodiversity Information Facility (GBIF, https://www.gbif.org), extensive field investigations and samplings were conducted across the natural distribution range of the wild *C. kousa* species.

**FIGURE 1:**
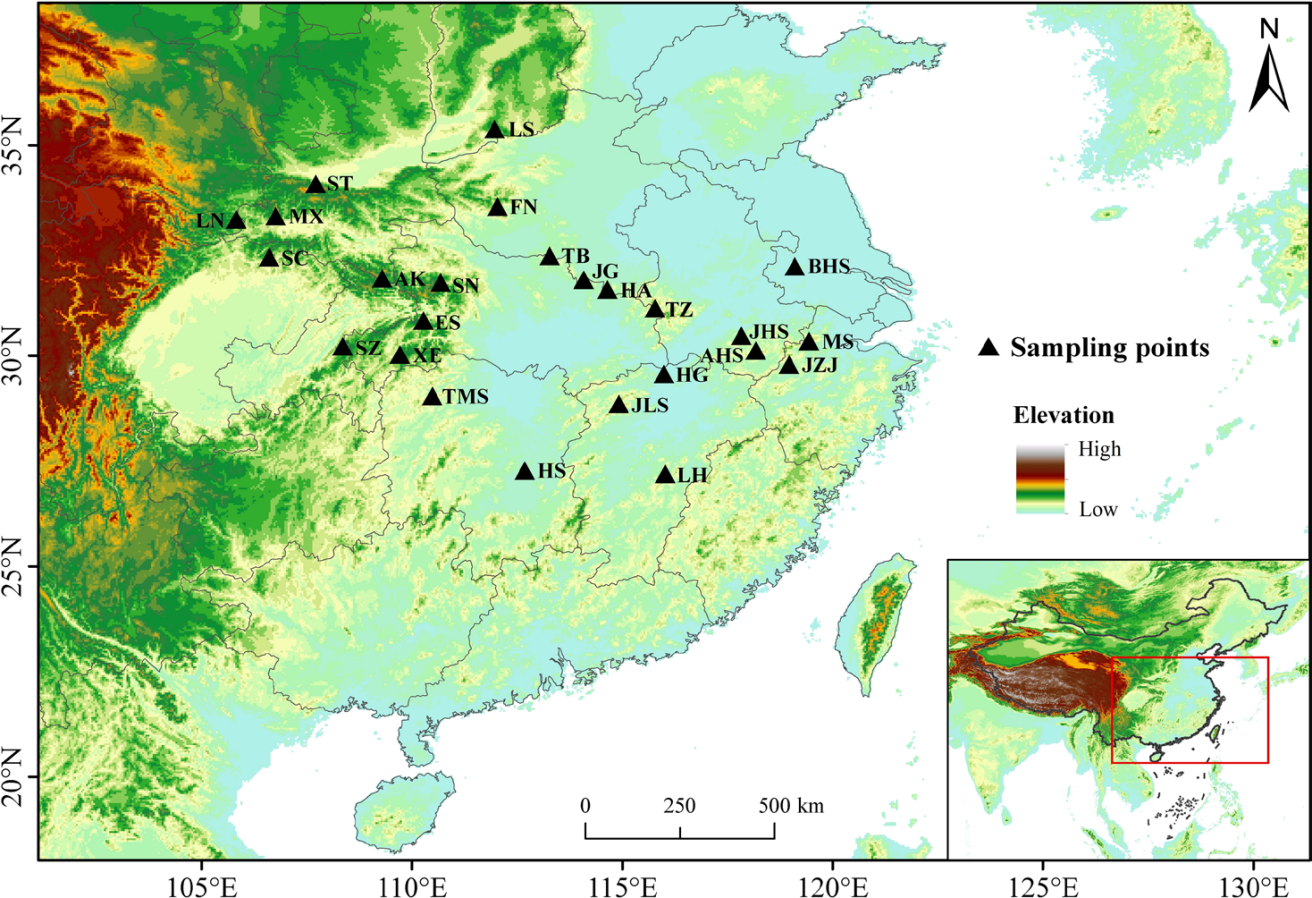
The distribution map depicting the sampling points of 25 populations of *Cornus kousa* investigated in this study is presented. The sampling locations are denoted by the black triangular symbols.

**FIGURE 2:**
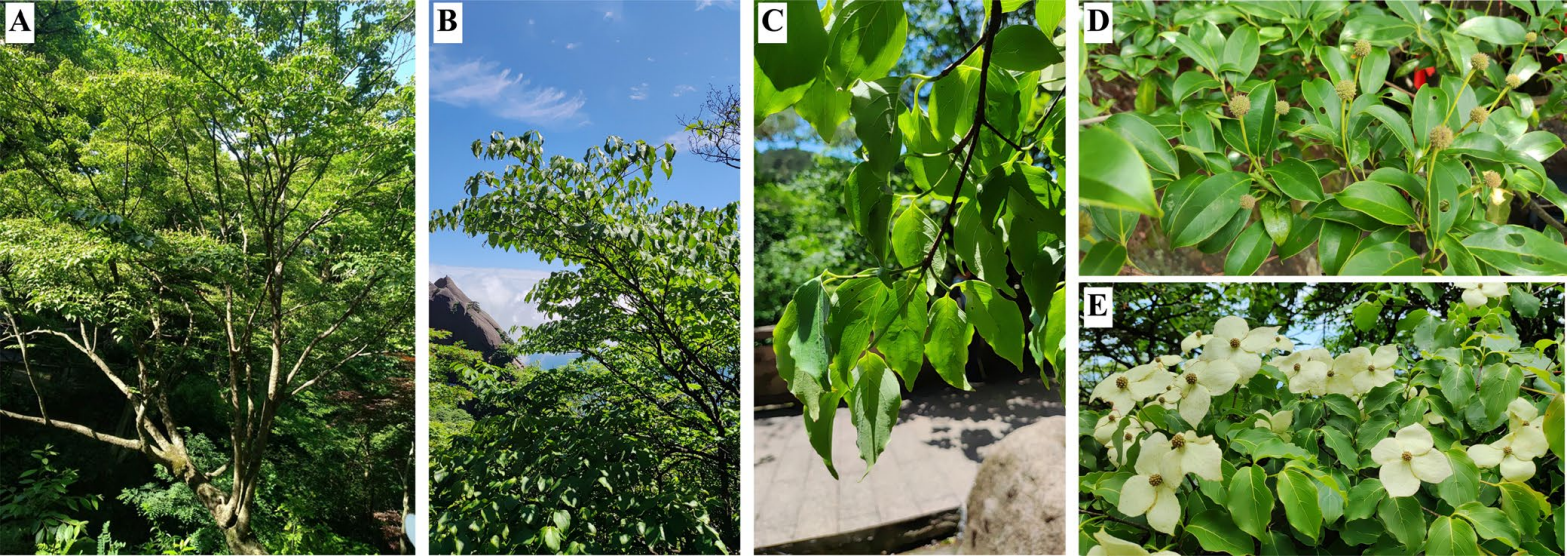
A comprehensive overview of the *Cornus kousa* collected during this study is provided. (A) The morphological characteristics of *C. kousa* . (B) The ecological habitats of *C. kousa* . (C) The leaves of *C. kousa* . (D) The flowers of *C. kousa* . (E) The bracts of *C. kousa* .

Sampling shall adhere to the following principles: (1) Sampling points should represent natural geographical populations. (2) A minimum distance of at least 50 m must be strictly maintained between individual specimens. (3) Comprehensive records of habitat information, longitude, latitude, and altitude should be meticulously documented, accompanied by visual records in the form of photographic documentation. (4) Healthy and freshly grown young leaves were collected and promptly desiccated using silica gel. The sampling points, morphological characteristics, habitat details, and geographical information of *C. kousa* in this study are presented in Figures 1 and 2 and Table S1. All samples were preserved in the ultra‐low‐temperature refrigerator (−80°C) at the School of Life Sciences, Nanchang University, for subsequent DNA extraction.

### 2.2 Genomic DNA Extraction and Construction of Genotyping‐By‐Sequencing (GBS) Library

Genomic DNA was isolated from the desiccated leaf specimens employing the Ezup Column Plant Genomic DNA Extraction Kit (Shanghai Sangong Bioengineering Co. Ltd.). All procedures were executed in strict accordance with the kit's instructions. The integrity of the extracted DNA was initially assessed via 1% agarose gel electrophoresis. Subsequently, the purity and concentration of the DNA were precisely determined using a Nanodrop 1000 spectrophotometer.

GBS was conducted on the DNA samples that satisfied the criteria. The concise workflow is delineated as follows: Samples with a total DNA quantity of at least 8.0 ng were selected. First, the DNA was digested using restriction enzymes. Subsequently, sequencing adapters were ligated. Ultimately, the GBS library was constructed through polymerase chain reaction (PCR). Sequencing was carried out on the Illumina HiSeq platform. The raw data generated from sequencing were subjected to filtration. Redundant adapters, low‐quality sequences, and undetermined bases were eliminated to acquire high‐quality clean data. The genome sequence of *C. florida* , a related species of *C. kousa* , was retrieved from the genome database of the National Center for Biotechnology Information (NCBI, https://www.ncbi.nlm.nih.gov/genome) and employed as the reference genome. The BWA software was utilized to align the clean reads with the reference genome (Li and Godzik 2006). Subsequently, the comparison facilitated by SAMtools is employed to sort and eliminate duplicates from the results (Li et al. 2009). Finally, VCFtools was used to rigorously filter the original set of SNPs (Danecek et al. 2011).

### 2.3 Genetic Diversity and Population Genetic Structure Analysis

Drawing upon the filtered SNP dataset, the genetic diversity parameters at both the population and species levels were computed through the utilization of GenAlEx 6.51 software (Peakall and Smouse 2006). These parameters encompass the allelic count (*Na*), the number of effective alleles (*Ne*), the anticipated heterozygosity (*He*), the observed heterozygosity (*Ho*), nucleotide diversity (*π*), and the Shannon diversity index (*I*).

The Structure 2.3.4 software is employed to deduce the genetic structure of the population, relying on the Bayesian clustering algorithm (Earl and VonHoldt 2012). The potential genetic groups (represented by the *K*‐value) are set to range from 2 to 25, and each *K*‐value is independently executed three times. For each run, the number of iterations in the Markov Chain Monte Carlo (MCMC) process amounts to 20,000, with the initial 10,000 iterations being discarded. By leveraging the online tool Structure Harvester (http://taylor0.biology.ucla.edu/struct_harvest), the optimal *K* value is ascertained in accordance with the Δ*K* method (Rosenberg 2003). Eventually, the outcomes of the repeated runs are amalgamated through the use of CLUMPP software (Jakobsson and Rosenberg 2007), and the final population genetic structure map is visualized via the employment of Distruct software (Pritchard et al. 2000).

### 2.4 Analysis of Climate Related Landscape Genomics

From the WorldClim database (https://worldclim.org), 19 bioclimatic factors (Bio1‐Bio19) and elevation data (Ele) (Table S2) were acquired for the present period (1970–2000), featuring a spatial resolution of 2.5 arc‐minutes. Variable with a correlation coefficient *R* < 0.7 were retained for subsequent analysis (Dormann et al. 2013). The 169 occurrence records were obtained from GBIF, CVH, and NSII after filtering for duplicates, cultivated plants, and spatial thinning to reduce autocorrelation. These variables, together with the 169 occurrence records, were used to build ensemble SDMs for the contemporary period (1970–2000) and for future scenarios using the Biomod2 package in R (Thuiller et al. 2009).

The POPs software was employed to simulate the dynamic alterations in the genetic structure under contemporary and future climate scenarios (Jay et al. 2015). Initially, the climate data under the RCP45 and RCP85 scenarios for the year 2070 (average for the period 2061–2080) were retrieved from the WorldClim database. Subsequently, ArcGIS was utilized to extract the values of the filtered climate variables corresponding to 25 sampling points. The SNP data, geographical coordinates, and climate data were jointly imported into POPs, and the range of the *K*‐value was defined. The operation was carried out using the MCMC algorithm (with 10,000 iterations and 2000 preburns) (Spiegelhalter et al. 2002). The optimal *K*‐value was selected in accordance with the principle of the minimum deviance information criterion (DIC), and the composition of the genetic clusters and their spatial distribution under different climate scenarios were visualized. In order to pinpoint populations that face an elevated risk of climate‐induced extirpation under future scenarios, we employed a precautionary strategy by setting a fitness threshold at 0.5. This value denotes the midpoint of the medium‐suitability spectrum (0.33–0.66) and serves as a more conservative demarcation than the high‐suitability boundary (0.66). Although there is no overarching standard for such thresholds, a value of 0.5 has been used as a suitability cutoff in previous climate change impact assessments (Collevatti et al. 2011; Lima et al. 2017).

To discern the genomic loci influenced by natural selection, we adopted a two‐step approach. First, the BayeScan 2.1 software was employed to pinpoint SNP loci that exhibited significantly greater differentiation among populations compared to those with a neutral background (Foll and Gaggiotti 2008). The false discovery rate (FDR) threshold was set at 0.1 to sieve out the significant outlier loci. Subsequently, the R‐package was utilized for the analysis (Frichot and Francois 2015). The filtered SNP data and the climate variable data of the sampling points were imported. The latent factor *K* was set to 4, and 10,000 iterations were run, with the first 5000 iterations being discarded. SNP loci with a *p*‐value less than 0.01 were recognized as candidate adaptive loci that were significantly correlated with climate factors.

## 3 Results

### 3.1 GBS Sequencing Results of *C. kousa*

In the present study, 348 wild specimens of *C. kousa* sourced from 25 natural populations were subjected to GBS. Subsequent to rigorous quality control measures, the sequencing data were refined to acquire high‐quality clean reads. The mean effective data rate reached 92.03%, and the mean base error rate stood at 0.03%. The assessment of sequencing quality revealed that Q20 (the proportion of bases with a quality value of at least 20) averaged 95.72%, Q30 (the proportion of bases with a quality value of at least 30) averaged 89.04%, and the guanine‐cytosine (GC) content averaged 38.35%. These findings suggest that the data quality was trustworthy and apt for subsequent analyses (Table S3).

The analysis of enzyme digestion efficiency statistics indicated that the enzyme digestion efficiency of all specimens spanned from 94.8% to 99.6%, and the completeness rate of enzyme digestion varied from 81.7% to 96.0%. This implies that the enzyme digestion reaction was thorough during the library construction process, which was favorable for the generation of subsequent tag sequences (Table S4). The statistics regarding tag fragments demonstrated that the total quantity of tags in each sample ranged between 2,012,360 and 2,840,209. Among these, the number of tags with a length of at least 4 bases ranged from 164,779 to 224,456, and the average sequencing depth ranged from 11,471 to 12,654, suggesting a high level of tag coverage (Table S5).

The high‐quality clean reads were aligned against the reference genome ( *C. florida* ). The alignment rate fluctuated within the range of 62.14%–88.36%, and the mean sequencing depth spanned from 11.23 to 15.5. The minimum coverage at a depth of 1× was 20.47%, while the maximum reached 29.99%. The coverage at a depth of 4× was between 12.56% and 22.8% (Table S6). These findings indicated that the sequencing data exhibited a remarkable congruence with the reference genome, thereby laying a robust groundwork for subsequent SNP detection and genetic analysis.

### 3.2 Genetic Diversity of *C. kousa* Under Current and Future Climate

Leveraging the 5939 high‐quality SNP loci acquired through GBS, the genetic diversity of 25 *C. kousa* populations under the present climatic conditions was scrutinized using GenAlEx software. The genetic diversity indices of these 25 populations are presented as follows: the count of *Na* per locus stands at 2.421, the *Ne* is 1.820, the *I* is 0.601, the *He* is 0.390, and the *Ho* reaches 0.693 (Table 1). Collectively, these indicators uniformly suggest that the genetic diversity level of *C. kousa* remains elevated under the current climate regime.

**TABLE 1:** Genetic diversity of 25 *Cornus kousa* populations calculated by GenALEx.

Population	Sample number	Na	Ne	I	He	Ho
AHS	15	2.412	1.810	0.594	0.387	0.693
AK	15	2.369	1.803	0.590	0.385	0.691
BHS	11	2.414	1.829	0.606	0.392	0.692
ES	15	2.460	1.822	0.604	0.390	0.692
FN	15	2.509	1.829	0.609	0.393	0.694
HA	15	2.500	1.837	0.613	0.395	0.693
HG	15	2.539	1.837	0.616	0.395	0.695
HS	15	2.471	1.821	0.603	0.390	0.694
JG	15	2.467	1.820	0.602	0.390	0.694
JHS	15	2.514	1.829	0.610	0.393	0.694
JLS	4	2.429	1.819	0.600	0.389	0.693
JZJ	14	2.035	1.776	0.562	0.376	0.682
LH	15	2.488	1.826	0.607	0.392	0.693
LN	15	2.450	1.817	0.601	0.389	0.692
LS	14	2.102	1.819	0.584	0.387	0.693
MS	15	2.357	1.805	0.590	0.386	0.692
MX	15	2.490	1.825	0.607	0.391	0.692
SC	15	2.492	1.823	0.606	0.391	0.694
SN	15	2.420	1.821	0.601	0.390	0.694
ST	15	2.347	1.812	0.592	0.387	0.693
SZ	15	2.397	1.818	0.599	0.389	0.694
TB	15	2.521	1.836	0.613	0.394	0.694
TMS	15	2.417	1.816	0.598	0.389	0.694
TZ	15	2.542	1.840	0.616	0.396	0.694
XE	5	2.390	1.811	0.593	0.386	0.692
Mean	—	2.421	1.820	0.601	0.390	0.693

As illustrated in Figure S1, the RCP45 scenario gives rise to minor alterations in the habitat and features core stable regions that are well‐conserved. In contrast, the RCP85 scenario leads to more substantial modifications. The ensemble model demonstrated dependable predictive capabilities (Table S7; mean AUC = 0.873 for the current climate, 0.698 for RCP45, and 0.710 for RCP85). To delve into the potential influence of future climate change on genetic diversity, the fitness values of diverse populations under the current and future climate scenarios (RCP45 and RCP85) were extracted via ArcMap. Using a fitness value of 0.500 as the threshold, in the future RCP45 scenario, the adaptability values of JLS (0.308), FN (0.441), and MS (0.498) were below the pre‐set threshold. After elimination, 22 populations were retained. In the future RCP85 scenario, six populations, namely JLS (0.305), ST (0.439), FN (0.447), MX (0.489), MS (0.496), and TZ (0.497), had values lower than the established threshold, and 19 populations remained after the culling process. The SNP data of these remaining populations were re‐examined to assess genetic diversity. The findings indicated that in the future RCP45 scenario, the average *I* was 0.601, and the mean *He* was 0.390. In the future RCP85 scenario, the average value of *I* was 0.601, and the average *He* was 0.390 (Table 2). When compared with the genetic diversity indices under the current climate (*I* = 0.601, *He* = 0.390), the genetic diversity of the *C. kousa* population did not exhibit a substantial change under the two future climate scenarios. This implies that climate change may not result in a significant loss of genetic diversity in the short term.

**TABLE 2:** Genetic diversity and fitness of 25 *Cornus* *kousa* populations.

Population	Future RCP45		Future RCP85		Fitness		
	I	He	I	He	Current	RCP45	RCP85
AHS	0.594	0.387	0.594	0.387	0.896	0.847	0.790
AK	0.590	0.385	0.590	0.385	0.825	0.552	0.562
BHS	0.606	0.392	0.606	0.392	0.377	0.658	0.656
ES	0.604	0.390	0.604	0.390	0.715	0.713	0.666
FN	—	—	—	—	0.530	0.441	0.447
HA	0.613	0.395	0.613	0.395	0.934	0.791	0.789
HG	0.616	0.395	0.616	0.395	0.728	0.733	0.727
HS	0.603	0.390	0.603	0.390	0.901	0.944	0.952
JG	0.602	0.390	0.602	0.390	0.892	0.890	0.723
JHS	0.610	0.393	0.610	0.393	0.671	0.582	0.630
JLS	—	—	—	—	0.231	0.308	0.305
JZJ	0.571	0.381	0.571	0.381	0.930	0.930	0.947
LH	0.607	0.392	0.607	0.392	0.624	0.628	0.718
LN	0.601	0.389	0.601	0.389	0.695	0.787	0.789
LS	0.584	0.387	0.584	0.387	0.789	0.650	0.746
MS	—	—	—	—	0.348	0.498	0.496
MX	0.607	0.391	—	—	0.570	0.622	0.489
SC	0.606	0.391	0.606	0.391	0.804	0.724	0.799
SN	0.601	0.390	0.601	0.390	0.902	0.908	0.944
ST	0.592	0.387	—	—	0.276	0.520	0.439
SZ	0.599	0.389	0.599	0.389	0.548	0.753	0.811
TB	0.613	0.394	0.613	0.394	0.798	0.556	0.568
TMS	0.598	0.389	0.598	0.389	0.823	0.729	0.899
TZ	0.616	0.396	—	—	0.300	0.601	0.497
XE	0.593	0.386	0.593	0.386	0.930	0.930	0.943
Mean	0.601	0.390	0.601	0.390	—	—	—

### 3.3 Genetic Structure of *C. kousa* Under Current and Future Climate

**FIGURE 3:**
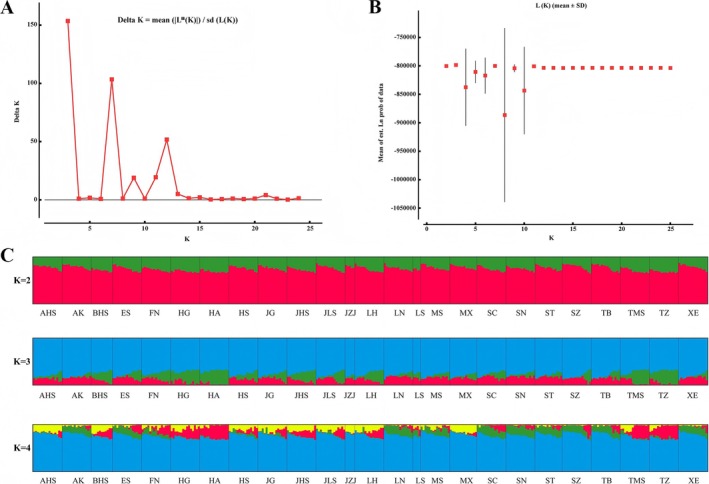
Genetic structure diagram of 25 *Cornus kousa* populations simulated by Structure and Structure Harvester website based on SNP data. (A) K and the mean probability of Ln*P*(*D*). (B) ∆*K* line chart. (C) Genetic cluster maps of *C. kousa* with different *K* values (*K* = 2, 3, 4) constructed by CLUMPP and distruct.

SNP data from 25 populations of *C. kousa* , encompassing 348 individuals, were subjected to analysis using the Structure software. The optimal number of genetic groups was ascertained via the Δ*K* method. When *K* = 3, the Δ*K* value reached its maximum, suggesting that these populations could be partitioned into three genetic clusters (Figure 3A,B). At *K* = 3, the genetic architecture was predominantly characterized by the blue gene pool, which accounted for over 50%. In contrast, the proportions of the green and red gene pools were relatively meager. Specifically, populations such as HA, TZ, HG, TMS, BHS, JHS, LH, JLS, FN, and HS exhibited a higher proportion of the green gene pool. Conversely, populations including SN, LN, ST, SC, AK, MS, XE, TB, LS, JZJ, AHS, JG, and SZ had a higher proportion of the red gene pool (Figure 3C). When *K* = 2, the population was bifurcated into red and green subgroups; however, further discrimination between them proved to be arduous. When *K* = 4, the clustering pattern bore resemblance to that observed at *K* = 3. Collectively, the results indicated that a certain degree of genetic admixture existed among the *C. kousa* populations. Nevertheless, three genetic clusters emerged as the dominant features.

**FIGURE 4:**
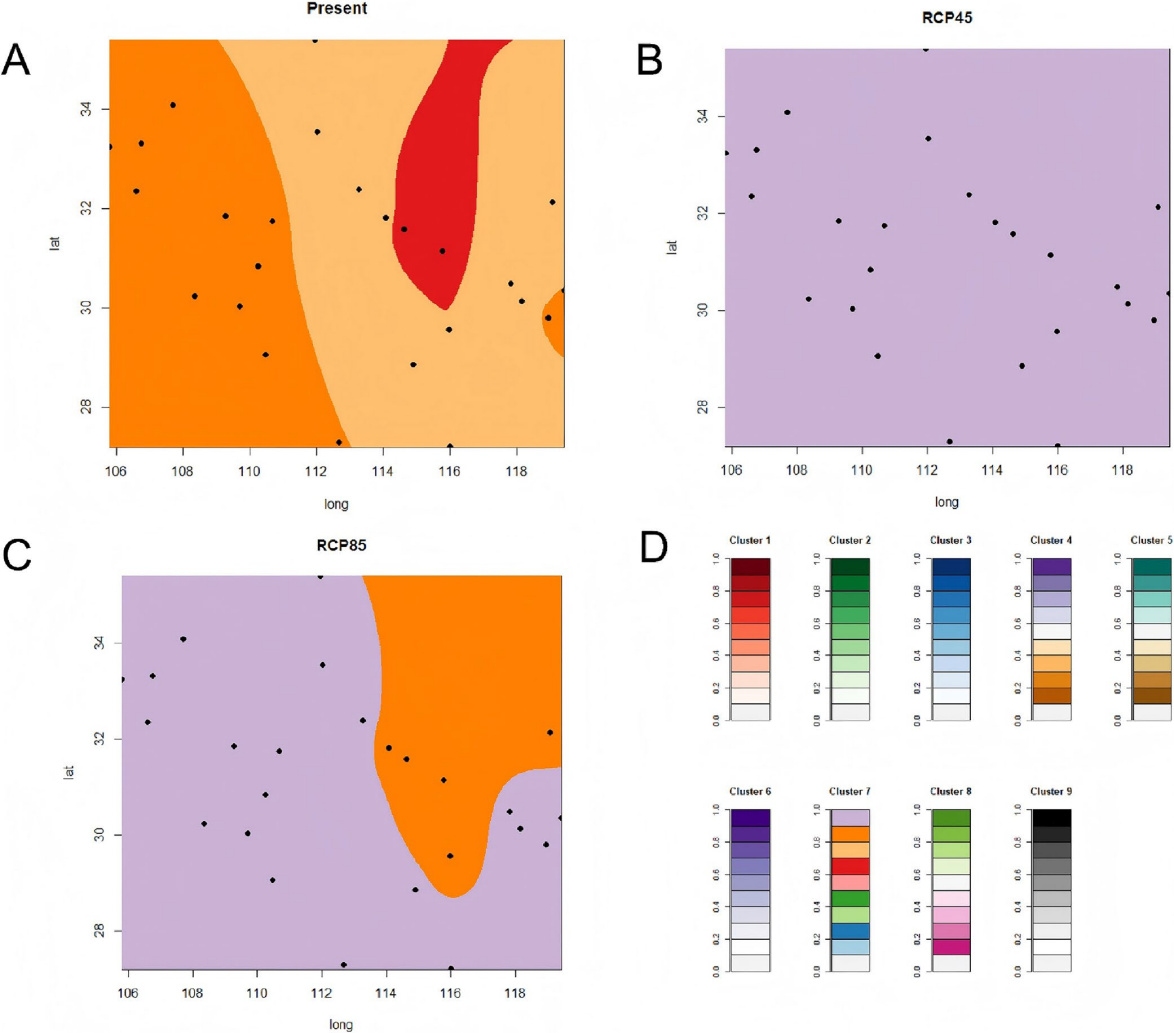
The spatial distribution maps of genetic clusters of 25 *Cornus kousa* populations under current and two future climate scenarios simulated by POPs. (A) Current climate. (B) Future RCP45 climate scenario. (C) Future RCP85 climate scenario. (D) Different genetic clusters were represented by different colors.

**FIGURE 5:**
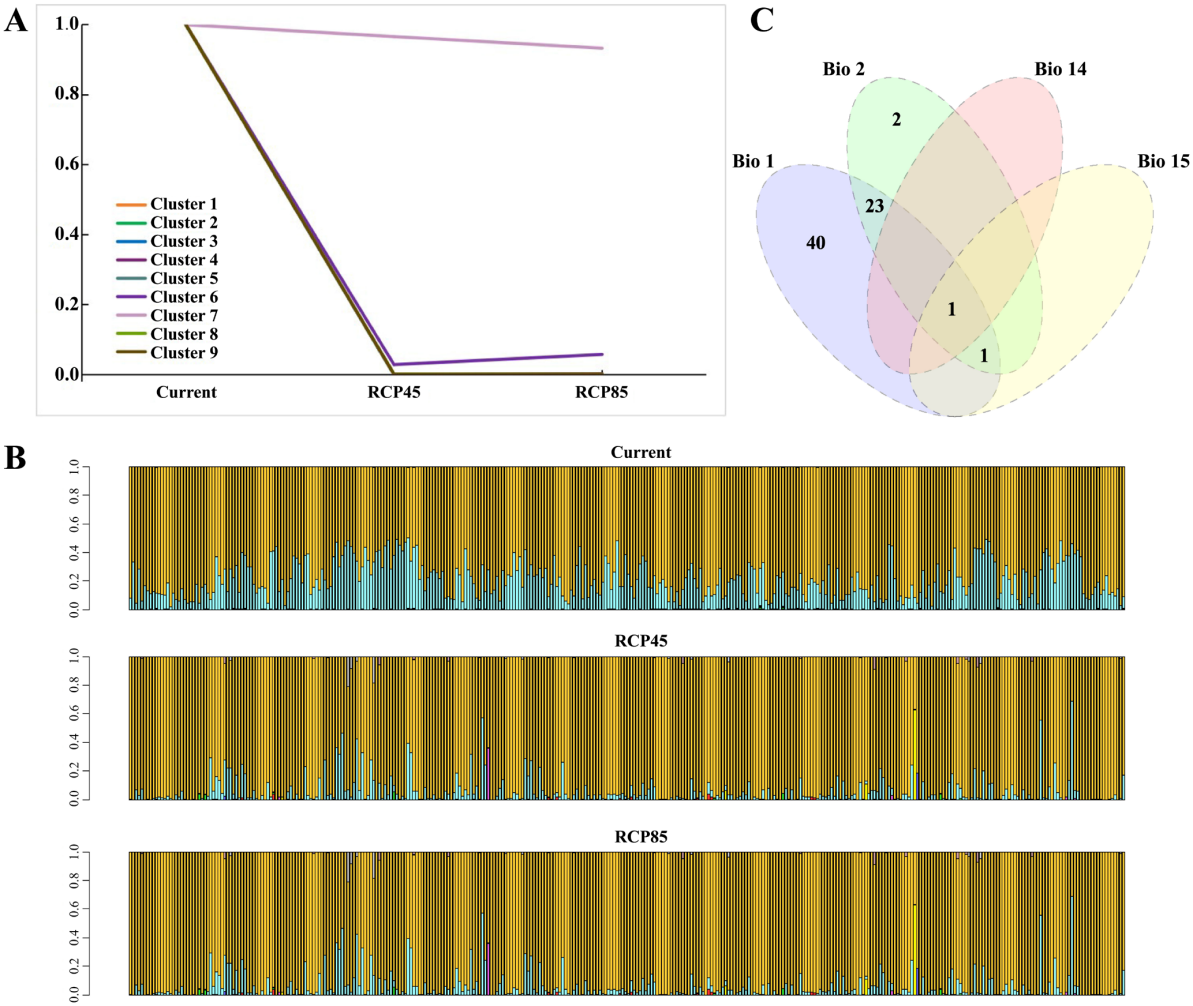
Genetic structure response of *Cornus kousa* to future climate scenarios. (A) Dynamic ancestor changes for nine genetic clusters simulated by POPs. (B) Genetic structure plots of *C. kousa* simulated by POPs under current, future RCP45 and RCP85 climate scenarios. (C) Sixty‐seven SNP loci associated with climate variables detected by BayeScan.

To more comprehensively uncover the influence of climate change on the genetic structure, the POPs software was employed to integrate genetic data and climate‐related variables for in‐depth analysis. When *K* = 9, the DIC attains its minimum value (126,395). Moreover, the correlation between the inferred mixing coefficient and climate covariates reaches as high as 0.979, which implies that the model boasts a high degree of prediction accuracy (Table S8). Additionally, the simulation outcomes of the POPs software demonstrated that the 25 populations of *C. kousa* were partitioned into multiple climatic genetic clusters under the present climate conditions. Under the two future climate scenarios, namely RCP45 and RCP85, 93.6% of 25 *C. kousa* populations are categorized into a common genetic cluster (Cluster 7) (Figure 4). Generally speaking, under the two future climate scenarios, the genetic cluster of *C. kousa* is likely to be lost, resulting in the homogenization of genetic diversity. This phenomenon is more pronounced under the RCP45 scenario. Under the RCP85 scenario, the correlation of Cluster 6 increased. In brief, the variation across all clusters under the RCP85 scenario is greater than that under the RCP45 scenario (Figure 5A). Although the results indicated that climate change exerts a significant impact on the spatial genetic structure of *C. kousa* , the responses of different genetic clusters vary under the two future climate scenarios. Specifically, the genetic structure under the current climate can be classified into three gene pools. The yellow genetic cluster predominates, yet the blue genetic cluster also constitutes a certain proportion. Nevertheless, by the year 2070, the genetic structure of *C. kousa* will have undergone substantial alterations under the RCP45 and RCP85 climate scenarios, with the yellow genetic cluster emerging as the dominant one. The aforementioned results also suggest that the genetic structure of *C. kousa* will become more homogeneous under future climate change (Figure 5B).

### 3.4 Effects of Climatic Factors on Genome Selection of *C. kousa*

The BayeScan software was employed to detect the outliers among SNP loci. In total, 72 outliers were discerned, which constituted 1.21% of the overall SNP loci (Table S9). Subsequently, the LFMM was utilized to analyze the correlation between these outliers and climatic factors. Remarkably, 67 of these outliers exhibited a significant correlation with climatic variables (Figure 5C). Specifically, 65 loci were associated with the annual mean temperature (Bio 1), accounting for 90.3% of the total outliers. There were 27 loci linked to the mean diurnal range (Bio 2). Two loci were related to the coefficient of variation (Bio 15), and one locus was associated with the precipitation of the driest month (Bio 14). These findings suggest that the annual mean temperature stands as the most pivotal climatic factor propelling the genome‐wide adaptive evolution of *C. kousa* . Moreover, the mean diurnal range and coefficient of variation also exert an influence on its genetic variation to a certain degree. This implies that *C. kousa* is plausibly particularly sensitive to future temperature fluctuations, and its adaptive evolutionary process is intricately associated with the selective pressure imposed by climatic factors.

## 4 Discussion

### 4.1 Dynamic Responses of the Genetic Diversity and Genetic Structure of *C. kousa* Amidst Climate Change

Genetic diversity serves as the fundamental cornerstone that empowers species to withstand environmental pressures and sustain their survival and evolutionary processes (Dar et al. 2024; Chung et al. 2023). Upon assessing crucial genetic parameters, the research discerned that the *C. kousa* exhibited a remarkable degree of genetic diversity at the species level (*I* = 0.601, *He* = 0.390). This level of genetic diversity was notably elevated compared to that of *C. officinalis* (*I* = 0.241, *He* = 0.361) and *Davidia involucrata* (*I* = 0.220, *He* = 0.335) (Li et al. 2012; Luo et al. 2011). The comparatively elevated genetic diversity of *C. kousa* can be primarily ascribed to its distinctive biological traits: sexual reproduction that hinges on cross‐pollination and a perennial life cycle. These attributes efficaciously foster the long‐term accumulation and conservation of genetic variation (Brito et al. 2016). Nonetheless, impending climate change is poised to present a grave peril to the population of this species. Model projections indicate that under the RCP45 and RCP85 scenarios, three and six populations of *C. kousa* , respectively, will confront the risk of regional extinction. Although the genetic diversity index computed from genome‐wide SNP data does not suggest a significant overall decline, the disappearance of local populations will inexorably result in the irrevocable loss of their distinctive genetic variations (Hoffmann et al. 2021). This tendency aligns with the research findings in other ecosystems across the globe. For instance, under the identical climate scenario, *Tabebuia aurea* is anticipated to experience the loss of 7 and 16 populations, precipitating a drastic decline in genetic diversity (Lima et al. 2017). In the case of Brazil's *Caryocar brasiliense* , the fitness of as many as 10 populations is projected to be less than 0.5 under future climate conditions, signifying that its genetic diversity might encounter the risk of an abrupt reduction (Collevatti et al. 2011).

Beyond the decline in population and genetic variation, the simulation outcomes of this study have also uncovered more profound alterations in the genetic structure itself. The present analysis indicates that the *C. kousa* population can be partitioned into three markedly distinct gene pools (*K* = 3). Nevertheless, via POPs analysis, it has been ascertained that its genetic structure is fated to undergo substantial simplification: the genetic differentiation among populations will gradually diminish and ultimately coalesce into a dominant genetic cluster. The underlying impetus for this change is the increasingly severe extreme climate, encompassing notable warming, seasonal intensification, and a reduction in dry‐season precipitation in specific regions. These factors compel the populations adapted to different microenvironments to confront the convergent selection pressure. This genetic homogeneity will gravely undermine the population's evolutionary resilience to environmental fluctuations (Benning et al. 2023). The constraint of a species' dispersal capacity further exacerbates the genetic risk. The seeds of *C. kousa* predominantly depend on rodents for short‐range dissemination, and its natural propagation scope is circumscribed. Under the dual pressures of climate change and habitat fragmentation, gene flow among populations will be notably impeded. Although the cross‐pollination mechanism and the genetic variation inherent in its perennial traits offer a buffer, in the face of the rapid pace of climate change, natural adaptation alone may prove insufficient to guarantee its survival (Lloyd et al. 2018; Skogen et al. 2019).

Moreover, the reproductive biology of *C. kousa* can exert a crucial influence on its genomic responses to climate change. Seeds of this species are in a state of physiological dormancy, attributable to inhibitors present in the endosperm. To initiate germination, they necessitate either cold stratification or hormonal treatment (Fu et al. 2013). As winter temperatures escalate, the chilling requirements for these seeds may not be adequately met. This situation has the potential to curtail recruitment, particularly, among populations at low elevations or in the southern margins of its range (Walck et al. 2011). Clonal propagation can, for a time, sustain local genets and serve as a buffer against genetic erosion. Nevertheless, the persistence of clonal reproduction reduces the occurrence of sexual recombination. Consequently, it hampers the generation of novel allelic combinations that are essential under novel selective pressures. Over multiple generations, this reproductive strategy may limit the species' adaptive evolutionary potential. This concern is consistent with our prediction of genetic homogenization under future climate scenarios (Vallejo‐Marin et al. 2010). Pollen and seeds serve as the crucial vectors of gene flow. Their restricted dispersal will profoundly reconfigure the genetic architecture of the population and ultimately imperil the long‐term viability of the species (Browne and Karubian 2018; Escobar et al. 2023). Our SDM projections further suggest that, instead of facing complete extinction, *C. kousa* as a whole is likely to respond through range shifts. Nevertheless, the species' restricted dispersal ability may significantly impede its capacity to colonize newly suitable habitats. This could result in temporary local extinctions, even when the climatic conditions become propitious in other regions.

### 4.2 Effects of Climatic Factors on Genome Selection of *C. kousa*

The remarkable diversity of the Earth's environment has given rise to an extensive spectrum of climate‐associated selection pressures. This has compelled plants to generate adaptive variations at the genomic level. These evolutionary imprints are conserved in the guise of “climate‐related genetic loci” (Li et al. 2021). A multitude of studies have indicated that temperature and precipitation stand as the core environmental elements dictating the adaptive evolution of forest species (Sampedro and Alía 2023; Ahrens et al. 2019).

In the present study, BayeScan and LFMM were employed to conduct a comprehensive whole‐genome scan of *C. kousa* . As a result, 67 SNP loci that were significantly correlated with key climate factors were identified. The allele frequencies of these loci exhibited a systematic alteration along the climate gradient, thereby offering direct molecular evidence for the adaptive evolution of the species. The analysis revealed that the influence of the annual mean temperature (Bio 1) was overwhelmingly predominant. Specifically, over 60 SNP loci were associated with it, constituting more than 90% of the total number of aberrant loci. This outcome strongly implies that during the historical evolution of *C. kousa* , the annual mean temperature (Bio 1) has served as the primary selection pressure shaping its genomic variation. Such observations are of a general nature. For instance, in the Mojave Desert of the United States, the annual mean temperature has also been demonstrated to be the principal factor propelling the genetic differentiation of *Ephedra nevadensis* and *Sphaeralcea ambigua* (Shryock et al. 2016). Temperature‐related factors also play a crucial role in determining the distribution of *Platycladus orientalis* (Jia et al. 2019). Moreover, the diurnal temperature range (Bio 2) was found to be correlated with 27 SNP loci. This suggests that the fluctuations in temperature between day and night have exerted a significant influence on the local adaptation of the species. The coefficient of variation (Bio 15) exhibits an association with two genetic loci, which mirrors the potential ramifications of seasonal precipitation variability on genomic evolution. Despite the meager proportion of loci associated with precipitation, the identified correlations with precipitation seasonality (Bio15) and precipitation of the driest month (Bio14) imply that water availability can serve as a secondary selective determinant. In areas where future warming is accompanied by more pronounced seasonal precipitation patterns or diminished dry‐season precipitation, temperature and water stress may interact synergistically to mold adaptive genomic variation. For example, alleles that confer an advantage under high‐temperature conditions might lead to reduced fitness during drought, giving rise to trade‐offs that could alter the frequency of the scant precipitation‐associated loci that have been detected. Our findings, therefore, issue a cautionary note: future adaptive responses should not be deduced solely from temperature data. Precipitation regimes, although statistically less prominent in terms of associated loci, may substantially influence temperature‐driven selection (Anderson and Song 2020). For instance, a substantial number of loci associated with drought tolerance were identified in the genome of *Alnus glutinosa* (Korte and Farlow 2013). Additionally, the analyses of endangered species like *Cephalotaxus oliveri* and *Abies alba* also revealed that their genomes harbored a multitude of adaptive loci linked to temperature and precipitation (Wang et al. 2016; Roschanski et al. 2016). A recent inquiry has revealed that species possessing relatively narrow temperature niche breadths are more prone to extinction amidst climate change (Malanoski et al. 2024). Regarding *C. kousa* , the annual mean temperature (Bio 1) might assume an extraordinarily pivotal role in its evolutionary trajectory. Confronted with the looming threat of climate change, as a substantial upsurge in global temperatures is projected, the genomic adaptive evolution of *C. kousa* in future environmental scenarios merits more profound and comprehensive exploration.

### 4.3 The Future of *C. kousa* From the Perspective of Conservation Biogeography and Conservation Biology

Against the backdrop of global climate change, the conservation of species transcends the mere safeguarding of biodiversity. It also encompasses the sustenance of the functional stability of ecosystems and the preservation of the inherent evolutionary potential within species (Gaitan‐Espitia and Hobday 2020; Schlaepfer and Lawler 2022). *C. kousa* , a quintessential woody plant in the subtropical to warm‐temperate forest ecosystems of China, plays a pivotal ecological role. Its survival and evolution are not merely linked to the species itself; rather, they hold paramount significance for the conservation of regional biodiversity and the equilibrium of ecosystems.

From the vantage point of conservation biology, this research delved into the latent risks confronting *C. kousa* under future climate scenarios and furnished a scientific foundation for devising efficacious conservation strategies. By means of the profound integration of the SDM and genetic structure analysis, this study ascertained that under future climate scenarios, *C. kousa* exhibited a pronounced tendency toward genetic homogeneity. This diminution in genetic diversity might enfeeble the population's capacity to adapt to environmental alterations and undermine its evolutionary resilience (DeWoody et al. 2021; Frei et al. 2023). As a tree species with restricted dispersal capabilities, gene flow among *C. kousa* populations is circumscribed. Moreover, the anticipated future climate warming is projected to further exacerbate the fragmentation of its habitat, intensify genetic isolation, and heighten the risk of local extinction.

Therefore, from a biogeographical standpoint, precedence ought to be accorded to enhancing the continuity of its distribution range and augmenting the ecological connectivity between extant habitats and future climate refuges. In the realm of conservation biology, *C. kousa* is by no means an isolated species; rather, it is a keystone species within the forest ecosystem it inhabits. It makes a significant contribution to the forest structure and nutrient cycling, as well as the provision of food and habitats for wild animals (Shackleton et al. 2018; He et al. 2021). The decline of the *C. kousa* population induced by climate change may set off a chain reaction, impact the symbiotic plant and animal species, and ultimately disrupt the ecological balance of the ecosystem. This study revealed that the genetic diversity of *C. kousa* remained relatively high under future climate conditions, indicating that it still possessed a certain degree of adaptive potential. Nevertheless, the observed trend of genetic structure simplification implies that the natural recovery capacity should not be overestimated. Consequently, conservation strategies should center on safeguarding the genetic integrity of existing populations and mitigating the inbreeding depression and genetic drift resulting from habitat fragmentation.

The robust association of *C. kousa* populations with mountain ranges (such as the Dabie, Qinling, and Wuyi Mountains) indicates that these topographically intricate regions have, throughout history, functioned as climatic refuges. They have shielded populations from extreme temperature oscillations and preserved relatively stable moisture conditions (Dobrowski 2011). In the face of future warming scenarios, these montane refuges could once again prove to be of paramount importance. They may potentially host microclimates that remain conducive to the species' survival, even as the surrounding lowlands turn inhospitable. Consequently, effective conservation planning ought to prioritize the maintenance of landscape connectivity among these mountain refuges (Heller and Zavaleta 2009). This will facilitate gene flow and enable the species to shift its range in response to climate change.

## 5 Conclusions

In summary, this study comprehensively unveiled the multi‐faceted impacts of climate change on the genetic adaptability of *C. kousa* . Despite the fact that the overall genetic diversity of *C. kousa* did not exhibit a marked decline under future climate scenarios, its genetic structure displayed a pronounced trend toward homogenization. This structural simplification propelled by convergent selection pressure will severely undermine the population's capacity for adaptive differentiation. Genome analysis further corroborated that the temperature factor played a preeminent role in the selection process, whereas the selection signal of the precipitation factor was feeble. This indicates that temperature adaptability represents the pivotal evolutionary strategy for the survival of *C. kousa* . Evidently, climate change has placed *C. kousa* in the predicament of genetic structure simplification, which will pose a fundamental jeopardy to the long‐term survival of the species by diminishing its adaptability. In light of this, the conservation of subtropical forests should center on the in situ conservation of temperature‐sensitive populations, the establishment of habitat corridors to sustain gene flow, and the incorporation of climate‐related loci into the genetic assessment framework of assisted migration.

## Funding

This research endeavor received backing from the National Natural Science Foundation of China, under the grant number 32160053.

## Conflicts of Interest

The authors declare no conflicts of interest.

## Supporting information


**Figure S1:** The distribution changes of *C. kousa* under two future climate scenarios simulated by SDM Toolbox.
**Table S1:** The geographical locations and number of samples of *Cornus kousa* populations in the study.
**Table S2:** Summary table of 20 environmental factors used in the study.
**Table S3:** Genotyping‐by‐sequencing (GBS) outcomes of 348 samples of *Cornus kousa* sourced from 25 natural populations.
**Table S4:** A statistical tabulation presenting the results of enzymatic digestion for 348 samples of *Cornus kousa* procured from 25 natural populations.
**Table S5:** A statistical table presenting the tag fragments of 348 samples of *Cornus kousa* sourced from 25 natural populations.
**Table S6:** A statistical tabulation detailing the comparison between the reads and the reference genome of 348 samples of *Cornus kousa* sourced from 25 natural populations.
**Table S7:** Evaluation indices (AUC, Kappa, TSS) for the potential distribution areas of *Cornus kousa* under current and future (RCP45, RCP85, 2070) climate scenarios, based on the ensemble model.
**Table S8:** The Deviance Information Criterion (DIC) value and the correlation associated with the simulation of POPs.

## Data Availability

The filtered SNP dataset for *C. kousa* , comprising 5939 high‐quality loci for 348 individuals, is publicly available in the Dryad Digital Repository (https://doi.org/10.5061/dryad.hdr7sqvx6).

## References

[eva70281-bib-0001] Ahrens, C. W. , M. Byrne , and P. D. Rymer . 2019. “Standing Genomic Variation Within Coding and Regulatory Regions Contributes to the Adaptive Capacity to Climate in a Foundation Tree Species.” Molecular Ecology 28, no. 10: 2502–2516. 10.1111/mec.15092. 30950536

[eva70281-bib-0002] Anderson, J. , and B. H. Song . 2020. “Plant Adaptation to Climate Change‐Where Are we?” Journal of Systematics and Evolution 58, no. 5: 533–545. 10.1111/jse.12649. 33584833 PMC7875155

[eva70281-bib-0003] Asif, R. H. 2023. “A Review of the Global Climate Change Impacts, Adaptation Strategies, and Mitigation Options in the Socio‐Economic and Environmental Sectors.” Journal of Environmental Science and Economics 2, no. 3: 36–58. 10.56556/jescae.v2i3.587.

[eva70281-bib-0004] Benning, J. W. , A. Faulkner , and D. A. Moeller . 2023. “Rapid Evolution During Climate Change: Demographic and Genetic Constraints on Adaptation to Severe Drought.” Proceedings. Biological Sciences 290, no. 1998: 20230336. 10.1098/rspb.2023.0336. 37161337 PMC10170215

[eva70281-bib-0005] Bontrager, M. , and A. L. Angert . 2018. “Gene Flow Improves Fitness at a Range Edge Under Climate Change.” Evolution Letters 3, no. 1: 55–68. 10.1002/evl3.91. 30788142 PMC6369935

[eva70281-bib-0006] Brito, V. L. G. , G. M. Mori , B. B. Z. Vigna , M. Azevedo‐Silva , A. P. Souza , and M. Sazima . 2016. “Genetic Structure and Diversity of Populations of Polyploid *Tibouchina pulchra* Cogn. (Melastomataceae) Under Different Environmental Conditions in Extremes of an Elevational Gradient.” Tree Genetics & Genomes 12: 101. 10.1007/s11295-016-1059-y.

[eva70281-bib-0007] Browne, L. , and J. Karubian . 2018. “Habitat Loss and Fragmentation Reduce Effective Gene Flow by Disrupting Seed Dispersal in a Neotropical Palm.” Molecular Ecology 27, no. 15: 3055–3069. 10.1111/mec.14765. 29900620

[eva70281-bib-0008] Caye, K. , B. Jumentier , J. Lepeule , and O. François . 2019. “LFMM 2: Fast and Accurate Inference of Gene‐Environment Associations in Genome‐Wide Studies.” Molecular Biology and Evolution 36, no. 4: 852–860. 10.1093/molbev/msz008. 30657943 PMC6659841

[eva70281-bib-0009] Chen, W. 2018. Preliminary Study on Phylogeography and Landscape Genetics of *Cornus kousa* subsp. chinensis. Nanchang University.

[eva70281-bib-0010] Chung, M. Y. , J. Merila , J. L. Li , et al. 2023. “Neutral and Adaptive Genetic Diversity in Plants: An Overview.” Frontiers in Ecology and Evolution 11: 1116814. 10.3389/fevo.2023.1116814.

[eva70281-bib-0011] Collevatti, R. G. , J. C. Nabout , and J. A. F. Diniz‐Filho . 2011. “Range Shift and Loss of Genetic Diversity Under Climate Change in *Caryocar brasiliense* , A Neotropical Tree Species.” Tree Genetics & Genomes 7: 1237–1247. 10.1007/s11295-011-0409-z.

[eva70281-bib-0012] Cruzan, M. B. , and E. C. Hendrickson . 2020. “Landscape Genetics of Plants: Challenges and Opportunities.” Plant Communications 1, no. 6: 100100. 10.1016/j.xplc.2020.100100. 33367263 PMC7748010

[eva70281-bib-0013] Cushman, S. A. , A. J. Shirk , G. T. Howe , M. A. Murphy , R. J. Dyer , and S. Joost . 2018. “Editorial: The Least Cost Path From Landscape Genetics to Landscape Genomics: Challenges and Opportunities to Explore NGS Data in A Spatially Explicit Context.” Frontiers in Genetics 9: 215. 10.3389/fgene.2018.00215. PMC6018102 29971091

[eva70281-bib-0014] Danecek, P. , A. Auton , G. Abecasis , et al. 2011. “The Variant Call Format and VCFtools.” Bioinformatics 27, no. 15: 2156–2158. 10.1093/bioinformatics/btr330. 21653522 PMC3137218

[eva70281-bib-0015] Dar, B. A. , A. A. Al‐Doss , A. M. Assaeed , et al. 2024. “Genetic Variation Among *Aeluropus lagopoides* Populations Growing in Different Saline Regions.” Diversity 16, no. 1: 59. 10.3390/d16010059.

[eva70281-bib-0016] DeWoody, J. A. , A. M. Harder , S. Mathur , and J. R. Willoughby . 2021. “The Long‐Standing Significance of Genetic Diversity in Conservation.” Molecular Ecology 30, no. 17: 4147–4154. 10.1111/mec.16051. 34191374

[eva70281-bib-0017] Dobrowski, S. Z. 2011. “A Climatic Basis for Microrefugia: The Influence of Terrain on Climate.” Global Change Biology 17, no. 2: 1022–1035. 10.1111/j.1365-2486.2010.02263.

[eva70281-bib-0018] Dormann, C. F. , J. Elith , S. Bacher , et al. 2013. “Collinearity: A Review of Methods to Deal With It and A Simulation Study Evaluating Their Performance.” Ecography 36, no. 1: 27–46. 10.1111/j.1600-0587.2012.07348.x.

[eva70281-bib-0019] Earl, D. A. , and B. M. VonHoldt . 2012. “STRUCTURE HARVESTER: A Website and Program for Visualizing STRUCTURE Output and Implementing the Evanno Method.” Conservation Genetics Resources 4: 359–361. 10.1007/s12686-011-9548-7.

[eva70281-bib-0020] Escobar, S. , Y. Vigouroux , J. Karubian , L. Zekraoui , H. Balslev , and R. Montufar . 2023. “Limited Seed Dispersal Shapes Fine‐Scale Spatial Genetic Structure in A Neotropical Dioecious Large‐Seeded Palm.” Biotropica 55, no. 1: 160–172. 10.1111/btp.13172.

[eva70281-bib-0021] Foll, M. , and O. Gaggiotti . 2008. “A Genome‐Scan Method to Identify Selected Loci Appropriate for Both Dominant and Codominant Markers: A Bayesian Perspective.” Genetics 180, no. 2: 977–993. 10.1534/genetics.108.092221. 18780740 PMC2567396

[eva70281-bib-0022] Frei, D. , S. Mwaiko , O. Seehausen , and P. G. D. Feulner . 2023. “Ecological Disturbance Reduces Genomic Diversity Across an Alpine Whitefish Adaptive Radiation.” Evolutionary Applications 17, no. 2: e13617. 10.1111/eva.13617. 38343775 PMC10853656

[eva70281-bib-0023] Frichot, E. , and O. Francois . 2015. “LEA: An R Package for Landscape and Ecological Association Studies.” Methods in Ecology and Evolution 6, no. 8: 925–929. 10.1111/2041-210X.12382.

[eva70281-bib-0024] Fu, X. X. , H. N. Liu , X. D. Zhou , et al. 2013. “Seed Dormancy Mechanism and Dormancy Breaking Techniques for *Cornus kousa* Var. *Chinensis* .” Seed Science and Technology 41, no. 3: 458–463. 10.15258/sst.2013.41.3.12.

[eva70281-bib-0025] Gaitan‐Espitia, J. D. , and A. J. Hobday . 2020. “Evolutionary Principles and Genetic Considerations for Guiding Conservation Interventions Under Climate Change.” Global Change Biology 27, no. 3: 475–488. 10.1111/gcb.15359. 32979891

[eva70281-bib-0026] Garcia‐Costoya, G. , C. E. Williams , T. M. Faske , J. D. Moorman , and M. L. Logan . 2023. “Evolutionary Constraints Mediate Extinction Risk Under Climate Change.” Ecology Letters 26, no. 4: 529–539. 10.1111/ele.14173. 36756845

[eva70281-bib-0027] Giraldo, A. M. 2020. “Fenologia, Crecimiento Y Diversidad Genetica De Mimosa Trianae Benth En El Piedemonte Orinocense Y El Valle Del Cauca, Colombia.” https://repositorio.unal.edu.co/handle/unal/80001.

[eva70281-bib-0028] Guan, B. , J. Gao , W. Chen , X. Gong , and G. Ge . 2021. “The Effects of Climate Change on Landscape Connectivity and Genetic Clusters in A Small Subtropical and Warm‐Temperate Tree.” Frontiers in Plant Science 12: 671336. 10.3389/fpls.2021.671336. 34858443 PMC8631755

[eva70281-bib-0029] He, Z. X. , M. H. Wang , X. Y. Xu , Y. F. You , Y. Chen , and L. B. Zhang . 2021. “The Development of EST‐SSR Markers From Transcriptome Sequencing and Genetic Diversity of Twenty Genotypes With High Yields of *Cornus wilsoniana*, an Important Wood Oil Plant.” Molecular Plant Breeding 12, no. 5: 1–10. 10.5376/mpb.2021.12.0005.

[eva70281-bib-0030] Heller, N. E. , and E. S. Zavaleta . 2009. “Biodiversity Management in the Face of Climate Change: A Review of 22 Years of Recommendations.” Biological Conservation 142, no. 1: 14–32. 10.1016/j.biocon.2008.10.006.

[eva70281-bib-0031] Hoffmann, A. A. , V. L. White , M. Jasper , H. Yagui , S. J. Sinclair , and M. R. Kearney . 2021. “An Endangered Flightless Grasshopper With Strong Genetic Structure Maintains Population Genetic Variation Despite Extensive Habitat Loss.” Ecology and Evolution 11, no. 10: 5364–5380. 10.1002/ece3.7428. 34026013 PMC8131777

[eva70281-bib-0032] Hu, Z. M. , Q. S. Zhang , J. Zhang , et al. 2021. “Intraspecific Genetic Variation Matters When Predicting Seagrass Distribution Under Climate Change.” Molecular Ecology 30, no. 15: 3840–3855. 10.1111/mec.15996. 34022079

[eva70281-bib-0033] Jakobsson, M. , and N. A. Rosenberg . 2007. “CLUMPP: A Cluster Matching and Permutation Program for Dealing With Label Switching and Multimodality in Analysis of Population Structure.” Bioinformatics 23, no. 14: 1801–1806. 10.1093/bioinformatics/btm233. 17485429

[eva70281-bib-0034] Jay, F. , O. Francois , E. Y. Durand , and M. G. B. Blum . 2015. “POPs: A Software for Prediction of Population Genetic Structure Using Latent Regression Models.” Journal of Statistical Software 68, no. 9: 1–19. 10.18637/jss.v068.i09.

[eva70281-bib-0035] Jia, K. H. , W. Zhao , P. A. Maier , et al. 2019. “Landscape Genomics Predicts Climate Change‐Related Genetic Offset for the Widespread *Platycladus orientalis* (Cupressaceae).” Evolutionary Applications 13, no. 4: 665–676. 10.1111/eva.12891. 32211059 PMC7086053

[eva70281-bib-0036] Korte, A. , and A. Farlow . 2013. “The Advantages and Limitations of Trait Analysis With GWAS: A Review.” Plant Methods 9: 29. 10.1186/1746-4811-9-29. 23876160 PMC3750305

[eva70281-bib-0037] Lecocq, T. , H. Alexander , R. Pierre , and S. Oliver . 2019. “Integrating Intraspecific Differentiation in Species Distribution Models: Consequences on Projections of Current and Future Climatically Suitable Areas of Species.” Diversity and Distributions 25, no. 7: 1088–1100. 10.1111/ddi.12916.

[eva70281-bib-0038] Li, G. S. , L. J. Zhang , and C. K. Bai . 2012. “Chinese *Cornus officinalis* : Genetic Resources, Genetic Diversity and Core Collection.” Genetic Resources and Crop Evolution 59: 1659–1671. 10.1007/s10722-011-9789-z.

[eva70281-bib-0039] Li, H. , B. Handsaker , A. Wysoker , et al. 2009. “The Sequence Alignment/Map Format and SAMtools.” Bioinformatics 25, no. 16: 2078–2079. 10.1093/bioinformatics/btp352. 19505943 PMC2723002

[eva70281-bib-0040] Li, W. , and A. Godzik . 2006. “Cd‐Hit: A Fast Program for Clustering and Comparing Large Sets of Protein or Nucleotide Sequences.” Bioinformatics 22, no. 13: 1658–1659. 10.1093/bioinformatics/btl158. 16731699

[eva70281-bib-0041] Li, Y. , K. Cao , N. Li , et al. 2021. “Genomic Analyses Provide Insights Into Peach Local Adaptation and Responses to Climate Change.” Genome Research 31, no. 4: 592–606. 10.1101/gr.261032.120. 33687945 PMC8015852

[eva70281-bib-0042] Lima, J. S. , L. Ballesteros‐Mejia , M. S. Lima‐Ribeiro , and R. G. Collevatti . 2017. “Climatic Changes Can Drive the Loss of Genetic Diversity in A Neotropical Savanna Tree Species.” Global Change Biology 23, no. 11: 4639–4650. 10.1111/gcb.13685. 28295840

[eva70281-bib-0043] Lloyd, M. W. , H. R. Tumas , and M. C. Neel . 2018. “Limited Pollen Dispersal, Small Genetic Neighborhoods, and Biparental Inbreeding in *Vallisneria americana* .” American Journal of Botany 105, no. 2: 227–240. 10.1002/ajb2.1031. 29578290

[eva70281-bib-0044] Luo, S. J. , Y. H. He , G. G. Ning , J. Q. Zhang , G. Y. Ma , and M. Z. Bao . 2011. “Genetic Diversity and Genetic Structure of Different Populations of the Endangered Species *Davidia involucrata* in China Detected by Inter‐Simple Sequence Repeat Analysis.” Trees 25: 1063–1071. 10.1007/s00468-011-0581-7.

[eva70281-bib-0045] Malanoski, C. M. , A. Farnsworth , D. J. Lunt , P. J. Valdes , and E. E. Saupe . 2024. “Climate Change Is an Important Predictor of Extinction Risk on Macroevolutionary Timescales.” Science 383, no. 6687: 1130–1134. 10.1126/science.adj5763. 38452067

[eva70281-bib-0046] Meester, L. , R. Stoks , and K. I. Brans . 2018. “Genetic Adaptation as a Biological Buffer Against Climate Change: Potential and Limitations.” Integrative Zoology 13, no. 4: 372–391. 10.1111/1749-4877.12298. 29168625 PMC6221008

[eva70281-bib-0047] Nordstrom, S. W. , R. A. Hufbauer , L. Olazcuaga , L. F. Durkee , and B. A. Melbourne . 2023. “How Density Dependence, Genetic Erosion and the Extinction Vortex Impact Evolutionary Rescue.” Proceedings. Biological Sciences 290, no. 2011: 20231228. 10.1098/rspb.2023.1228. 37989246 PMC10688442

[eva70281-bib-0048] Peakall, R. , and P. E. Smouse . 2006. “GenAlEx 6: Genetic Analysis in Excel. Population Genetic Software for Teaching and Research.” Molecular Ecology Notes 6, no. 1: 288–295. 10.1111/j.1471-8286.2005.01155.x. PMC3463245 22820204

[eva70281-bib-0049] Pritchard, J. K. , M. Stephens , and P. Donnelly . 2000. “Inference of Population Structure Using Multilocus Genotype Data.” Genetics 155, no. 2: 945–959. 10.1093/genetics/155.2.945. 10835412 PMC1461096

[eva70281-bib-0050] Razgour, O. , B. Forester , J. B. Taggart , et al. 2019. “Considering Adaptive Genetic Variation in Climate Change Vulnerability Assessment Reduces Species Range Loss Projections.” Proceedings of the National Academy of Sciences of the United States of America 116, no. 21: 10418–10423. 10.1073/pnas.1820663116. 31061126 PMC6535011

[eva70281-bib-0051] Rico, Y. 2019. Landscape Genetics of Mexican Biodiversity: A Review. Vol. 29. Acta Universitaria. 10.15174/au.2019.1894.

[eva70281-bib-0052] Rivest, S. , G. Lajoie , D. A. Watts , and M. Vellend . 2021. “Earlier Spring Reduces Potential for Gene Flow via Reduced Flowering Synchrony Across an Elevational Gradient.” American Journal of Botany 108, no. 3: 538–545. 10.1002/ajb2.1627. 33733494

[eva70281-bib-0053] Roschanski, A. M. , K. Csillery , S. Liepelt , et al. 2016. “Evidence of Divergent Selection for Drought and Cold Tolerance at Landscape and Local Scales in *Abies blba* Mill. in the French Mediterranean Alps.” Molecular Ecology 25, no. 3: 776–794. 10.1111/mec.13516. 26676992

[eva70281-bib-0054] Rosenberg, N. A. 2003. “Distruct: A Program for the Graphical Display of Population Structure.” Molecular Ecology Notes 4, no. 1: 137–138. 10.1046/j.1471-8286.2003.00566.x.

[eva70281-bib-0055] Sampedro, L. , and R. Alía . 2023. “A Claim for a ‘Next Generation’ of Multisite Range‐Wide Forest Genetic Trials Built on the Legacy of Ecological Genetics to Anticipate Responses to Climate.” Global Change Biology 29, no. 17: 4700–4702. 10.1111/gcb.16816. 37317039

[eva70281-bib-0056] Schlaepfer, M. A. , and J. J. Lawler . 2022. “Conserving Biodiversity in the Face of Rapid Climate Change Requires a Shift in Priorities.” Wiley Interdisciplinary Reviews: Climate Change 14, no. 1: e798. 10.1002/wcc.798.

[eva70281-bib-0057] Shackleton, C. , T. Ticktin , and A. B. Cunningham . 2018. “Nontimber Forest Products as Ecological and Biocultural Keystone Species.” Ecology and Society 23, no. 4: 22. 10.5751/ES-10469-230422.

[eva70281-bib-0058] Shryock, D. F. , C. A. Havrilla , L. A. DeFalco , T. C. Esque , N. A. Custer , and T. E. Wood . 2016. “Landscape Genetic Approaches to Guide Native Plant Restoration in the Mojave Desert.” Ecological Applications 27, no. 2: 429–445. 10.1002/eap.1447. 28135767

[eva70281-bib-0059] Skogen, K. A. , R. P. Overson , E. T. Hilpman , and J. B. Fant . 2019. “Hawkmoth Pollination Facilitates Long‐Distance Pollen Dispersal and Reduces Isolation Across a Gradient of Land‐Use Change.” Annals of the Missouri Botanical Garden 104, no. 3: 495–511. 10.3417/2019475.

[eva70281-bib-0060] Spiegelhalter, D. J. , N. G. Best , B. P. Carlin , and A. V. Der Linde . 2002. “Bayesian Measures of Model Complexity and Fit.” Journal of the Royal Statistical Society, Series B: Statistical Methodology 64, no. 4: 583–639. 10.1111/1467-9868.00353.

[eva70281-bib-0061] Thuiller, W. , B. Lafourcade , R. Engler , et al. 2009. “BIOMOD—A Platform for Ensemble Forecasting of Species Distributions.” Ecography 32, no. 3: 369–373. 10.1111/j.1600-0587.2008.05742.

[eva70281-bib-0062] Vallejo‐Marin, M. , M. E. Dorken , and S. C. H. Barrett . 2010. “The Ecological and Evolutionary Consequences of Clonality for Plant Mating.” Annual Review of Ecology, Evolution, and Systematics 41: 193–213. 10.1146/annurev.ecolsys.110308.120258.

[eva70281-bib-0063] Verma, D. A. K. 2022. “Anthropogenic Activities and Biodiversity Threats.” International Journal of Biological Innovations 4, no. 1: 94–103. 10.46505/IJBI.2022.4110.

[eva70281-bib-0064] Walck, J. L. , S. N. Hidayati , K. W. Dixon , et al. 2011. “Climate Change and Plant Regeneration From Seed.” Global Change Biology 17, no. 6: 2145–2161. 10.1111/j.1365-2486.2010.02368.

[eva70281-bib-0065] Wang, T. , Z. Wang , F. Xia , and Y. Su . 2016. “Local Adaptation to Temperature and Precipitation in Naturally Fragmented Populations of *Cephalotaxus oliveri*, an Endangered Conifer Endemic to China.” Scientific Reports 6: 25031. 10.1038/srep25031. 27113970 PMC4844950

[eva70281-bib-0066] Wang, T. R. , H. H. Meng , N. Wang , et al. 2023. “Adaptive Divergence and Genetic Vulnerability of Relict Species Under Climate Change: A Case Study of *Pterocarya macroptera* .” Annals of Botany 132, no. 2: 241–254. 10.1093/aob/mcad083. 37409981 PMC10583204

[eva70281-bib-0067] Wu, L. W. , Y. Zhang , X. Guo , et al. 2022. “Reduction of Microbial Diversity in Grassland Soil Is Driven by Long‐Term Climate Warming.” Nature Microbiology 7, no. 7: 1054–1062. 10.1038/s41564-022-01147-3. 35697795

[eva70281-bib-0068] Wudu, K. , A. Abegaz , L. Ayele , and Y. Mussie . 2023. “The Impacts of Climate Change on Biodiversity Loss and Its Remedial Measures Using Nature Based Conservation Approach: A Global Perspective.” Biodiversity and Conservation 32: 3681–3701. 10.1007/s10531-023-02656-1.

[eva70281-bib-0069] Xiang, Q. Y. , and D. E. Boufford . 2005. “Cornaceae, Mastixiaceae, Toricelliaceae, Helwingiaceae, Aucubaceae.” In Flora of China, edited by Z. Y. Wu and P. H. Raven , vol. 14. Science Press. 206–234.

[eva70281-bib-0070] Yuan, J. , Q. Fang , G. Liu , and X. Fu . 2019. “Low Divergence Among Natural Populations of *Cornus kousa* Subsp *chinensis* Revealed by ISSR Markers.” Forests 10: 1082. 10.3390/f10121082.

[eva70281-bib-0071] Zu, K. , F. Chen , Y. Li , et al. 2023. “Climate Change Impacts Flowering Phenology in Gongga Mountains, Southwest China.” Plant Diversity 46, no. 6: 774–782. 10.1016/j.pld.2023.07.007. 39811806 PMC11725964

