# Supplementary material for: Climate‐Induced Genomic Selection: Genetic Structure Variation of Cornus kousa subsp. chinensis and Its Adaptive Reaction to Future Climate Change
Source: Evol Appl. 2026 Jun 18;19(6):e70281. doi: 10.1111/eva.70281 (PMC13276676; doi:10.1111/eva.70281)
Supplement: Supplementary file 1 — Figure S1: The distribution changes of C. kousa under two future climate scenarios simulated by SDM Toolbox. Table S1: The geographical locations and number of samples of Cornus kousa populations in the study. Table S2: Summary table of 20 environmental factors used in the study. Table S3: Genotyping‐by‐sequencing (GBS) outcomes of 348 samples of Cornus kousa sourced from 25 natural populations. Table S4: A statistical tabulation presenting the results of enzymatic digestion for 348 samples of Cornus kousa procured from 25 natural populations. Table S5: A statistical table presenting the tag fragments of 348 samples of Cornus kousa sourced from 25 natural populations. Table S6: A statistical tabulation detailing the comparison between the reads and the reference genome of 348 samples of Cornus kousa sourced from 25 natural populations. Table S7: Evaluation indices (AUC, Kappa, TSS) for the potential distribution areas of Cornus kousa under current and future (RCP45, RCP85, 2070) climate scenarios, based on the ensemble model. Table S8: The Deviance Information Criterion (DIC) value and the correlation associated with the simulation of POPs. [file EVA-19-e70281-s001.docx]

**Supporting Information:**

Additional supporting information can be found online in the Supporting Information section. **FIGURE S1:** Information on the distribution changes of *C. kousa* under two future climate scenarios. **TABLE S1**: Information on sampling locations and population sizes. **TABLE S2**: Information on environmental factors. **TABLE S3**: Information on Genotyping-by-Sequencing outcomes. **TABLE S4**: Information on enzymatic digestion results. **TABLE S5**: Information on tag fragment statistics. **TABLE S6**: Information on read mapping to reference genome. **TABLE S7**: Evaluation indices (AUC, Kappa, TSS) of the ensemble model for potential distribution areas under current and future climates. **TABLE S8**: Deviance Information Criterion (DIC) values and correlations from POPs simulations. **TABLE S9**: List of 72 outlier loci detected by BayeScan.

**
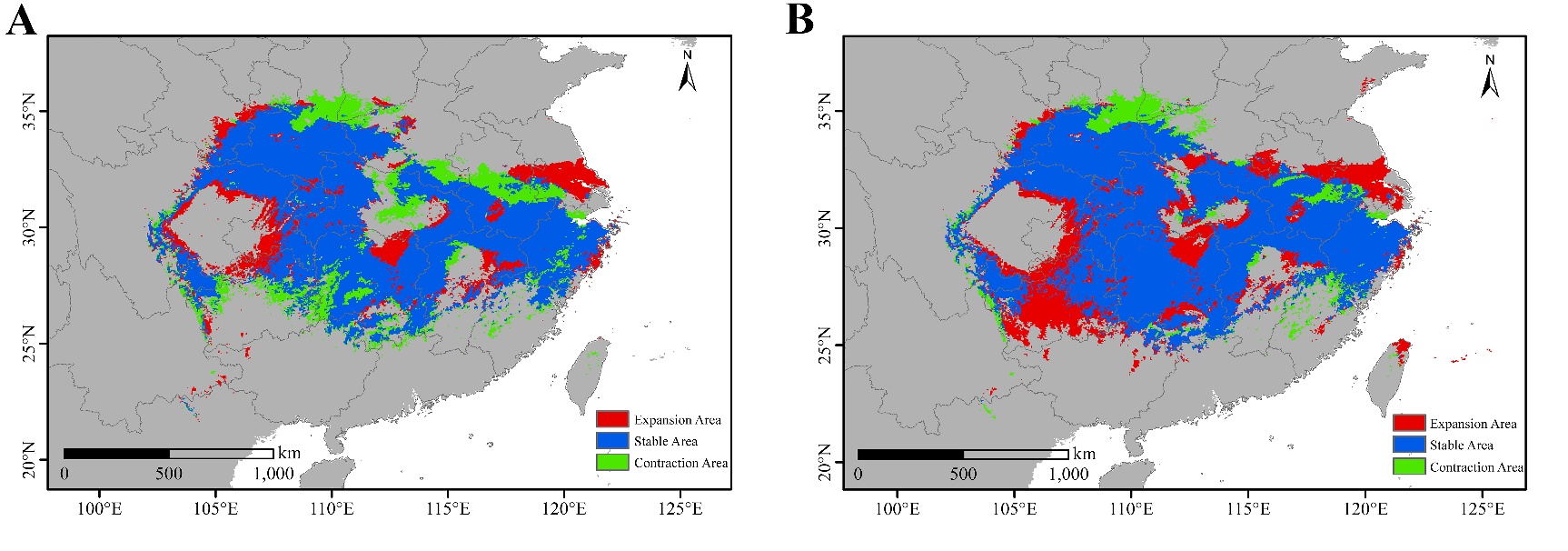
**

**FIGURE S1.** The distribution changes of *C. kousa* under two future climate scenarios simulated by SDM Toolbox: (A) RCP45, (B) RCP85.

**TABLE S1.** The geographical locations and number of samples of *C. kousa* populations in the study.

| Population numbers | Population code | Location | Area | Longitude  (°E) | Latitude  (°N) | Sample numbers | Altitude  (m) | |
| --- | --- | --- | --- | --- | --- | --- | --- | --- |
| 1 | HG | Lu Shan | Jiujiang City, Jiangxi Province | 115.99 | 29.57 | 36 | 1099 | |
| 2 | FN | Fu Niu Shan | Nanyang City, Henan Province | 112.03 | 33.55 | 26 | 1001 | |
| 3 | TZ | Tian Tang Zhai | Liuan City, Anhui Province | 115.78 | 31.14 | 20 | 1073 | |
| 4 | ES | Tian Sang Ping | Enshi Prefecture, Hubei Province | 110.27 | 30.84 | 20 | 437 | |
| 5 | SN | Shen Nong Jia | Shennongjia Forest District, Hubei Province | 110.68 | 31.75 | 14 | 945 | |
| 6 | ST | Tai Bai Shan | Baoji City, Shaanxi Province | 107.71 | 34.09 | 21 | 1102.2 | |
| 7 | LH | Lao Hu Nao | Fuzhou City, Jiangxi Province | 116.02 | 27.20 | 16 | 1220 | |
| 8 | TB | Tong Bai Shan | Suizhou City, Hubei Province | 113.27 | 32.38 | 20 | 986 | |
| 9 | HS | Heng Shan | Hengyang City, Hunan Province | 112.68 | 27.28 | 19 | 1201 | |
| 10 | HA | Tian Tai Shan | Huanggang City, Hubei Province | 114.64 | 31.58 | 20 | 741 | |
| 11 | JG | Ji Gong Shan | Xinyang City, Henan Province | 114.08 | 31.81 | 16 | 707 | |
| 12 | JLS | Jiu Ling Shan | Yichun City, Jiangxi Province | 114.91 | 28.86 | 19 | 1714 | |
| 13 | JHS | Jiu Hua Shan | Chizhou City, Anhui Province | 117.83 | 30.48 | 16 | 1215.2 | |
| 14 | SC | Rong Mu Ba | Guangyuan City, Sichuan Province | 106.60 | 32.35 | 20 | 1253 |  |
| 15 | AK | Huang An Ba | Ankang City, Shaanxi Province | 109.29 | 31.85 | 20 | 1298.7 |  |
| 16 | TMS | Tian Men Shan | Zhangjiajie City, Hunan Province | 110.48 | 29.05 | 20 | 1443 |  |
| 17 | AHS | Huang Shan | Huangshan City, Anhui Province | 118.17 | 30.13 | 20 | 1787 |  |
| 18 | XE | He Long Shan | Enshi Prefecture, Hubei Province | 109.71 | 30.03 | 20 | 1153 |  |
| 19 | SZ | Huang Shui | Shizhu County, Chongqing City | 108.35 | 30.23 | 20 | 1510 |  |
| 20 | MS | Tian Mu Shan | Hangzhou City, Zhejiang Province | 119.43 | 30.35 | 15 | 1383 |  |
| 21 | JZJ | Jin Zi Jian | Hangzhou City, Zhejiang Province | 118.96 | 29.8 | 5 | 1448 |  |
| 22 | LN | Long Nan Forest Farm | Longnan City, Gansu Province | 105.82 | 33.24 | 21 | 1624.97 |  |
| 23 | LS | Li Shan | Jincheng City, Shanxi Province | 111.96 | 35.39 | 4 | 1615 |  |
| 24 | BHS | Bao Hua Shan | Zhenjiang City, Jiangsu Province | 119.1 | 32.13 | 15 | 309 |  |
| 25 | MX | Xin Dian Zi Forest Farm | Hanzhong City, Shaanxi Province | 106.76 | 33.32 | 15 | 1278.31 |  |

**TABLE S2.** Summary table of twenty environmental factors used in the study.

| **Variable** | **Description** |
| --- | --- |
| Bio 1 | Annual mean temperature |
| Bio 2 | Mean diurnal Range |
| Bio 3 | Isothermality |
| Bio 4 | Temperature seasonality |
| Bio 5 | Max temperature of warmest month |
| Bio 6 | Min temperature of coldest month |
| Bio 7 | Temperature annual range |
| Bio 8 | Mean temperature of wettest quarter |
| Bio 9 | Mean temperature of driest quarter |
| Bio 10 | Mean temperature of warmest quarter |
| Bio 11 | Mean temperature of coldest quarter |
| Bio 12 | Annual precipitation |
| Bio 13 | Precipitation of wettest month |
| Bio 14 | Precipitation of driest month |
| Bio 15 | Precipitation seasonality (Coefficient of variation) |
| Bio 16 | Precipitation of wettest quarter |
| Bio 17 | Precipitation of driest quarter |
| Bio 18 | Precipitation of warmest quarter |
| Bio 19 | Precipitation of coldest quarter |
| Ele | Elevation |

**TABLE S3.** Genotyping-by-Sequencing (GBS) outcomes of 348 samples of *C. kousa* sourced from 25 natural populations*.*

| Sample | Raw base (bp) | Clean base (bp) | Effective rate (%) | Error rate (%) | Q20 (%) | Q30 (%) | GC content (%) |
| --- | --- | --- | --- | --- | --- | --- | --- |
| AHS1 | 296,179,488 | 278,864,928 | 94.15 | 0.03 | 97.22 | 92.31 | 39.34 |
| AHS10 | 625,100,256 | 558,301,824 | 89.31 | 0.03 | 96.98 | 91.75 | 38.89 |
| AHS11 | 386,659,296 | 377,075,232 | 97.52 | 0.03 | 96.09 | 89.77 | 38.48 |
| AHS12 | 478,921,536 | 462,653,856 | 96.6 | 0.03 | 96.46 | 90.66 | 38.53 |
| AHS13 | 376,556,832 | 364,595,904 | 96.82 | 0.04 | 94.25 | 85.36 | 38.9 |
| AHS14 | 398,889,504 | 377,957,376 | 94.75 | 0.04 | 94.25 | 85.38 | 39.02 |
| AHS15 | 366,209,568 | 353,494,080 | 96.53 | 0.03 | 95.5 | 88.02 | 39.27 |
| AHS2 | 481,447,584 | 461,409,408 | 95.84 | 0.03 | 96.31 | 90.12 | 38.91 |
| AHS3 | 337,834,944 | 323,432,064 | 95.74 | 0.03 | 96.69 | 91.06 | 39.17 |
| AHS4 | 517,317,408 | 498,342,816 | 96.33 | 0.03 | 96.06 | 89.55 | 39.34 |
| AHS5 | 282,147,552 | 261,346,176 | 92.63 | 0.03 | 96.88 | 91.6 | 39.32 |
| AHS6 | 319,010,688 | 294,047,712 | 92.17 | 0.03 | 96.73 | 91.24 | 39.19 |
| AHS7 | 345,417,984 | 328,379,616 | 95.07 | 0.03 | 96.45 | 90.43 | 38.67 |
| AHS8 | 694,328,544 | 649,845,792 | 93.59 | 0.04 | 95.16 | 87.54 | 39.13 |
| AHS9 | 483,249,600 | 473,607,936 | 98 | 0.04 | 94.45 | 85.83 | 38.47 |
| AK1 | 448,549,632 | 430,699,680 | 96.02 | 0.03 | 96 | 89.48 | 39.22 |
| AK10 | 293,910,912 | 280,410,048 | 95.41 | 0.03 | 96.3 | 90.13 | 39.39 |
| AK11 | 267,747,840 | 259,672,320 | 96.98 | 0.03 | 96.78 | 91.27 | 38.95 |
| AK12 | 435,587,616 | 423,175,968 | 97.15 | 0.03 | 96.25 | 90.07 | 38.49 |
| AK13 | 278,812,512 | 271,987,488 | 97.55 | 0.03 | 96.72 | 91.09 | 39.22 |
| AK14 | 282,166,272 | 266,555,232 | 94.47 | 0.03 | 96.56 | 90.72 | 39.53 |
| AK15 | 348,448,032 | 326,369,664 | 93.66 | 0.03 | 95.95 | 89.22 | 39.23 |
| AK2 | 296,895,456 | 280,361,088 | 94.43 | 0.03 | 96.48 | 90.6 | 38.84 |
| AK3 | 336,842,784 | 302,937,120 | 89.93 | 0.03 | 96.02 | 89.37 | 38.59 |
| AK4 | 336,705,696 | 331,011,936 | 98.31 | 0.04 | 94.22 | 85.28 | 38.3 |
| AK5 | 277,094,592 | 235,987,200 | 85.16 | 0.03 | 96.59 | 90.97 | 38.82 |
| AK6 | 559,387,872 | 528,920,352 | 94.55 | 0.03 | 95.78 | 88.94 | 39.59 |
| AK7 | 308,258,496 | 289,982,016 | 94.07 | 0.03 | 96.69 | 91.11 | 39.13 |
| AK8 | 255,988,224 | 250,451,136 | 97.84 | 0.03 | 96.46 | 90.56 | 38.7 |
| AK9 | 251,375,904 | 236,731,968 | 94.17 | 0.03 | 96.59 | 90.81 | 39.36 |
| BHS1 | 445,030,560 | 377,078,688 | 84.73 | 0.03 | 96.81 | 91.25 | 38.14 |
| BHS12 | 381,720,096 | 348,501,312 | 91.3 | 0.03 | 97.02 | 91.78 | 37.75 |
| BHS13 | 413,779,392 | 375,573,024 | 90.77 | 0.03 | 96.79 | 91.1 | 38.41 |
| BHS14 | 447,668,928 | 421,208,928 | 94.09 | 0.03 | 97.2 | 92.21 | 38.05 |
| BHS15 | 417,183,840 | 399,792,384 | 95.83 | 0.04 | 95.52 | 88.15 | 38.05 |
| BHS2 | 447,412,320 | 410,220,288 | 91.69 | 0.03 | 96.97 | 91.64 | 38.14 |
| BHS3 | 400,424,256 | 374,727,456 | 93.58 | 0.03 | 96.49 | 90.39 | 38.09 |
| BHS4 | 391,042,368 | 341,938,080 | 87.44 | 0.03 | 97.39 | 92.78 | 37.92 |
| BHS5 | 374,872,896 | 328,436,640 | 87.61 | 0.03 | 97.53 | 93.16 | 37.9 |
| BHS6 | 428,087,232 | 343,671,552 | 80.28 | 0.03 | 96.97 | 91.72 | 38.14 |
| BHS7 | 468,485,568 | 432,643,968 | 92.35 | 0.03 | 97.03 | 91.78 | 38.12 |
| ES1 | 449,726,400 | 398,630,304 | 88.64 | 0.03 | 95.95 | 89.48 | 38.97 |
| ES10 | 476,585,568 | 427,942,944 | 89.79 | 0.03 | 96.82 | 91.72 | 39.08 |
| ES11 | 201,379,104 | 178,468,992 | 88.62 | 0.03 | 96.48 | 90.89 | 38.77 |
| ES12 | 141,710,112 | 137,822,976 | 97.26 | 0.03 | 96.12 | 89.88 | 38.05 |
| ES13 | 193,349,952 | 182,589,696 | 94.43 | 0.03 | 95.96 | 89.45 | 38.2 |
| ES14 | 226,207,584 | 192,577,248 | 85.13 | 0.03 | 95.77 | 89.09 | 38.77 |
| ES15 | 243,147,456 | 212,044,032 | 87.21 | 0.03 | 95.8 | 89.1 | 38.66 |
| ES16 | 177,966,720 | 171,481,536 | 96.36 | 0.03 | 96.19 | 90.16 | 38.49 |
| ES2 | 478,456,416 | 408,369,024 | 85.35 | 0.03 | 95.88 | 89.34 | 39.3 |
| ES3 | 541,930,752 | 466,820,064 | 86.14 | 0.03 | 95.87 | 89.29 | 39.12 |
| ES4 | 493,234,560 | 433,836,288 | 87.96 | 0.03 | 96.34 | 90.52 | 39.08 |
| ES5 | 217,393,632 | 193,730,976 | 89.12 | 0.03 | 95.78 | 89 | 38.92 |
| ES7 | 186,588,576 | 170,613,792 | 91.44 | 0.03 | 97.04 | 92.05 | 39.05 |
| ES8 | 210,144,384 | 197,656,416 | 94.06 | 0.03 | 96.49 | 90.77 | 39.02 |
| ES9 | 196,473,600 | 179,464,032 | 91.34 | 0.03 | 96.71 | 91.41 | 38.94 |
| FN1 | 211,874,688 | 178,386,624 | 84.19 | 0.03 | 96.62 | 91.32 | 38.6 |
| FN10 | 297,466,848 | 243,712,800 | 81.93 | 0.03 | 96.41 | 90.38 | 38.16 |
| FN11 | 305,471,520 | 261,880,128 | 85.73 | 0.03 | 96.75 | 91.11 | 38.23 |
| FN12 | 343,912,608 | 296,859,168 | 86.32 | 0.03 | 95.31 | 87.77 | 38.16 |
| FN13 | 380,997,216 | 358,787,232 | 94.17 | 0.03 | 95.41 | 87.95 | 37.81 |
| FN14 | 324,766,656 | 267,704,352 | 82.43 | 0.04 | 94.23 | 85.57 | 38.12 |
| FN15 | 515,409,984 | 431,515,872 | 83.72 | 0.03 | 96.54 | 90.97 | 39.02 |
| FN2 | 203,095,008 | 171,035,136 | 84.21 | 0.03 | 96.32 | 90.5 | 38.52 |
| FN3 | 228,724,992 | 212,212,512 | 92.78 | 0.03 | 96.36 | 90.54 | 38.92 |
| FN4 | 195,179,040 | 176,002,848 | 90.18 | 0.03 | 96.95 | 91.97 | 38.95 |
| FN5 | 206,935,776 | 180,914,400 | 87.43 | 0.03 | 96.54 | 91 | 38.99 |
| FN6 | 239,792,256 | 211,052,448 | 88.01 | 0.03 | 95.46 | 88.32 | 38.7 |
| FN7 | 217,676,448 | 192,850,272 | 88.59 | 0.03 | 95.99 | 89.57 | 38.92 |
| FN8 | 217,578,528 | 202,590,144 | 93.11 | 0.03 | 96.19 | 90.17 | 39.02 |
| FN9 | 327,937,248 | 299,362,176 | 91.29 | 0.04 | 93.39 | 83.87 | 38.2 |
| HA1 | 647,061,696 | 579,048,768 | 89.49 | 0.03 | 96.04 | 89.54 | 39.61 |
| HA11 | 834,577,920 | 767,114,208 | 91.92 | 0.03 | 96.36 | 90.28 | 39.53 |
| HA12 | 606,392,064 | 574,281,792 | 94.7 | 0.03 | 96.92 | 91.53 | 37.95 |
| HA13 | 636,572,160 | 575,540,352 | 90.41 | 0.03 | 96.94 | 91.62 | 38.56 |
| HA14 | 676,109,088 | 580,254,336 | 85.82 | 0.03 | 96.81 | 91.19 | 38.91 |
| HA15 | 511,743,744 | 487,341,504 | 95.23 | 0.03 | 96.57 | 90.97 | 39.08 |
| HA16 | 429,016,032 | 386,637,984 | 90.12 | 0.03 | 96.47 | 90.69 | 39.62 |
| HA2 | 641,644,704 | 548,964,288 | 85.56 | 0.03 | 96.25 | 90.07 | 39.26 |
| HA3 | 593,453,376 | 518,715,648 | 87.41 | 0.03 | 95.44 | 88.14 | 39.51 |
| HA4 | 823,222,656 | 745,392,672 | 90.55 | 0.04 | 94.65 | 86.47 | 39.22 |
| HA5 | 744,503,616 | 554,544,288 | 74.49 | 0.03 | 96.03 | 89.72 | 38.32 |
| HA6 | 808,018,272 | 731,280,672 | 90.5 | 0.03 | 95.33 | 87.88 | 39.47 |
| HA7 | 483,612,480 | 439,824,672 | 90.95 | 0.03 | 96.59 | 91.08 | 38.55 |
| HA8 | 712,320,768 | 677,458,080 | 95.11 | 0.04 | 92.91 | 82.82 | 39.49 |
| HA9 | 696,363,264 | 620,746,848 | 89.14 | 0.03 | 96.24 | 90.08 | 38.79 |
| HG1 | 425,612,160 | 390,017,664 | 91.64 | 0.03 | 97.22 | 92.22 | 37.84 |
| HG10 | 768,970,368 | 648,652,320 | 84.35 | 0.03 | 95.56 | 88.53 | 39.33 |
| HG11 | 754,426,656 | 636,945,696 | 84.43 | 0.03 | 95.97 | 89.5 | 39.13 |
| HG12 | 719,803,008 | 660,040,704 | 91.7 | 0.04 | 94.8 | 86.77 | 39.68 |
| HG13 | 597,849,984 | 539,473,824 | 90.24 | 0.03 | 96.89 | 91.6 | 39.22 |
| HG14 | 440,615,808 | 378,597,888 | 85.92 | 0.03 | 96.37 | 90.58 | 38.88 |
| HG15 | 510,276,960 | 463,701,024 | 90.87 | 0.03 | 97.19 | 92.27 | 39.33 |
| HG16 | 475,640,928 | 423,033,984 | 88.94 | 0.03 | 96.16 | 90.06 | 39.39 |
| HG2 | 468,509,184 | 421,317,792 | 89.93 | 0.03 | 97.16 | 92.11 | 37.94 |
| HG3 | 439,527,168 | 415,697,184 | 94.58 | 0.03 | 97.17 | 92.1 | 38.26 |
| HG4 | 383,020,128 | 338,355,648 | 88.34 | 0.03 | 97.12 | 91.96 | 38.28 |
| HG5 | 450,900,576 | 382,229,280 | 84.77 | 0.03 | 96.75 | 90.99 | 38.12 |
| HG6 | 505,442,304 | 418,271,328 | 82.75 | 0.03 | 97.53 | 93.06 | 37.97 |
| HG7 | 461,835,360 | 430,988,832 | 93.32 | 0.03 | 96.28 | 90.41 | 38.34 |
| HG9 | 705,050,208 | 645,135,840 | 91.5 | 0.04 | 94.96 | 87.13 | 39.3 |
| HS1 | 508,893,120 | 500,707,296 | 98.39 | 0.03 | 96.08 | 89.54 | 37.55 |
| HS11 | 432,432,576 | 379,981,440 | 87.87 | 0.03 | 95.95 | 89.3 | 37.95 |
| HS12 | 485,135,136 | 470,509,344 | 96.99 | 0.03 | 96.41 | 90.36 | 37.39 |
| HS13 | 467,103,456 | 441,472,320 | 94.51 | 0.03 | 96.41 | 90.28 | 37.66 |
| HS14 | 427,562,496 | 387,396,288 | 90.61 | 0.03 | 96.18 | 89.81 | 38.09 |
| HS15 | 480,371,616 | 443,548,800 | 92.33 | 0.04 | 95.48 | 88.07 | 38.02 |
| HS16 | 464,081,472 | 428,822,496 | 92.4 | 0.03 | 97.09 | 91.92 | 38.02 |
| HS17 | 475,396,128 | 449,008,416 | 94.45 | 0.03 | 96.4 | 90.3 | 37.88 |
| HS2 | 451,439,424 | 428,104,512 | 94.83 | 0.04 | 95.54 | 88.16 | 37.99 |
| HS3 | 450,805,824 | 439,405,344 | 97.47 | 0.04 | 93.23 | 83.19 | 38.1 |
| HS4 | 486,596,736 | 461,687,328 | 94.88 | 0.03 | 96.44 | 90.37 | 38.05 |
| HS5 | 484,123,104 | 462,478,752 | 95.53 | 0.03 | 96.43 | 90.39 | 38.02 |
| HS6 | 461,736,288 | 450,494,784 | 97.57 | 0.03 | 96.12 | 89.73 | 37.1 |
| HS7 | 512,233,920 | 445,612,320 | 86.99 | 0.03 | 96.32 | 90.46 | 39.03 |
| HS8 | 415,481,472 | 382,059,936 | 91.96 | 0.03 | 96.14 | 89.78 | 37.93 |
| JG1 | 369,760,896 | 353,421,792 | 95.58 | 0.03 | 95.19 | 87.42 | 38.11 |
| JG11 | 597,370,752 | 505,311,552 | 84.59 | 0.03 | 95.96 | 89.64 | 39.16 |
| JG12 | 341,257,536 | 300,741,984 | 88.13 | 0.04 | 93.9 | 84.84 | 38.13 |
| JG13 | 370,629,504 | 334,704,672 | 90.31 | 0.04 | 93.97 | 84.9 | 37.95 |
| JG14 | 391,381,344 | 373,475,232 | 95.42 | 0.03 | 95.23 | 87.57 | 37.9 |
| JG15 | 396,364,320 | 375,193,440 | 94.66 | 0.03 | 96.1 | 89.56 | 37.94 |
| JG17 | 419,368,608 | 392,656,896 | 93.63 | 0.03 | 95.41 | 87.94 | 37.83 |
| JG18 | 291,358,656 | 247,080,384 | 84.8 | 0.03 | 95.62 | 88.52 | 37.75 |
| JG2 | 339,035,904 | 324,079,776 | 95.59 | 0.04 | 93.84 | 84.59 | 38.08 |
| JG3 | 323,567,424 | 298,695,456 | 92.31 | 0.03 | 96.6 | 90.57 | 38.3 |
| JG5 | 385,105,248 | 374,350,176 | 97.21 | 0.03 | 95.55 | 88.19 | 37.97 |
| JG6 | 365,120,064 | 346,667,616 | 94.95 | 0.03 | 96.2 | 89.69 | 38.47 |
| JG7 | 365,395,392 | 346,967,136 | 94.96 | 0.03 | 95.72 | 88.59 | 38.02 |
| JG8 | 305,196,480 | 299,068,416 | 97.99 | 0.03 | 94.88 | 86.78 | 37.11 |
| JG9 | 301,512,384 | 265,517,856 | 88.06 | 0.04 | 94.5 | 86.09 | 37.77 |
| JHS1 | 418,030,272 | 401,224,608 | 95.98 | 0.03 | 96.59 | 90.62 | 38.21 |
| JHS10 | 409,283,424 | 378,784,512 | 92.55 | 0.03 | 96.91 | 91.6 | 38.11 |
| JHS11 | 346,791,456 | 300,120,480 | 86.54 | 0.03 | 97.18 | 92.27 | 38.08 |
| JHS12 | 434,091,456 | 388,061,856 | 89.4 | 0.03 | 96.21 | 89.91 | 38.06 |
| JHS13 | 455,618,592 | 420,416,928 | 92.27 | 0.03 | 96.32 | 90.16 | 37.71 |
| JHS14 | 398,908,224 | 342,008,640 | 85.74 | 0.03 | 95.55 | 88.33 | 37.87 |
| JHS15 | 454,941,792 | 439,030,656 | 96.5 | 0.04 | 94.95 | 86.86 | 37.89 |
| JHS2 | 600,511,392 | 508,804,992 | 84.73 | 0.03 | 96.2 | 90.31 | 38.79 |
| JHS3 | 363,002,976 | 333,657,792 | 91.92 | 0.03 | 95.88 | 88.98 | 38.19 |
| JHS4 | 520,317,216 | 481,701,600 | 92.58 | 0.03 | 95.5 | 88.4 | 39.45 |
| JHS5 | 392,626,368 | 374,691,744 | 95.43 | 0.04 | 93.47 | 83.93 | 37.91 |
| JHS6 | 459,212,256 | 410,851,008 | 89.47 | 0.03 | 96.93 | 91.96 | 38.88 |
| JHS7 | 396,993,312 | 360,397,728 | 90.78 | 0.03 | 97.16 | 92.44 | 38.99 |
| JHS8 | 364,159,296 | 339,936,480 | 93.35 | 0.03 | 94.62 | 86.26 | 38.16 |
| JHS9 | 434,984,544 | 394,102,656 | 90.6 | 0.04 | 95.05 | 87.15 | 37.89 |
| JLS1 | 313,564,032 | 295,120,512 | 94.12 | 0.04 | 93.06 | 83.22 | 38.12 |
| JLS10 | 346,459,104 | 334,620,000 | 96.58 | 0.03 | 95.2 | 87.56 | 37.1 |
| JLS11 | 611,692,704 | 552,970,080 | 90.4 | 0.03 | 95.48 | 88.47 | 38.9 |
| JLS12 | 340,831,872 | 310,821,984 | 91.2 | 0.03 | 95.32 | 87.84 | 38.03 |
| JLS13 | 356,633,856 | 323,746,560 | 90.78 | 0.03 | 94.93 | 86.92 | 38.1 |
| JLS14 | 376,132,320 | 356,302,080 | 94.73 | 0.03 | 95.64 | 88.44 | 37.7 |
| JLS15 | 355,771,296 | 345,523,104 | 97.12 | 0.03 | 95.44 | 88 | 37.59 |
| JLS2 | 393,571,872 | 380,357,280 | 96.64 | 0.03 | 95.37 | 87.77 | 38.14 |
| JLS3 | 386,659,296 | 372,149,280 | 96.25 | 0.04 | 93.96 | 84.9 | 38.09 |
| JLS4 | 195,981,120 | 189,964,512 | 96.93 | 0.05 | 90.58 | 78.94 | 38.5 |
| JLS5 | 608,573,664 | 552,085,920 | 90.72 | 0.03 | 96.38 | 90.69 | 38.97 |
| JLS6 | 584,058,240 | 521,075,232 | 89.22 | 0.03 | 96.94 | 92.06 | 38.66 |
| JLS7 | 330,389,856 | 324,775,296 | 98.3 | 0.03 | 95.45 | 88.03 | 37.62 |
| JLS8 | 599,501,376 | 507,196,800 | 84.6 | 0.03 | 96.55 | 91.12 | 38.77 |
| JLS9 | 375,677,568 | 363,040,416 | 96.64 | 0.03 | 95.75 | 88.65 | 38.31 |
| JZJ1 | 487,170,432 | 470,162,592 | 96.51 | 0.04 | 93.16 | 84.35 | 37.47 |
| JZJ2 | 400,519,872 | 391,425,408 | 97.73 | 0.04 | 94.11 | 86.17 | 36.56 |
| JZJ3 | 526,887,072 | 507,187,008 | 96.26 | 0.04 | 94.15 | 86.14 | 37.08 |
| JZJ4 | 519,009,120 | 487,073,376 | 93.85 | 0.04 | 93.71 | 85.37 | 37.58 |
| JZJ5 | 559,256,256 | 527,938,272 | 94.4 | 0.04 | 92.36 | 82.81 | 37.77 |
| LH10 | 641,379,744 | 565,913,952 | 88.23 | 0.03 | 96.25 | 90.35 | 38.37 |
| LH11 | 627,640,128 | 581,244,192 | 92.61 | 0.03 | 95.81 | 89.14 | 38.95 |
| LH12 | 327,496,032 | 310,849,920 | 94.92 | 0.03 | 96.06 | 89.42 | 39.36 |
| LH13 | 503,562,528 | 473,786,208 | 94.09 | 0.04 | 94.03 | 85.16 | 39.2 |
| LH14 | 565,358,976 | 494,712,288 | 87.5 | 0.03 | 96.41 | 90.75 | 38.39 |
| LH15 | 269,134,560 | 256,468,320 | 95.29 | 0.03 | 96.51 | 90.64 | 39.45 |
| LH16 | 249,806,304 | 232,978,464 | 93.26 | 0.03 | 96.97 | 91.81 | 39.33 |
| LH2 | 469,477,728 | 428,238,432 | 91.22 | 0.03 | 96.91 | 91.48 | 38.38 |
| LH3 | 495,369,216 | 444,276,288 | 89.69 | 0.03 | 96.52 | 90.64 | 38.08 |
| LH4 | 450,552,096 | 432,923,328 | 96.09 | 0.03 | 95.97 | 89.28 | 37.42 |
| LH5 | 249,743,232 | 243,694,944 | 97.58 | 0.03 | 96.03 | 89.44 | 38.56 |
| LH6 | 327,087,360 | 302,613,984 | 92.52 | 0.03 | 96.08 | 89.52 | 39.41 |
| LH7 | 526,831,776 | 481,654,944 | 91.42 | 0.03 | 96.42 | 90.71 | 39 |
| LH8 | 502,061,184 | 438,607,872 | 87.36 | 0.03 | 95.92 | 89.41 | 39.28 |
| LH9 | 592,473,024 | 546,869,376 | 92.3 | 0.03 | 95.3 | 88.03 | 38.82 |
| LN1 | 529,732,800 | 491,032,800 | 92.69 | 0.03 | 96.91 | 91.35 | 38.08 |
| LN10 | 490,276,224 | 450,928,512 | 91.97 | 0.03 | 96.97 | 91.54 | 37.87 |
| LN11 | 412,424,640 | 338,430,816 | 82.06 | 0.03 | 97.26 | 92.28 | 38.03 |
| LN12 | 504,472,032 | 480,612,672 | 95.27 | 0.03 | 96.11 | 89.5 | 38.09 |
| LN13 | 569,530,656 | 531,914,688 | 93.4 | 0.03 | 96.33 | 89.98 | 38.04 |
| LN14 | 509,452,704 | 481,964,832 | 94.6 | 0.03 | 95.44 | 87.91 | 38.06 |
| LN15 | 505,423,872 | 485,177,184 | 95.99 | 0.04 | 94.69 | 86.31 | 37.65 |
| LN2 | 444,425,760 | 429,901,344 | 96.73 | 0.03 | 96.38 | 90.07 | 37.88 |
| LN3 | 554,639,328 | 527,628,672 | 95.13 | 0.03 | 96.28 | 89.93 | 37.59 |
| LN4 | 535,080,384 | 517,766,400 | 96.76 | 0.03 | 97.05 | 91.72 | 37.48 |
| LN5 | 498,347,136 | 474,380,064 | 95.19 | 0.03 | 96.55 | 90.53 | 37.73 |
| LN6 | 301,735,008 | 299,060,928 | 99.11 | 0.04 | 94.88 | 86.76 | 37.22 |
| LN7 | 549,489,888 | 505,356,480 | 91.97 | 0.03 | 95.66 | 88.5 | 38.08 |
| LN8 | 551,953,440 | 496,047,168 | 89.87 | 0.03 | 96.09 | 89.55 | 37.91 |
| LN9 | 540,245,088 | 495,981,504 | 91.81 | 0.04 | 94.91 | 86.81 | 37.97 |
| LS1 | 449,497,728 | 406,013,760 | 90.33 | 0.04 | 95.28 | 88.35 | 37.56 |
| LS2 | 459,269,568 | 425,379,744 | 92.62 | 0.04 | 94.2 | 86.27 | 37.73 |
| LS3 | 443,928,960 | 411,979,968 | 92.8 | 0.04 | 94.69 | 87.27 | 37.59 |
| LS4 | 423,871,776 | 377,649,792 | 89.1 | 0.04 | 94.31 | 86.53 | 37.68 |
| MS1 | 542,384,064 | 531,863,712 | 98.06 | 0.04 | 94.05 | 86.27 | 36.69 |
| MS10 | 583,827,840 | 566,620,704 | 97.05 | 0.04 | 93.65 | 85.46 | 37.11 |
| MS11 | 548,358,048 | 518,163,552 | 94.49 | 0.04 | 94.89 | 87.79 | 37.43 |
| MS12 | 584,034,912 | 553,796,640 | 94.82 | 0.04 | 94.09 | 86.26 | 37.69 |
| MS13 | 542,023,488 | 521,094,240 | 96.14 | 0.04 | 91.72 | 81.89 | 37.31 |
| MS14 | 568,739,520 | 542,823,552 | 95.44 | 0.04 | 92.59 | 83.49 | 37.67 |
| MS15 | 679,290,624 | 631,956,096 | 93.03 | 0.03 | 96.44 | 90.51 | 39.11 |
| MS2 | 492,559,488 | 482,905,152 | 98.04 | 0.04 | 93.6 | 85.4 | 36.66 |
| MS4 | 285,443,712 | 281,644,128 | 98.67 | 0.04 | 91.97 | 82.47 | 36.55 |
| MS5 | 489,882,816 | 450,963,072 | 92.06 | 0.04 | 92.21 | 82.73 | 37.73 |
| MS6 | 568,955,232 | 548,395,200 | 96.39 | 0.04 | 92.08 | 82.49 | 37.52 |
| MS7 | 519,310,944 | 506,336,256 | 97.5 | 0.04 | 93.23 | 84.68 | 37.11 |
| MS8 | 563,399,712 | 528,340,896 | 93.78 | 0.04 | 94.45 | 87 | 37.53 |
| MS9 | 530,380,800 | 511,109,280 | 96.37 | 0.04 | 93.68 | 85.47 | 37.14 |
| MX1 | 450,229,536 | 423,874,080 | 94.15 | 0.03 | 97.07 | 91.93 | 37.62 |
| MX10 | 413,438,976 | 398,166,912 | 96.31 | 0.03 | 97.06 | 91.87 | 37.84 |
| MX11 | 437,204,736 | 367,999,776 | 84.17 | 0.03 | 97.18 | 92.19 | 37.77 |
| MX12 | 377,715,168 | 326,359,584 | 86.4 | 0.03 | 97.1 | 92.05 | 37.84 |
| MX13 | 461,384,352 | 409,977,504 | 88.86 | 0.03 | 97.44 | 92.91 | 37.75 |
| MX14 | 420,864,192 | 373,491,648 | 88.74 | 0.03 | 97.25 | 92.38 | 37.92 |
| MX15 | 421,984,512 | 397,544,544 | 94.21 | 0.03 | 96.6 | 90.68 | 37.69 |
| MX2 | 442,561,824 | 367,786,080 | 83.1 | 0.03 | 97.27 | 92.53 | 37.82 |
| MX3 | 325,082,304 | 317,121,120 | 97.55 | 0.03 | 97 | 91.8 | 37.22 |
| MX4 | 345,313,728 | 340,693,344 | 98.66 | 0.03 | 96.85 | 91.35 | 36.69 |
| MX5 | 326,031,264 | 321,483,168 | 98.61 | 0.03 | 96.69 | 90.91 | 37.36 |
| MX6 | 462,371,904 | 428,113,728 | 92.59 | 0.03 | 96.76 | 91 | 38.3 |
| MX7 | 482,092,416 | 446,706,432 | 92.66 | 0.03 | 96.74 | 90.91 | 38.27 |
| MX8 | 408,184,704 | 367,517,952 | 90.04 | 0.03 | 97.03 | 91.81 | 38.19 |
| MX9 | 452,967,840 | 384,086,304 | 84.79 | 0.03 | 97.38 | 92.75 | 38.08 |
| SC1 | 275,807,520 | 253,366,272 | 91.86 | 0.03 | 96.67 | 91.01 | 39.12 |
| SC10 | 239,350,464 | 214,254,144 | 89.51 | 0.03 | 97.06 | 92.09 | 39.42 |
| SC11 | 231,467,616 | 203,024,448 | 87.71 | 0.03 | 97.24 | 92.48 | 39.47 |
| SC12 | 292,291,200 | 271,684,512 | 92.95 | 0.03 | 96.53 | 90.69 | 39.42 |
| SC13 | 254,362,176 | 229,449,888 | 90.21 | 0.03 | 96.61 | 90.94 | 39.2 |
| SC14 | 285,727,392 | 233,987,904 | 81.89 | 0.03 | 96.09 | 89.63 | 39.01 |
| SC15 | 293,474,592 | 275,527,008 | 93.88 | 0.03 | 95.52 | 88.23 | 38.77 |
| SC2 | 359,357,184 | 347,623,488 | 96.73 | 0.03 | 95.88 | 89.32 | 38.23 |
| SC3 | 290,136,096 | 259,808,256 | 89.55 | 0.03 | 96.54 | 90.82 | 38.76 |
| SC4 | 276,802,848 | 237,263,040 | 85.72 | 0.03 | 97.05 | 92.12 | 38.8 |
| SC5 | 279,650,880 | 232,156,800 | 83.02 | 0.03 | 96.7 | 91.27 | 38.91 |
| SC6 | 316,370,016 | 277,896,384 | 87.84 | 0.03 | 95.67 | 88.66 | 38.58 |
| SC7 | 290,739,744 | 235,944,000 | 81.15 | 0.03 | 96.16 | 89.88 | 38.73 |
| SC8 | 309,040,416 | 266,266,656 | 86.16 | 0.03 | 96.38 | 90.5 | 38.82 |
| SC9 | 316,790,208 | 295,307,424 | 93.22 | 0.03 | 95.72 | 88.66 | 39.06 |
| SN1 | 457,898,976 | 422,120,448 | 92.19 | 0.04 | 91.81 | 81.81 | 37.95 |
| SN10 | 371,632,320 | 361,276,416 | 97.21 | 0.06 | 88.95 | 76.59 | 38.2 |
| SN11 | 451,134,432 | 432,306,144 | 95.83 | 0.04 | 94.1 | 86.07 | 37.48 |
| SN12 | 499,744,800 | 445,840,128 | 89.21 | 0.04 | 94.23 | 86.39 | 37.72 |
| SN13 | 287,013,600 | 283,367,520 | 98.73 | 0.04 | 93.53 | 85.11 | 36.28 |
| SN14 | 427,870,080 | 413,651,808 | 96.68 | 0.04 | 93.53 | 85.11 | 36.98 |
| SN2 | 426,805,920 | 395,094,240 | 92.57 | 0.04 | 95.05 | 87.97 | 37.47 |
| SN3 | 418,160,736 | 400,820,256 | 95.85 | 0.04 | 95.4 | 88.63 | 37.41 |
| SN4 | 476,603,136 | 429,252,768 | 90.07 | 0.04 | 93.63 | 85.23 | 37.84 |
| SN5 | 447,670,368 | 426,152,736 | 95.19 | 0.04 | 93.87 | 85.66 | 37.42 |
| SN6 | 440,348,256 | 387,598,176 | 88.02 | 0.04 | 92.91 | 83.75 | 37.95 |
| SN7 | 299,507,616 | 294,585,120 | 98.36 | 0.04 | 91.3 | 80.92 | 37.05 |
| SN8 | 469,090,368 | 444,134,880 | 94.68 | 0.04 | 93.74 | 85.4 | 37.39 |
| SN9 | 440,239,104 | 419,406,336 | 95.27 | 0.04 | 92.57 | 83.14 | 37.84 |
| ST1 | 233,394,624 | 217,403,136 | 93.15 | 0.03 | 95.4 | 88.1 | 38.9 |
| ST10 | 117,184,032 | 113,663,808 | 97 | 0.03 | 96.13 | 90.1 | 38.23 |
| ST11 | 188,813,088 | 176,948,640 | 93.72 | 0.03 | 96.18 | 90.16 | 38.42 |
| ST12 | 188,654,688 | 158,482,368 | 84.01 | 0.03 | 96.03 | 89.82 | 38.8 |
| ST13 | 161,520,480 | 155,432,736 | 96.23 | 0.03 | 96.35 | 90.59 | 38.09 |
| ST15 | 215,274,240 | 189,410,112 | 87.99 | 0.03 | 96.25 | 90.28 | 39.07 |
| ST2 | 557,853,696 | 449,061,984 | 80.5 | 0.03 | 96.66 | 91.44 | 38.48 |
| ST3 | 605,583,072 | 495,396,576 | 81.8 | 0.03 | 96.52 | 91.08 | 38.61 |
| ST4 | 516,647,808 | 475,130,304 | 91.96 | 0.03 | 96.36 | 90.44 | 39.64 |
| ST5 | 140,780,736 | 137,614,752 | 97.75 | 0.03 | 96.07 | 89.89 | 38.06 |
| ST6 | 233,169,984 | 224,323,488 | 96.21 | 0.03 | 95.85 | 89.07 | 38.58 |
| ST7 | 194,382,432 | 188,370,720 | 96.91 | 0.04 | 94.07 | 85.01 | 38.38 |
| ST8 | 209,839,104 | 197,361,792 | 94.05 | 0.03 | 96.41 | 90.66 | 38.97 |
| ST9 | 214,089,120 | 203,274,720 | 94.95 | 0.03 | 96.44 | 90.69 | 38.82 |
| SZ1 | 365,349,024 | 344,416,032 | 94.27 | 0.03 | 95.67 | 88.43 | 38.99 |
| SZ10 | 234,677,952 | 226,401,696 | 96.47 | 0.05 | 90.59 | 79.02 | 38.63 |
| SZ11 | 339,776,928 | 332,653,248 | 97.9 | 0.03 | 95.66 | 88.43 | 38.42 |
| SZ12 | 372,508,416 | 360,792,288 | 96.85 | 0.03 | 95.84 | 88.86 | 38.32 |
| ST15 | 215,274,240 | 189,410,112 | 87.99 | 0.03 | 96.25 | 90.28 | 39.07 |
| ST2 | 557,853,696 | 449,061,984 | 80.5 | 0.03 | 96.66 | 91.44 | 38.48 |
| SZ13 | 677,085,696 | 620,327,808 | 91.62 | 0.03 | 96.34 | 90.2 | 39.36 |
| SZ14 | 286,596,288 | 283,338,144 | 98.86 | 0.03 | 95.43 | 87.93 | 38.24 |
| SZ15 | 352,684,800 | 342,851,904 | 97.21 | 0.03 | 95.65 | 88.37 | 39.08 |
| SZ2 | 330,967,296 | 325,085,184 | 98.22 | 0.04 | 94.14 | 85.22 | 38.65 |
| SZ3 | 616,497,696 | 540,226,368 | 87.63 | 0.03 | 95.41 | 88.08 | 39.58 |
| SZ4 | 641,482,560 | 589,032,576 | 91.82 | 0.03 | 95.38 | 87.95 | 39.77 |
| SZ5 | 369,856,224 | 359,890,848 | 97.31 | 0.04 | 93.07 | 83.29 | 38.09 |
| SZ6 | 344,426,400 | 337,827,168 | 98.08 | 0.03 | 95.44 | 87.95 | 38.18 |
| SZ7 | 590,529,888 | 547,301,088 | 92.68 | 0.03 | 96.08 | 89.63 | 39.47 |
| SZ8 | 587,289,888 | 539,134,848 | 91.8 | 0.03 | 96.8 | 91.34 | 39.41 |
| SZ9 | 435,439,584 | 418,762,944 | 96.17 | 0.04 | 94.12 | 85.22 | 38.36 |
| TB10 | 950,701,824 | 861,712,416 | 90.64 | 0.03 | 96.99 | 91.66 | 39.39 |
| TB11 | 256,781,952 | 237,749,472 | 92.59 | 0.03 | 95.48 | 88.28 | 38.66 |
| TB14 | 199,689,696 | 174,579,264 | 87.43 | 0.03 | 96.71 | 91.56 | 38.63 |
| TB15 | 182,844,288 | 151,424,064 | 82.82 | 0.03 | 96.91 | 92 | 38.63 |
| TB16 | 246,334,176 | 220,624,992 | 89.56 | 0.03 | 96.29 | 90.39 | 38.93 |
| TB17 | 237,616,992 | 211,666,464 | 89.08 | 0.03 | 96.34 | 90.56 | 38.72 |
| TB18 | 229,800,096 | 209,948,544 | 91.36 | 0.03 | 95.88 | 89.23 | 38.98 |
| TB2 | 659,423,808 | 590,649,120 | 89.57 | 0.03 | 96.91 | 91.56 | 38.51 |
| TB3 | 703,024,128 | 599,888,448 | 85.33 | 0.03 | 97.38 | 92.83 | 38.41 |
| TB4 | 696,682,080 | 592,077,312 | 84.99 | 0.03 | 97.03 | 91.88 | 38.23 |
| TB5 | 706,513,824 | 625,918,464 | 88.59 | 0.03 | 97.02 | 91.83 | 38.41 |
| TB6 | 665,075,232 | 576,931,968 | 86.75 | 0.03 | 97.53 | 93.23 | 38.11 |
| TB7 | 649,631,808 | 549,703,584 | 84.62 | 0.03 | 97.19 | 92.3 | 38.44 |
| TB8 | 702,847,872 | 569,437,632 | 81.02 | 0.03 | 96.05 | 89.43 | 38.21 |
| TB9 | 697,712,544 | 568,374,336 | 81.46 | 0.03 | 96.55 | 90.66 | 38.78 |
| TMS1 | 522,125,568 | 506,987,712 | 97.1 | 0.03 | 96.12 | 89.47 | 37.54 |
| TMS10 | 467,987,616 | 441,947,520 | 94.44 | 0.04 | 93.83 | 85.72 | 37.2 |
| TMS11 | 474,958,368 | 439,316,928 | 92.5 | 0.04 | 93.38 | 84.93 | 37.75 |
| TMS12 | 441,142,272 | 395,710,272 | 89.7 | 0.04 | 92.22 | 82.74 | 37.88 |
| TMS13 | 476,443,584 | 444,363,264 | 93.27 | 0.04 | 94.84 | 87.66 | 37.55 |
| TMS14 | 504,011,520 | 487,497,888 | 96.72 | 0.04 | 93.69 | 85.49 | 37.3 |
| TMS15 | 665,995,392 | 618,560,928 | 92.88 | 0.03 | 96.94 | 91.75 | 38.85 |
| TMS2 | 549,224,064 | 524,281,824 | 95.46 | 0.04 | 95.39 | 87.77 | 37.99 |
| TMS3 | 380,419,776 | 373,336,992 | 98.14 | 0.04 | 93.02 | 82.84 | 37.75 |
| TMS4 | 511,630,272 | 473,525,856 | 92.55 | 0.03 | 96.38 | 90.11 | 37.68 |
| TMS5 | 497,199,744 | 461,674,656 | 92.85 | 0.03 | 96.47 | 90.33 | 38.11 |
| TMS6 | 283,285,440 | 279,957,024 | 98.83 | 0.04 | 93.14 | 84.59 | 36.25 |
| TMS7 | 415,775,232 | 395,389,152 | 95.1 | 0.04 | 93.29 | 84.82 | 37.38 |
| TMS8 | 397,663,776 | 369,589,824 | 92.94 | 0.04 | 92.84 | 83.98 | 37.55 |
| TMS9 | 283,496,256 | 277,740,288 | 97.97 | 0.04 | 93.78 | 85.71 | 36.69 |
| TZ1 | 554,518,368 | 516,290,112 | 93.11 | 0.03 | 97.11 | 92.06 | 37.89 |
| TZ10 | 673,490,880 | 594,413,280 | 88.26 | 0.03 | 97.27 | 92.56 | 38.05 |
| TZ11 | 657,037,728 | 561,077,568 | 85.4 | 0.03 | 96.47 | 90.88 | 38.66 |
| TZ12 | 629,120,448 | 570,599,712 | 90.7 | 0.03 | 97.06 | 92.05 | 37.84 |
| TZ13 | 424,762,560 | 398,093,184 | 93.72 | 0.03 | 96.57 | 90.82 | 37.09 |
| TZ14 | 436,259,232 | 408,041,280 | 93.53 | 0.03 | 96.24 | 89.93 | 37.67 |
| TZ15 | 680,037,696 | 578,517,408 | 85.07 | 0.03 | 96.44 | 90.29 | 38.75 |
| TZ16 | 708,130,080 | 593,710,272 | 83.84 | 0.03 | 96.39 | 90.16 | 38.42 |
| TZ2 | 569,615,616 | 527,825,376 | 92.66 | 0.03 | 97.07 | 91.9 | 37.87 |
| TZ4 | 578,251,296 | 502,464,672 | 86.89 | 0.03 | 96.97 | 91.62 | 38.56 |
| TZ5 | 770,570,496 | 665,441,280 | 86.36 | 0.03 | 96.34 | 90 | 38.55 |
| TZ6 | 671,245,632 | 584,136,000 | 87.02 | 0.03 | 97.56 | 93.2 | 38.19 |
| TZ7 | 625,843,296 | 568,353,312 | 90.81 | 0.03 | 97.03 | 91.89 | 37.97 |
| TZ8 | 614,081,088 | 492,216,192 | 80.15 | 0.03 | 95.76 | 89.13 | 38.95 |
| TZ9 | 526,811,616 | 439,750,080 | 83.47 | 0.03 | 97.04 | 92.18 | 38.67 |
| XE1 | 363,157,056 | 341,758,368 | 94.11 | 0.03 | 96.41 | 90.1 | 39.09 |
| XE10 | 440,260,992 | 424,934,784 | 96.52 | 0.04 | 94.79 | 86.57 | 38.51 |
| XE11 | 228,195,072 | 225,504,576 | 98.82 | 0.03 | 95.27 | 87.67 | 38.09 |
| XE12 | 379,628,064 | 361,215,936 | 95.15 | 0.04 | 93.69 | 84.38 | 38.88 |
| XE13 | 374,755,968 | 342,726,624 | 91.45 | 0.03 | 96.55 | 90.46 | 39.08 |
| XE14 | 329,943,744 | 308,522,880 | 93.51 | 0.03 | 96.88 | 91.2 | 39.22 |
| XE15 | 369,745,056 | 354,837,024 | 95.97 | 0.03 | 95.44 | 87.91 | 38.93 |
| XE2 | 370,892,448 | 364,445,280 | 98.26 | 0.03 | 95.67 | 88.4 | 38.66 |
| XE3 | 698,288,832 | 639,245,088 | 91.54 | 0.03 | 96.29 | 90.24 | 38.83 |
| XE4 | 362,799,936 | 353,285,568 | 97.38 | 0.03 | 95.58 | 88.28 | 38.45 |
| XE5 | 440,005,536 | 424,143,936 | 96.4 | 0.03 | 96.66 | 90.73 | 38.86 |
| XE6 | 499,806,432 | 472,231,008 | 94.48 | 0.03 | 95.72 | 88.9 | 38.33 |
| XE7 | 437,448,960 | 425,107,584 | 97.18 | 0.03 | 96 | 89.17 | 38.8 |
| XE8 | 401,181,120 | 388,627,200 | 96.87 | 0.04 | 93.78 | 84.51 | 38.3 |
| XE9 | 471,772,800 | 458,296,992 | 97.14 | 0.04 | 95.28 | 87.86 | 39.13 |

**TABLE S4.** A statistical tabulation presenting the results of enzymatic digestion for 348 samples of *C. kousa* procured from 25 natural populations.

| Sample | Total PE clean reads | Total PE enzyme catch reads | Total PE enzyme cut completely reads | Enzyme catch ratio (%) | Enzyme cut completely ratio (%) |
| --- | --- | --- | --- | --- | --- |
| AHS1 | 968,281 | 963,697 | 918,298 | 99.5 | 95.3 |
| AHS10 | 1,938,548 | 1,926,320 | 1,812,546 | 99.4 | 94.1 |
| AHS11 | 1,309,289 | 1,298,392 | 1,224,369 | 99.2 | 94.3 |
| AHS12 | 1,606,437 | 1,595,709 | 1,507,033 | 99.3 | 94.4 |
| AHS13 | 1,265,958 | 1,225,851 | 1,165,474 | 96.8 | 95.1 |
| AHS14 | 1,312,352 | 1,284,677 | 1,206,583 | 97.9 | 93.9 |
| AHS15 | 1,227,410 | 1,212,162 | 1,153,120 | 98.8 | 95.1 |
| AHS2 | 1,602,116 | 1,592,285 | 1,510,058 | 99.4 | 94.8 |
| AHS3 | 1,123,028 | 1,116,821 | 1,053,509 | 99.4 | 94.3 |
| AHS4 | 1,730,357 | 1,709,341 | 1,623,644 | 98.8 | 95 |
| AHS5 | 907,452 | 903,503 | 855,554 | 99.6 | 94.7 |
| AHS6 | 1,020,999 | 1,015,238 | 952,137 | 99.4 | 93.8 |
| AHS7 | 1,140,207 | 1,132,937 | 1,037,805 | 99.4 | 91.6 |
| AHS8 | 2,256,409 | 2,230,672 | 2,094,435 | 98.9 | 93.9 |
| AHS9 | 1,644,472 | 1,607,113 | 1,456,859 | 97.7 | 90.7 |
| AK1 | 1,495,485 | 1,480,616 | 1,402,699 | 99 | 94.7 |
| AK10 | 973,646 | 967,175 | 915,677 | 99.3 | 94.7 |
| AK11 | 901,640 | 896,333 | 832,301 | 99.4 | 92.9 |
| AK12 | 1,469,361 | 1,456,450 | 1,373,433 | 99.1 | 94.3 |
| AK13 | 944,401 | 940,147 | 885,156 | 99.5 | 94.2 |
| AK14 | 925,539 | 917,903 | 873,875 | 99.2 | 95.2 |
| AK15 | 1,133,228 | 1,125,120 | 1,061,189 | 99.3 | 94.3 |
| AK2 | 973,476 | 967,887 | 899,254 | 99.4 | 92.9 |
| AK3 | 1,051,865 | 1,044,675 | 981,555 | 99.3 | 94 |
| AK4 | 1,149,347 | 1,136,158 | 1,052,146 | 98.9 | 92.6 |
| AK5 | 819,400 | 814,804 | 753,120 | 99.4 | 92.4 |
| AK6 | 1,836,529 | 1,817,099 | 1,720,706 | 98.9 | 94.7 |
| AK7 | 1,006,882 | 1,001,302 | 942,159 | 99.4 | 94.1 |
| AK8 | 869,622 | 863,971 | 773,025 | 99.4 | 89.5 |
| AK9 | 821,986 | 817,249 | 763,273 | 99.4 | 93.4 |
| BHS1 | 1,309,301 | 1,297,804 | 1,176,086 | 99.1 | 90.6 |
| BHS12 | 1,210,074 | 1,204,116 | 1,092,197 | 99.5 | 90.7 |
| BHS13 | 1,304,073 | 1,296,026 | 1,200,411 | 99.4 | 92.6 |
| BHS14 | 1,462,531 | 1,452,096 | 1,335,015 | 99.3 | 91.9 |
| BHS15 | 1,388,168 | 1,374,605 | 1,212,833 | 99 | 88.2 |
| BHS2 | 1,424,376 | 1,413,408 | 1,292,562 | 99.2 | 91.5 |
| BHS3 | 1,301,137 | 1,292,302 | 1,140,493 | 99.3 | 88.3 |
| BHS4 | 1,187,285 | 1,180,416 | 1,077,226 | 99.4 | 91.3 |
| BHS5 | 1,140,405 | 1,134,843 | 1,052,640 | 99.5 | 92.8 |
| BHS6 | 1,193,304 | 1,183,754 | 1,082,434 | 99.2 | 91.4 |
| BHS7 | 1,502,236 | 1,491,829 | 1,331,035 | 99.3 | 89.2 |
| ES1 | 1,384,133 | 1,370,787 | 1,274,589 | 99 | 93 |
| ES10 | 1,485,913 | 1,474,270 | 1,379,421 | 99.2 | 93.6 |
| ES11 | 619,684 | 615,972 | 572,140 | 99.4 | 92.9 |
| ES12 | 478,552 | 475,538 | 435,241 | 99.4 | 91.5 |
| ES13 | 633,992 | 628,802 | 577,208 | 99.2 | 91.8 |
| ES14 | 668,671 | 661,012 | 614,483 | 98.9 | 93 |
| ES15 | 736,264 | 731,255 | 672,608 | 99.3 | 92 |
| ES16 | 595,422 | 591,616 | 548,279 | 99.4 | 92.7 |
| ES2 | 1,417,948 | 1,397,627 | 1,299,833 | 98.6 | 93 |
| ES3 | 1,620,903 | 1,607,047 | 1,491,179 | 99.1 | 92.8 |
| ES4 | 1,506,376 | 1,495,349 | 1,383,755 | 99.3 | 92.5 |
| ES5 | 672,677 | 667,909 | 624,422 | 99.3 | 93.5 |
| ES7 | 592,409 | 589,302 | 558,337 | 99.5 | 94.7 |
| ES8 | 686,307 | 682,149 | 633,824 | 99.4 | 92.9 |
| ES9 | 623,139 | 620,123 | 583,046 | 99.5 | 94 |
| FN1 | 619,398 | 615,358 | 570,430 | 99.3 | 92.7 |
| FN10 | 846,225 | 838,353 | 782,229 | 99.1 | 93.3 |
| FN11 | 909,306 | 902,749 | 851,374 | 99.3 | 94.3 |
| FN12 | 1,030,761 | 1,014,012 | 952,029 | 98.4 | 93.9 |
| FN13 | 1,245,789 | 1,232,274 | 1,153,505 | 98.9 | 93.6 |
| FN14 | 929,529 | 908,913 | 847,467 | 97.8 | 93.2 |
| FN15 | 1,498,319 | 1,487,445 | 1,368,929 | 99.3 | 92 |
| FN2 | 593,872 | 590,136 | 529,903 | 99.4 | 89.8 |
| FN3 | 736,849 | 731,474 | 681,228 | 99.3 | 93.1 |
| FN4 | 611,121 | 606,979 | 568,534 | 99.3 | 93.7 |
| FN5 | 628,175 | 624,237 | 585,439 | 99.4 | 93.8 |
| FN6 | 732,821 | 727,211 | 668,219 | 99.2 | 91.9 |
| FN7 | 669,619 | 663,204 | 621,859 | 99.0 | 93.8 |
| FN8 | 703,438 | 697,858 | 655,579 | 99.2 | 93.9 |
| FN9 | 1,039,452 | 1,018,480 | 950,821 | 98.0 | 93.4 |
| HA1 | 2,010,586 | 1,986,821 | 1,865,323 | 98.8 | 93.9 |
| HA11 | 2,663,591 | 2,641,490 | 2,474,101 | 99.2 | 93.7 |
| HA12 | 1,994,034 | 1,980,709 | 1,801,475 | 99.3 | 91.0 |
| HA13 | 1,998,404 | 1,984,169 | 1,829,087 | 99.3 | 92.2 |
| HA14 | 2,014,772 | 2,000,562 | 1,881,655 | 99.3 | 94.1 |
| HA15 | 1,692,158 | 1,683,179 | 1,578,798 | 99.5 | 93.8 |
| HA16 | 1,342,493 | 1,329,395 | 1,250,476 | 99.0 | 94.1 |
| HA2 | 1,906,126 | 1,890,891 | 1,760,099 | 99.2 | 93.1 |
| HA3 | 1,801,096 | 1,777,192 | 1,661,155 | 98.7 | 93.5 |
| HA4 | 2,588,169 | 2,552,714 | 2,361,073 | 98.6 | 92.5 |
| HA5 | 1,925,501 | 1,911,834 | 1,715,736 | 99.3 | 89.7 |
| HA6 | 2,539,169 | 2,512,115 | 2,350,886 | 98.9 | 93.6 |
| HA7 | 1,527,169 | 1,514,234 | 1,400,882 | 99.2 | 92.5 |
| HA8 | 2,352,285 | 2,300,728 | 2,110,301 | 97.8 | 91.7 |
| HA9 | 2,155,371 | 2,138,114 | 1,988,901 | 99.2 | 93 |
| HG1 | 2,252,265 | 2,216,606 | 2,060,288 | 98.4 | 92.9 |
| HG10 | 2,211,617 | 2,185,488 | 2,019,982 | 98.8 | 92.4 |
| HG11 | 2,291,808 | 2,261,059 | 2,113,958 | 98.7 | 93.5 |
| HG12 | 1,873,173 | 1,857,976 | 1,755,876 | 99.2 | 94.5 |
| HG13 | 1,314,576 | 1,301,089 | 1,204,320 | 99.0 | 92.6 |
| HG14 | 1,354,228 | 1,345,371 | 1,102,295 | 99.3 | 81.9 |
| HG15 | 1,610,073 | 1,599,090 | 1,518,064 | 99.3 | 94.9 |
| HG16 | 1,468,868 | 1,457,012 | 1,365,466 | 99.2 | 93.7 |
| HG2 | 1,462,909 | 1,454,545 | 1,303,553 | 99.4 | 89.6 |
| HG3 | 1,443,393 | 1,434,016 | 1,333,459 | 99.4 | 93.0 |
| HG4 | 1,174,846 | 1,164,560 | 1,103,375 | 99.1 | 94.7 |
| HG5 | 1,327,185 | 1,318,853 | 1,149,970 | 99.4 | 87.2 |
| HG6 | 1,452,331 | 1,444,503 | 1,318,755 | 99.5 | 91.3 |
| HG7 | 1,496,489 | 1,485,299 | 1,366,153 | 99.3 | 92.0 |
| HG9 | 2,240,055 | 2,211,936 | 2,052,264 | 98.7 | 92.8 |
| HS1 | 1,738,567 | 1,725,661 | 1,607,433 | 99.3 | 93.1 |
| HS11 | 1,319,380 | 1,307,025 | 1,218,088 | 99.1 | 93.2 |
| HS12 | 1,633,713 | 1,619,248 | 1,476,697 | 99.1 | 91.2 |
| HS13 | 1,532,890 | 1,523,557 | 1,400,570 | 99.4 | 91.9 |
| HS14 | 1,345,126 | 1,330,621 | 1,250,711 | 98.9 | 94 |
| HS15 | 1,540,100 | 1,524,368 | 1,421,066 | 99 | 93.2 |
| HS16 | 1,488,967 | 1,480,860 | 1,394,265 | 99.5 | 94.2 |
| HS17 | 1,559,057 | 1,547,145 | 1,432,611 | 99.2 | 92.6 |
| HS2 | 1,486,474 | 1,472,256 | 1,387,606 | 99 | 94.3 |
| HS3 | 1,525,713 | 1,495,076 | 1,376,344 | 98 | 92.1 |
| HS4 | 1,603,081 | 1,591,149 | 1,477,526 | 99.3 | 92.9 |
| HS5 | 1,605,829 | 1,592,826 | 1,496,580 | 99.2 | 94 |
| HS6 | 1,564,218 | 1,549,824 | 1,375,450 | 99.1 | 88.7 |
| HS7 | 1,547,265 | 1,527,257 | 1,426,297 | 98.7 | 93.4 |
| HS8 | 1,326,597 | 1,314,707 | 1,209,690 | 99.1 | 92 |
| JG1 | 1,227,159 | 1,206,429 | 1,141,892 | 98.3 | 94.7 |
| JG11 | 1,754,554 | 1,732,139 | 1,606,974 | 98.7 | 92.8 |
| JG12 | 1,044,243 | 1,008,103 | 945,232 | 96.5 | 93.8 |
| JG13 | 1,162,169 | 1,135,535 | 1,027,562 | 97.7 | 90.5 |
| JG14 | 1,296,789 | 1,280,170 | 1,198,115 | 98.7 | 93.6 |
| JG15 | 1,302,755 | 1,291,507 | 1,226,895 | 99.1 | 95 |
| JG17 | 1,363,392 | 1,349,231 | 1,262,928 | 99 | 93.6 |
| JG18 | 857,918 | 848,413 | 779,363 | 98.9 | 91.9 |
| JG2 | 1,125,277 | 1,103,210 | 1,030,908 | 98 | 93.4 |
| JG3 | 1,037,137 | 1,030,053 | 977,940 | 99.3 | 94.9 |
| JG5 | 1,299,827 | 1,285,500 | 1,202,699 | 98.9 | 93.6 |
| JG6 | 1,203,707 | 1,194,744 | 1,133,825 | 99.3 | 94.9 |
| JG7 | 1,204,747 | 1,188,871 | 1,117,451 | 98.7 | 94 |
| JG8 | 1,038,432 | 1,023,576 | 928,952 | 98.6 | 90.8 |
| JG9 | 921,937 | 902,647 | 820,438 | 97.9 | 90.9 |
| JHS1 | 1,393,141 | 1,380,250 | 1,305,639 | 99.1 | 94.6 |
| JHS10 | 1,315,224 | 1,305,338 | 1,233,902 | 99.2 | 94.5 |
| JHS11 | 1,042,085 | 1,035,612 | 975,717 | 99.4 | 94.2 |
| JHS12 | 1,347,437 | 1,332,944 | 1,251,644 | 98.9 | 93.9 |
| JHS13 | 1,459,781 | 1,448,082 | 1,358,111 | 99.2 | 93.8 |
| JHS14 | 1,187,530 | 1,173,059 | 1,095,057 | 98.8 | 93.4 |
| JHS15 | 1,524,412 | 1,506,092 | 1,396,422 | 98.8 | 92.7 |
| JHS2 | 1,766,684 | 1,751,320 | 1,621,578 | 99.1 | 92.6 |
| JHS3 | 1,158,534 | 1,145,387 | 1,084,695 | 98.9 | 94.7 |
| JHS4 | 1,672,575 | 1,656,652 | 1,560,203 | 99 | 94.2 |
| JHS5 | 1,301,013 | 1,274,765 | 1,176,394 | 98 | 92.3 |
| JHS6 | 1,426,566 | 1,415,520 | 1,333,852 | 99.2 | 94.2 |
| JHS7 | 1,251,381 | 1,242,348 | 1,176,592 | 99.3 | 94.7 |
| JHS8 | 1,180,335 | 1,155,013 | 1,092,206 | 97.9 | 94.6 |
| JHS9 | 1,368,412 | 1,353,068 | 1,254,587 | 98.9 | 92.7 |
| JLS1 | 1,024,724 | 1,000,529 | 923,238 | 97.6 | 92.3 |
| JLS10 | 1,161,875 | 1,146,222 | 1,001,630 | 98.7 | 87.4 |
| JLS11 | 1,920,035 | 1,901,809 | 1,768,781 | 99.1 | 93 |
| JLS12 | 1,079,243 | 1,064,489 | 988,460 | 98.6 | 92.9 |
| JLS13 | 1,124,120 | 1,107,644 | 1,041,526 | 98.5 | 94 |
| JLS14 | 1,237,160 | 1,222,212 | 1,133,318 | 98.8 | 92.7 |
| JLS15 | 1,199,733 | 1,189,462 | 1,108,559 | 99.1 | 93.2 |
| JLS2 | 1,320,685 | 1,307,811 | 1,227,446 | 99 | 93.9 |
| JLS3 | 1,292,185 | 1,271,799 | 1,196,959 | 98.4 | 94.1 |
| JLS4 | 659,599 | 631,723 | 572,472 | 95.8 | 90.6 |
| JLS5 | 1,916,965 | 1,903,405 | 1,781,339 | 99.3 | 93.6 |
| JLS6 | 1,809,289 | 1,796,309 | 1,677,536 | 99.3 | 93.4 |
| JLS7 | 1,127,692 | 1,115,448 | 1,045,634 | 98.9 | 93.7 |
| JLS8 | 1,761,100 | 1,745,914 | 1,619,763 | 99.1 | 92.8 |
| JLS9 | 1,260,557 | 1,246,166 | 1,180,024 | 98.9 | 94.7 |
| JZJ1 | 1,632,509 | 1,590,347 | 1,430,620 | 97.4 | 90 |
| JZJ2 | 1,359,116 | 1,332,834 | 1,182,618 | 98.1 | 88.7 |
| JZJ3 | 1,761,066 | 1,734,231 | 1,537,628 | 98.5 | 88.7 |
| JZJ4 | 1,691,227 | 1,648,031 | 1,494,408 | 97.4 | 90.7 |
| JZJ5 | 1,833,119 | 1,776,600 | 1,584,177 | 96.9 | 89.2 |
| LH10 | 1,964,979 | 1,952,411 | 1,800,770 | 99.4 | 92.2 |
| LH11 | 2,018,209 | 2,000,207 | 1,881,482 | 99.1 | 94.1 |
| LH12 | 1,079,340 | 1,072,125 | 1,000,705 | 99.3 | 93.3 |
| LH13 | 1,645,091 | 1,620,714 | 1,503,334 | 98.5 | 92.8 |
| LH14 | 1,717,751 | 1,702,501 | 1,580,030 | 99.1 | 92.8 |
| LH15 | 890,515 | 885,656 | 829,831 | 99.5 | 93.7 |
| LH16 | 808,953 | 804,242 | 763,368 | 99.4 | 94.9 |
| LH2 | 1,486,939 | 1,478,612 | 1,400,915 | 99.4 | 94.7 |
| LH3 | 1,542,626 | 1,530,194 | 1,422,512 | 99.2 | 93 |
| LH4 | 1,503,206 | 1,489,710 | 1,331,264 | 99.1 | 89.4 |
| LH5 | 846,163 | 838,237 | 775,431 | 99.1 | 92.5 |
| LH6 | 1,050,743 | 1,039,204 | 982,204 | 98.9 | 94.5 |
| LH7 | 1,672,413 | 1,659,698 | 1,560,541 | 99.2 | 94 |
| LH8 | 1,522,944 | 1,504,981 | 1,407,545 | 98.8 | 93.5 |
| LH9 | 1,898,852 | 1,880,778 | 1,756,139 | 99 | 93.4 |
| LN1 | 1,704,975 | 1,694,222 | 1,608,383 | 99.4 | 94.9 |
| LN10 | 1,565,724 | 1,554,347 | 1,458,052 | 99.3 | 93.8 |
| LN11 | 1,175,107 | 1,168,525 | 1,103,680 | 99.4 | 94.5 |
| LN12 | 1,668,794 | 1,650,198 | 1,568,038 | 98.9 | 95 |
| LN13 | 1,846,926 | 1,831,122 | 1,722,146 | 99.1 | 94 |
| LN14 | 1,673,489 | 1,651,540 | 1,561,584 | 98.7 | 94.6 |
| LN15 | 1,684,643 | 1,661,656 | 1,547,936 | 98.6 | 93.2 |
| LN2 | 1,492,713 | 1,480,937 | 1,398,569 | 99.2 | 94.4 |
| LN3 | 1,832,044 | 1,817,450 | 1,706,944 | 99.2 | 93.9 |
| LN4 | 1,797,800 | 1,784,412 | 1,697,209 | 99.3 | 95.1 |
| LN5 | 1,647,153 | 1,633,590 | 1,550,728 | 99.2 | 94.9 |
| LN6 | 1,038,406 | 1,025,830 | 963,411 | 98.8 | 93.9 |
| LN7 | 1,754,710 | 1,730,168 | 1,635,959 | 98.6 | 94.6 |
| LN8 | 1,722,386 | 1,701,604 | 1,599,626 | 98.8 | 94 |
| LN9 | 1,722,158 | 1,700,307 | 1,592,824 | 98.7 | 93.7 |
| LS1 | 1,409,770 | 1,393,536 | 1,280,793 | 98.8 | 91.9 |
| LS2 | 1,477,013 | 1,451,784 | 1,305,224 | 98.3 | 89.9 |
| LS3 | 1,430,486 | 1,410,653 | 1,295,217 | 98.6 | 91.8 |
| LS4 | 1,311,284 | 1,283,542 | 1,163,960 | 97.9 | 90.7 |
| MS1 | 1,846,749 | 1,820,254 | 1,623,579 | 98.6 | 89.2 |
| MS10 | 1,967,433 | 1,932,205 | 1,724,009 | 98.2 | 89.2 |
| MS11 | 1,799,179 | 1,773,363 | 1,613,673 | 98.6 | 91 |
| MS12 | 1,922,905 | 1,888,553 | 1,716,950 | 98.2 | 90.9 |
| MS13 | 1,809,355 | 1,754,573 | 1,535,306 | 97 | 87.5 |
| MS14 | 1,884,804 | 1,829,472 | 1,637,500 | 97.1 | 89.5 |
| MS15 | 2,194,292 | 2,164,923 | 2,044,535 | 98.7 | 94.4 |
| MS2 | 1,676,754 | 1,641,190 | 1,461,324 | 97.9 | 89 |
| MS4 | 977,931 | 942,593 | 823,891 | 96.4 | 87.4 |
| MS5 | 1,565,844 | 1,500,333 | 1,336,926 | 95.8 | 89.1 |
| MS6 | 1,904,150 | 1,841,098 | 1,630,256 | 96.7 | 88.5 |
| MS7 | 1,758,112 | 1,722,640 | 1,535,109 | 98 | 89.1 |
| MS8 | 1,834,517 | 1,807,834 | 1,633,177 | 98.5 | 90.3 |
| MS9 | 1,774,685 | 1,744,484 | 1,561,332 | 98.3 | 89.5 |
| MX1 | 1,471,785 | 1,463,926 | 1,332,994 | 99.5 | 91.1 |
| MX10 | 1,382,524 | 1,373,620 | 1,167,267 | 99.4 | 85 |
| MX11 | 1,277,777 | 1,271,277 | 1,046,072 | 99.5 | 82.3 |
| MX12 | 1,133,193 | 1,126,199 | 1,019,539 | 99.4 | 90.5 |
| MX13 | 1,423,533 | 1,415,721 | 1,281,527 | 99.5 | 90.5 |
| MX14 | 1,296,846 | 1,289,121 | 1,133,062 | 99.4 | 87.9 |
| MX15 | 1,380,363 | 1,371,282 | 1,180,749 | 99.3 | 86.1 |
| MX2 | 1,277,035 | 1,271,473 | 1,159,689 | 99.6 | 91.2 |
| MX3 | 1,101,115 | 1,093,784 | 1,035,493 | 99.3 | 94.7 |
| MX4 | 1,182,963 | 1,175,930 | 960,317 | 99.4 | 81.7 |
| MX5 | 1,116,261 | 1,107,790 | 1,034,731 | 99.2 | 93.4 |
| MX6 | 1,486,506 | 1,470,295 | 1,387,142 | 98.9 | 94.3 |
| MX7 | 1,551,064 | 1,540,671 | 1,411,427 | 99.3 | 91.6 |
| MX8 | 1,276,104 | 1,268,959 | 1,174,087 | 99.4 | 92.5 |
| MX9 | 1,333,633 | 1,326,533 | 1,218,676 | 99.5 | 91.9 |
| SC1 | 879,744 | 874,828 | 809,661 | 99.4 | 92.6 |
| SC10 | 743,938 | 739,996 | 700,604 | 99.5 | 94.7 |
| SC11 | 704,946 | 701,441 | 665,112 | 99.5 | 94.8 |
| SC12 | 943,349 | 935,921 | 877,708 | 99.2 | 93.8 |
| SC13 | 796,701 | 791,507 | 740,846 | 99.3 | 93.6 |
| SC14 | 812,458 | 806,337 | 750,836 | 99.2 | 93.1 |
| SC15 | 956,691 | 949,627 | 879,854 | 99.3 | 92.7 |
| SC2 | 1,207,026 | 1,195,080 | 1,113,676 | 99 | 93.2 |
| SC3 | 902,112 | 896,767 | 829,563 | 99.4 | 92.5 |
| SC4 | 823,830 | 819,034 | 759,861 | 99.4 | 92.8 |
| SC5 | 806,100 | 801,707 | 750,904 | 99.5 | 93.7 |
| SC6 | 964,918 | 957,729 | 874,613 | 99.3 | 91.3 |
| SC7 | 819,250 | 812,539 | 757,709 | 99.2 | 93.3 |
| SC8 | 924,537 | 917,902 | 853,423 | 99.3 | 93 |
| SC9 | 1,025,373 | 1,018,319 | 955,129 | 99.3 | 93.8 |
| SN1 | 1,465,696 | 1,417,653 | 1,273,670 | 96.7 | 89.8 |
| SN10 | 1,254,432 | 1,188,632 | 1,048,572 | 94.8 | 88.2 |
| SN11 | 1,501,063 | 1,473,482 | 1,336,618 | 98.2 | 90.7 |
| SN12 | 1,548,056 | 1,520,290 | 1,372,130 | 98.2 | 90.3 |
| SN13 | 983,915 | 961,023 | 801,408 | 97.7 | 83.4 |
| SN14 | 1,436,291 | 1,402,415 | 1,257,994 | 97.6 | 89.7 |
| SN2 | 1,371,855 | 1,349,059 | 1,247,617 | 98.3 | 92.5 |
| SN3 | 1,391,737 | 1,372,989 | 1,279,841 | 98.7 | 93.2 |
| SN4 | 1,490,461 | 1,451,160 | 1,315,434 | 97.4 | 90.6 |
| SN5 | 1,479,697 | 1,449,304 | 1,329,803 | 97.9 | 91.8 |
| SN6 | 1,345,827 | 1,305,580 | 1,182,127 | 97 | 90.5 |
| SN7 | 1,022,865 | 985,929 | 877,698 | 96.4 | 89 |
| SN8 | 1,542,135 | 1,515,077 | 1,353,646 | 98.2 | 89.3 |
| SN9 | 1,456,272 | 1,418,625 | 1,288,955 | 97.4 | 90.9 |
| ST1 | 754,872 | 749,130 | 696,872 | 99.2 | 93 |
| ST10 | 394,666 | 391,672 | 357,640 | 99.2 | 91.3 |
| ST11 | 614,405 | 609,956 | 563,091 | 99.3 | 92.3 |
| ST12 | 550,286 | 546,550 | 508,311 | 99.3 | 93 |
| ST13 | 539,697 | 535,967 | 493,401 | 99.3 | 92.1 |
| ST14 | 578,477 | 575,313 | 528,845 | 99.5 | 91.9 |
| ST15 | 657,674 | 652,026 | 610,201 | 99.1 | 93.6 |
| ST2 | 1,559,243 | 1,548,785 | 1,426,359 | 99.3 | 92.1 |
| ST3 | 1,720,127 | 1,704,265 | 1,561,197 | 99.1 | 91.6 |
| ST4 | 1,649,758 | 1,635,196 | 1,505,438 | 99.1 | 92.1 |
| ST5 | 477,829 | 474,433 | 440,518 | 99.3 | 92.9 |
| ST6 | 778,901 | 773,183 | 730,262 | 99.3 | 94.4 |
| ST7 | 654,065 | 646,169 | 597,906 | 98.8 | 92.5 |
| ST8 | 685,284 | 680,601 | 634,802 | 99.3 | 93.3 |
| ST9 | 705,815 | 700,774 | 659,115 | 99.3 | 94.1 |
| SZ1 | 1,195,889 | 1,183,236 | 1,128,046 | 98.9 | 95.3 |
| SZ10 | 786,117 | 754,692 | 687,928 | 96 | 91.2 |
| SZ11 | 1,155,046 | 1,142,828 | 1,080,169 | 98.9 | 94.5 |
| SZ12 | 1,252,751 | 1,238,497 | 1,175,384 | 98.9 | 94.9 |
| SZ13 | 2,153,916 | 2,132,665 | 1,993,578 | 99 | 93.5 |
| SZ14 | 983,813 | 969,793 | 897,804 | 98.6 | 92.6 |
| SZ15 | 1,190,458 | 1,175,259 | 1,111,834 | 98.7 | 94.6 |
| SZ2 | 1,128,768 | 1,102,550 | 1,048,523 | 97.7 | 95.1 |
| SZ3 | 1,875,786 | 1,838,042 | 1,713,773 | 98 | 93.2 |
| SZ4 | 2,045,252 | 2,018,504 | 1,893,362 | 98.7 | 93.8 |
| SZ5 | 1,249,621 | 1,220,987 | 1,139,071 | 97.7 | 93.3 |
| SZ6 | 1,173,011 | 1,161,961 | 1,089,894 | 99.1 | 93.8 |
| SZ7 | 1,900,351 | 1,882,702 | 1,775,494 | 99.1 | 94.3 |
| SZ8 | 1,871,996 | 1,856,116 | 1,758,624 | 99.2 | 94.7 |
| SZ9 | 1,454,038 | 1,432,201 | 1,357,217 | 98.5 | 94.8 |
| TB10 | 2,992,057 | 2,968,075 | 2,789,047 | 99.2 | 94 |
| TB11 | 825,519 | 819,403 | 762,900 | 99.3 | 93.1 |
| TB14 | 606,178 | 602,052 | 561,077 | 99.3 | 93.2 |
| TB15 | 525,778 | 522,449 | 486,359 | 99.4 | 93.1 |
| TB16 | 766,059 | 759,236 | 712,428 | 99.1 | 93.8 |
| TB17 | 734,953 | 729,752 | 679,306 | 99.3 | 93.1 |
| TB18 | 728,988 | 722,699 | 681,048 | 99.1 | 94.2 |
| TB2 | 2,050,865 | 2,038,127 | 1,891,207 | 99.4 | 92.8 |
| TB3 | 2,082,946 | 2,071,488 | 1,941,065 | 99.4 | 93.7 |
| TB4 | 2,055,824 | 2,044,148 | 1,896,638 | 99.4 | 92.8 |
| TB5 | 2,173,328 | 2,159,884 | 2,018,498 | 99.4 | 93.5 |
| TB6 | 2,003,236 | 1,989,894 | 1,855,824 | 99.3 | 93.3 |
| TB7 | 1,908,693 | 1,896,023 | 1,770,960 | 99.3 | 93.4 |
| TB8 | 1,977,214 | 1,960,182 | 1,788,167 | 99.1 | 91.2 |
| TB9 | 1,973,522 | 1,951,785 | 1,812,040 | 98.9 | 92.8 |
| TMS1 | 1,760,374 | 1,747,276 | 1,627,212 | 99.3 | 93.1 |
| TMS10 | 1,534,540 | 1,511,974 | 1,348,253 | 98.5 | 89.2 |
| TMS11 | 1,525,406 | 1,487,977 | 1,346,734 | 97.5 | 90.5 |
| TMS12 | 1,373,994 | 1,334,081 | 1,188,490 | 97.1 | 89.1 |
| TMS13 | 1,542,928 | 1,525,352 | 1,397,011 | 98.9 | 91.6 |
| TMS14 | 1,692,701 | 1,663,160 | 1,489,416 | 98.3 | 89.6 |
| TMS15 | 2,147,781 | 2,131,649 | 2,012,257 | 99.2 | 94.4 |
| TMS2 | 1,820,423 | 1,801,472 | 1,703,803 | 99 | 94.6 |
| TMS3 | 1,296,309 | 1,269,434 | 1,168,207 | 97.9 | 92 |
| TMS4 | 1,644,187 | 1,631,612 | 1,511,380 | 99.2 | 92.6 |
| TMS5 | 1,603,037 | 1,590,186 | 1,503,510 | 99.2 | 94.5 |
| TMS6 | 972,073 | 950,491 | 810,789 | 97.8 | 85.3 |
| TMS7 | 1,372,879 | 1,342,773 | 1,193,336 | 97.8 | 88.9 |
| TMS8 | 1,283,298 | 1,252,099 | 1,117,815 | 97.6 | 89.3 |
| TMS9 | 964,376 | 946,706 | 841,036 | 98.2 | 88.8 |
| TZ1 | 1,792,674 | 1,781,524 | 1,638,431 | 99.4 | 92 |
| TZ10 | 2,063,935 | 2,054,193 | 1,921,611 | 99.5 | 93.5 |
| TZ11 | 1,948,186 | 1,934,814 | 1,790,605 | 99.3 | 92.5 |
| TZ12 | 1,981,249 | 1,969,165 | 1,833,423 | 99.4 | 93.1 |
| TZ13 | 1,382,268 | 1,372,819 | 1,251,979 | 99.3 | 91.2 |
| TZ14 | 1,416,810 | 1,402,434 | 1,289,683 | 99 | 92 |
| TZ15 | 2,008,741 | 1,982,944 | 1,855,548 | 98.7 | 93.6 |
| TZ16 | 2,061,494 | 2,045,323 | 1,883,863 | 99.2 | 92.1 |
| TZ2 | 1,832,727 | 1,823,956 | 1,692,506 | 99.5 | 92.8 |
| TZ4 | 1,744,669 | 1,729,829 | 1,625,384 | 99.1 | 94 |
| TZ5 | 2,310,560 | 2,292,901 | 2,138,181 | 99.2 | 93.3 |
| TZ6 | 2,028,250 | 2,018,069 | 1,896,777 | 99.5 | 94 |
| TZ7 | 1,973,449 | 1,961,547 | 1,833,278 | 99.4 | 93.5 |
| TZ8 | 1,709,084 | 1,694,265 | 1,551,982 | 99.1 | 91.6 |
| TZ9 | 1,526,910 | 1,517,552 | 1,408,626 | 99.4 | 92.8 |
| XE1 | 1,186,661 | 1,176,793 | 1,124,535 | 99.2 | 95.6 |
| XE10 | 1,475,468 | 1,444,880 | 1,373,824 | 97.9 | 95.1 |
| XE11 | 783,002 | 769,736 | 730,958 | 98.3 | 95 |
| XE12 | 1,254,222 | 1,229,634 | 1,155,528 | 98 | 94 |
| XE13 | 1,190,023 | 1,178,342 | 1,118,530 | 99 | 94.9 |
| XE14 | 1,071,260 | 1,063,418 | 1,020,639 | 99.3 | 96 |
| XE15 | 1,232,073 | 1,213,375 | 1,157,112 | 98.5 | 95.4 |
| XE2 | 1,265,435 | 1,252,429 | 1,189,938 | 99 | 95 |
| XE3 | 2,219,601 | 2,193,945 | 2,051,906 | 98.8 | 93.5 |
| XE4 | 1,226,686 | 1,212,976 | 1,147,373 | 98.9 | 94.6 |
| XE5 | 1,472,722 | 1,458,931 | 1,396,459 | 99.1 | 95.7 |
| XE6 | 1,639,691 | 1,624,120 | 1,497,789 | 99.1 | 92.2 |
| XE7 | 1,476,068 | 1,458,809 | 1,398,294 | 98.8 | 95.9 |
| XE8 | 1,349,400 | 1,324,131 | 1,246,372 | 98.1 | 94.1 |
| XE9 | 1,591,309 | 1,567,109 | 1,478,773 | 98.5 | 94.4 |

**TABLE S5.** A statistical table presenting the tag fragments of 348 samples of *C. kousa* sourced from 25 natural populations.

| Sample | Total Tag number | Tag ≥ 4 | Tag ≥ 8 | Tag ≥ 4 average |
| --- | --- | --- | --- | --- |
| AHS1 | 1,448,464 | 122,031 | 47,721 | 11.87 |
| AHS10 | 2,840,209 | 224,456 | 98,428 | 12.654 |
| AHS11 | 2,012,360 | 175,429 | 68,124 | 11.471 |
| AHS12 | 2,451,724 | 210,164 | 88,618 | 11.666 |
| AHS13 | 2,031,519 | 164,779 | 74,645 | 12.329 |
| AHS14 | 2,041,053 | 166,233 | 73,957 | 12.278 |
| AHS15 | 1,958,266 | 160,291 | 73,611 | 12.217 |
| AHS2 | 2,513,891 | 199,459 | 89,571 | 12.604 |
| AHS3 | 1,740,319 | 142,676 | 62,003 | 12.198 |
| AHS4 | 2,737,271 | 211,906 | 96,769 | 12.917 |
| AHS5 | 1,304,384 | 114,743 | 43,689 | 11.368 |
| AHS6 | 1,496,145 | 125,046 | 50,410 | 11.965 |
| AHS7 | 1,723,600 | 133,261 | 59,267 | 12.934 |
| AHS8 | 3,598,455 | 255,842 | 133,794 | 14.065 |
| AHS9 | 2,645,645 | 188,769 | 96,267 | 14.015 |
| AK1 | 2,277,299 | 192,460 | 82,068 | 11.833 |
| AK10 | 1,468,422 | 125,799 | 50,144 | 11.673 |
| AK11 | 1,323,865 | 115,028 | 45,864 | 11.509 |
| AK12 | 2,297,646 | 192,955 | 85,592 | 11.908 |
| AK13 | 1,435,201 | 124,652 | 50,008 | 11.514 |
| AK14 | 1,370,356 | 117,936 | 45,067 | 11.619 |
| AK15 | 1,717,555 | 143,492 | 59,565 | 11.97 |
| AK2 | 1,423,479 | 122,112 | 46,981 | 11.657 |
| AK3 | 1,499,536 | 138,227 | 50,282 | 10.848 |
| AK4 | 1,781,240 | 158,618 | 64,485 | 11.23 |
| AK5 | 1,020,513 | 97,214 | 32,892 | 10.498 |
| AK6 | 2,849,331 | 213,808 | 100,331 | 13.327 |
| AK7 | 1,497,672 | 130,434 | 53,076 | 11.482 |
| AK8 | 1,238,853 | 106,587 | 41,756 | 11.623 |
| AK9 | 1,157,499 | 104,293 | 38,511 | 11.099 |
| BHS1 | 1,626,172 | 155,270 | 49,869 | 10.473 |
| BHS12 | 1,629,674 | 156,170 | 54,951 | 10.435 |
| BHS13 | 1,756,234 | 163,880 | 55,258 | 10.717 |
| BHS14 | 2,123,666 | 195,898 | 81,030 | 10.841 |
| BHS15 | 1,944,396 | 180,163 | 68,012 | 10.792 |
| BHS2 | 1,995,859 | 183,214 | 68,490 | 10.894 |
| BHS3 | 1,762,531 | 163,082 | 55,949 | 10.808 |
| BHS4 | 1,490,807 | 146,946 | 44,773 | 10.145 |
| BHS5 | 1,444,621 | 143,547 | 42,875 | 10.064 |
| BHS6 | 1,360,228 | 133,935 | 38,376 | 10.156 |
| BHS7 | 2,086,935 | 191,504 | 74,549 | 10.898 |
| ES1 | 1,888,995 | 160,254 | 64,708 | 11.788 |
| ES10 | 2,107,834 | 171,803 | 68,882 | 12.269 |
| ES11 | 706,040 | 69,854 | 18,276 | 10.107 |
| ES12 | 560,903 | 57,552 | 14,709 | 9.746 |
| ES13 | 765,144 | 76,896 | 20,074 | 9.95 |
| ES14 | 736,413 | 72,187 | 17,258 | 10.201 |
| ES15 | 859,736 | 84,800 | 22,551 | 10.138 |
| ES16 | 689,293 | 74,325 | 18,461 | 9.274 |
| ES2 | 1,837,144 | 153,383 | 59,755 | 11.977 |
| ES3 | 2,214,071 | 178,618 | 74,462 | 12.396 |
| ES4 | 2,058,306 | 170,780 | 67,909 | 12.052 |
| ES5 | 792,568 | 79,577 | 20,112 | 9.96 |
| ES7 | 695,072 | 65,130 | 16,022 | 10.672 |
| ES8 | 861,742 | 81,636 | 22,304 | 10.556 |
| ES9 | 728,072 | 71,797 | 18,034 | 10.141 |
| FN1 | 644,611 | 63,930 | 15,832 | 10.083 |
| FN10 | 856,587 | 88,580 | 19,668 | 9.67 |
| FN11 | 1,027,362 | 106,469 | 24,971 | 9.649 |
| FN12 | 1,237,526 | 125,146 | 31,236 | 9.889 |
| FN13 | 1,797,601 | 176,660 | 56,524 | 10.175 |
| FN14 | 1,038,051 | 107,918 | 24,669 | 9.619 |
| FN15 | 1,876,940 | 161,793 | 61,641 | 11.601 |
| FN2 | 592,766 | 61,198 | 14,489 | 9.686 |
| FN3 | 908,261 | 91,482 | 26,577 | 9.928 |
| FN4 | 690,821 | 71,579 | 19,196 | 9.651 |
| FN5 | 700,546 | 69,190 | 17,569 | 10.125 |
| FN6 | 876,195 | 87,065 | 23,213 | 10.064 |
| FN7 | 777,542 | 78,113 | 19,098 | 9.954 |
| FN8 | 899,339 | 91,658 | 27,272 | 9.812 |
| FN9 | 1,333,939 | 142,290 | 38,068 | 9.375 |
| HA1 | 3,029,001 | 212,687 | 100,752 | 14.242 |
| HA11 | 4,315,656 | 259,889 | 139,176 | 16.606 |
| HA12 | 3,084,618 | 216,376 | 107,601 | 14.256 |
| HA13 | 2,960,042 | 221,427 | 98,884 | 13.368 |
| HA14 | 2,871,978 | 215,727 | 93,325 | 13.313 |
| HA15 | 2,654,743 | 205,950 | 98,184 | 12.89 |
| HA16 | 1,887,628 | 155,387 | 64,335 | 12.148 |
| HA2 | 2,699,907 | 192,821 | 82,728 | 14.002 |
| HA3 | 2,569,908 | 191,703 | 84,989 | 13.406 |
| HA4 | 4,063,339 | 255,606 | 136,720 | 15.897 |
| HA5 | 2,234,015 | 182,520 | 67,130 | 12.24 |
| HA6 | 3,956,399 | 255,940 | 133,554 | 15.458 |
| HA7 | 2,198,592 | 178,634 | 79,662 | 12.308 |
| HA8 | 3,778,919 | 241,086 | 130,653 | 15.675 |
| HA9 | 3,218,541 | 234,511 | 111,845 | 13.724 |
| HG1 | 3,201,568 | 225,351 | 102,984 | 14.207 |
| HG10 | 3,116,558 | 225,546 | 99,937 | 13.818 |
| HG11 | 3,565,253 | 235,221 | 117,230 | 15.157 |
| HG12 | 2,755,066 | 211,003 | 90,707 | 13.057 |
| HG13 | 1,658,922 | 147,316 | 54,531 | 11.261 |
| HG14 | 1,662,388 | 160,892 | 52,961 | 10.332 |
| HG15 | 2,366,475 | 183,683 | 73,188 | 12.883 |
| HG16 | 1,983,765 | 164,761 | 65,066 | 12.040 |
| HG2 | 1,950,186 | 173,080 | 61,794 | 11.268 |
| HG3 | 2,111,931 | 179,571 | 66,086 | 11.761 |
| HG4 | 1,529,757 | 146,710 | 46,744 | 10.427 |
| HG5 | 1,589,563 | 149,620 | 47,698 | 10.624 |
| HG6 | 1,837,887 | 171,918 | 58,162 | 10.690 |
| HG7 | 2,172,822 | 178,928 | 80,800 | 12.144 |
| HG9 | 3,460,612 | 240,419 | 119,512 | 14.394 |
| HS1 | 2,857,252 | 243,246 | 108,911 | 11.746 |
| HS11 | 1,784,366 | 173,722 | 53,683 | 10.271 |
| HS12 | 2,523,715 | 223,242 | 93,972 | 11.305 |
| HS13 | 2,312,503 | 211,646 | 83,162 | 10.926 |
| HS14 | 1,924,417 | 186,837 | 61,293 | 10.3 |
| HS15 | 2,311,597 | 211,061 | 78,490 | 10.952 |
| HS16 | 2,200,255 | 206,163 | 72,627 | 10.672 |
| HS17 | 2,365,207 | 218,402 | 85,931 | 10.83 |
| HS2 | 2,289,321 | 208,638 | 74,943 | 10.973 |
| HS3 | 2,362,552 | 220,280 | 86,697 | 10.725 |
| HS4 | 2,466,242 | 219,022 | 86,735 | 11.26 |
| HS5 | 2,506,517 | 228,640 | 91,405 | 10.963 |
| HS6 | 2,206,933 | 206,721 | 82,797 | 10.676 |
| HS7 | 2,063,639 | 172,217 | 69,574 | 11.983 |
| HS8 | 1,854,629 | 183,641 | 59,174 | 10.099 |
| JG1 | 1,754,435 | 176,946 | 54,748 | 9.915 |
| JG11 | 2,282,015 | 190,235 | 79,121 | 11.996 |
| JG12 | 1,294,281 | 138,477 | 35,657 | 9.347 |
| JG13 | 1,493,613 | 154,202 | 43,283 | 9.686 |
| JG14 | 1,893,412 | 183,721 | 61,433 | 10.306 |
| JG15 | 1,926,450 | 191,211 | 67,648 | 10.075 |
| JG17 | 1,964,846 | 195,881 | 68,315 | 10.031 |
| JG18 | 914,598 | 100,704 | 24,355 | 9.082 |
| JG2 | 1,581,961 | 158,142 | 46,771 | 10.003 |
| JG3 | 1,373,212 | 138,263 | 37,444 | 9.932 |
| JG5 | 1,938,959 | 187,177 | 66,536 | 10.359 |
| JG6 | 1,690,454 | 167,273 | 51,866 | 10.106 |
| JG7 | 1,723,546 | 169,566 | 55,525 | 10.164 |
| JG8 | 1,445,469 | 144,619 | 48,393 | 9.995 |
| JG9 | 1,009,910 | 109,602 | 28,548 | 9.214 |
| JHS1 | 2,115,915 | 196,663 | 74,011 | 10.759 |
| JHS10 | 1,896,407 | 185,629 | 61,498 | 10.216 |
| JHS11 | 1,288,878 | 130,903 | 32,846 | 9.846 |
| JHS12 | 1,894,119 | 182,820 | 60,426 | 10.361 |
| JHS13 | 2,152,778 | 207,915 | 74,200 | 10.354 |
| JHS14 | 1,532,363 | 156,037 | 43,261 | 9.821 |
| JHS15 | 2,374,024 | 216,964 | 84,918 | 10.942 |
| JHS2 | 2,383,384 | 193,919 | 82,275 | 12.291 |
| JHS3 | 1,598,638 | 157,972 | 47,585 | 10.12 |
| JHS4 | 2,524,610 | 192,332 | 87,266 | 13.126 |
| JHS5 | 1,911,214 | 185,932 | 64,707 | 10.279 |
| JHS6 | 1,989,576 | 169,895 | 67,305 | 11.711 |
| JHS7 | 1,745,550 | 150,491 | 56,572 | 11.599 |
| JHS8 | 1,651,648 | 165,662 | 50,361 | 9.97 |
| JHS9 | 1,925,832 | 190,162 | 62,726 | 10.127 |
| JLS1 | 1,371,799 | 144,886 | 42,606 | 9.468 |
| JLS10 | 1,570,812 | 156,449 | 55,812 | 10.04 |
| JLS11 | 2,852,596 | 223,454 | 106,429 | 12.766 |
| JLS12 | 1,379,589 | 146,997 | 41,853 | 9.385 |
| JLS13 | 1,494,853 | 154,728 | 44,108 | 9.661 |
| JLS14 | 1,751,789 | 172,608 | 59,749 | 10.149 |
| JLS15 | 1,742,481 | 179,943 | 58,614 | 9.684 |
| JLS2 | 1,949,013 | 192,499 | 69,310 | 10.125 |
| JLS3 | 1,916,362 | 193,794 | 66,224 | 9.889 |
| JLS4 | 694,237 | 79,318 | 15,754 | 8.753 |
| JLS5 | 2,792,858 | 226,169 | 102,438 | 12.349 |
| JLS6 | 2,529,874 | 217,736 | 93,385 | 11.619 |
| JLS7 | 1,648,953 | 172,783 | 60,000 | 9.543 |
| JLS8 | 2,334,117 | 195,779 | 82,692 | 11.922 |
| JLS9 | 1,893,276 | 184,330 | 64,451 | 10.271 |
| JZJ1 | 2,340,018 | 231,343 | 88,156 | 10.115 |
| JZJ2 | 1,981,054 | 197,529 | 78,467 | 10.029 |
| JZJ3 | 2,659,579 | 242,973 | 105,237 | 10.946 |
| JZJ4 | 2,479,730 | 233,927 | 91,762 | 10.6 |
| JZJ5 | 2,721,449 | 245,530 | 105,284 | 11.084 |
| LH10 | 2,853,823 | 223,797 | 107,353 | 12.752 |
| LH11 | 3,147,666 | 231,837 | 117,723 | 13.577 |
| LH12 | 1,618,123 | 134,308 | 55,888 | 12.048 |
| LH13 | 2,442,371 | 204,132 | 93,527 | 11.965 |
| LH14 | 2,376,121 | 198,863 | 83,566 | 11.949 |
| LH15 | 1,264,552 | 111,129 | 40,829 | 11.379 |
| LH16 | 1,123,804 | 103,196 | 36,387 | 10.89 |
| LH2 | 2,210,335 | 198,430 | 74,718 | 11.139 |
| LH3 | 2,215,435 | 201,368 | 73,763 | 11.002 |
| LH4 | 2,256,329 | 199,183 | 82,154 | 11.328 |
| LH5 | 1,222,908 | 112,290 | 42,858 | 10.891 |
| LH6 | 1,536,356 | 128,925 | 51,646 | 11.917 |
| LH7 | 2,493,012 | 196,806 | 88,222 | 12.667 |
| LH8 | 2,070,624 | 170,511 | 70,881 | 12.144 |
| LH9 | 2,922,169 | 226,102 | 110,671 | 12.924 |
| LN1 | 2,718,255 | 227,717 | 107,695 | 11.937 |
| LN10 | 2,410,938 | 215,018 | 91,445 | 11.213 |
| LN11 | 1,511,540 | 149,439 | 45,615 | 10.115 |
| LN12 | 2,671,201 | 230,846 | 105,036 | 11.571 |
| LN13 | 2,963,082 | 243,528 | 115,819 | 12.167 |
| LN14 | 2,686,782 | 227,311 | 102,632 | 11.82 |
| LN15 | 2,699,142 | 230,635 | 109,444 | 11.703 |
| LN2 | 2,405,746 | 209,855 | 91,840 | 11.464 |
| LN3 | 3,018,467 | 250,543 | 124,309 | 12.048 |
| LN4 | 3,008,397 | 251,774 | 124,652 | 11.949 |
| LN5 | 2,689,614 | 233,693 | 107,652 | 11.509 |
| LN6 | 1,568,183 | 165,619 | 55,564 | 9.469 |
| LN7 | 2,799,139 | 231,487 | 105,558 | 12.092 |
| LN8 | 2,644,938 | 229,232 | 100,331 | 11.538 |
| LN9 | 2,704,219 | 229,819 | 104,062 | 11.767 |
| LS1 | 2,015,436 | 187,347 | 62,496 | 10.758 |
| LS2 | 2,143,541 | 192,772 | 69,777 | 11.12 |
| LS3 | 2,103,673 | 197,148 | 69,375 | 10.671 |
| LS4 | 1,787,748 | 169,177 | 52,411 | 10.567 |
| MS1 | 2,925,987 | 250,221 | 118,816 | 11.694 |
| MS10 | 3,124,406 | 252,282 | 126,789 | 12.385 |
| MS11 | 2,775,766 | 236,157 | 102,786 | 11.754 |
| MS12 | 3,028,623 | 241,900 | 111,824 | 12.52 |
| MS13 | 2,700,184 | 231,954 | 103,497 | 11.641 |
| MS14 | 2,913,398 | 241,911 | 111,227 | 12.043 |
| MS15 | 3,487,877 | 250,965 | 125,544 | 13.898 |
| MS2 | 2,586,988 | 239,399 | 111,312 | 10.806 |
| MS4 | 1,288,012 | 134,887 | 48,191 | 9.549 |
| MS5 | 2,190,383 | 213,558 | 85,191 | 10.257 |
| MS6 | 2,923,702 | 251,070 | 120,112 | 11.645 |
| MS7 | 2,705,647 | 247,985 | 117,144 | 10.911 |
| MS8 | 2,784,736 | 241,625 | 107,843 | 11.525 |
| MS9 | 2,747,492 | 231,490 | 102,601 | 11.869 |
| MX1 | 2,131,426 | 200,131 | 84,993 | 10.65 |
| MX10 | 1,882,681 | 176,788 | 67,806 | 10.649 |
| MX11 | 1,438,371 | 144,576 | 44,225 | 9.949 |
| MX12 | 1,403,700 | 141,633 | 44,150 | 9.911 |
| MX13 | 1,906,130 | 182,613 | 67,430 | 10.438 |
| MX14 | 1,655,778 | 160,838 | 53,165 | 10.295 |
| MX15 | 1,882,244 | 175,544 | 67,073 | 10.722 |
| MX2 | 1,546,130 | 156,133 | 50,118 | 9.903 |
| MX3 | 1,622,858 | 165,585 | 63,745 | 9.801 |
| MX4 | 1,558,447 | 160,990 | 62,039 | 9.68 |
| MX5 | 1,693,336 | 166,981 | 70,002 | 10.141 |
| MX6 | 2,231,334 | 197,607 | 82,675 | 11.292 |
| MX7 | 2,263,734 | 198,679 | 81,172 | 11.394 |
| MX8 | 1,720,849 | 167,827 | 57,354 | 10.254 |
| MX9 | 1,715,295 | 162,942 | 54,248 | 10.527 |
| SC1 | 1,229,106 | 107,897 | 40,759 | 11.391 |
| SC10 | 990,553 | 90,194 | 31,228 | 10.982 |
| SC11 | 907,591 | 82,669 | 26,978 | 10.979 |
| SC12 | 1,368,665 | 117,913 | 46,546 | 11.607 |
| SC13 | 1,073,629 | 98,444 | 33,472 | 10.906 |
| SC14 | 952,107 | 87,894 | 26,059 | 10.832 |
| SC15 | 1,402,891 | 124,018 | 47,424 | 11.312 |
| SC2 | 1,806,159 | 159,686 | 66,266 | 11.311 |
| SC3 | 1,236,860 | 112,017 | 40,338 | 11.042 |
| SC4 | 1,040,757 | 96,617 | 32,605 | 10.772 |
| SC5 | 1,001,077 | 92,844 | 29,777 | 10.782 |
| SC6 | 1,307,569 | 120,320 | 43,131 | 10.867 |
| SC7 | 980,507 | 94,012 | 27,579 | 10.43 |
| SC8 | 1,218,750 | 110,896 | 39,680 | 10.99 |
| SC9 | 1,520,732 | 133,650 | 52,154 | 11.378 |
| SN1 | 2,008,100 | 198,592 | 70,040 | 10.112 |
| SN10 | 1,648,419 | 176,609 | 59,201 | 9.334 |
| SN11 | 2,197,031 | 214,973 | 80,922 | 10.22 |
| SN12 | 2,125,555 | 205,757 | 73,585 | 10.33 |
| SN13 | 1,230,347 | 139,693 | 45,195 | 8.808 |
| SN14 | 2,073,756 | 210,694 | 82,701 | 9.843 |
| SN2 | 1,957,683 | 187,773 | 62,532 | 10.426 |
| SN3 | 2,083,693 | 202,444 | 71,051 | 10.293 |
| SN4 | 2,069,678 | 203,398 | 70,929 | 10.176 |
| SN5 | 2,197,624 | 207,114 | 75,948 | 10.611 |
| SN6 | 1,759,358 | 175,635 | 54,786 | 10.017 |
| SN7 | 1,351,344 | 142,407 | 44,723 | 9.489 |
| SN8 | 2,257,551 | 214,695 | 86,635 | 10.515 |
| SN9 | 2,110,200 | 202,082 | 72,398 | 10.442 |
| ST1 | 918,238 | 94,016 | 26,264 | 9.767 |
| ST10 | 367,255 | 41,678 | 9,550 | 8.812 |
| ST11 | 719,151 | 73,915 | 18,947 | 9.729 |
| ST12 | 534,900 | 53,927 | 11,999 | 9.919 |
| ST13 | 620,996 | 66,555 | 16,979 | 9.331 |
| ST14 | 664,075 | 68,620 | 16,454 | 9.678 |
| ST15 | 712,558 | 71,975 | 17,512 | 9.9 |
| ST2 | 1,895,160 | 165,765 | 61,538 | 11.433 |
| ST3 | 2,111,750 | 182,967 | 71,001 | 11.542 |
| ST4 | 2,142,138 | 195,799 | 79,140 | 10.94 |
| ST5 | 552,057 | 60,717 | 14,835 | 9.092 |
| ST6 | 1,022,968 | 104,407 | 27,716 | 9.798 |
| ST7 | 803,548 | 84,463 | 19,354 | 9.514 |
| ST8 | 817,805 | 81,724 | 22,179 | 10.007 |
| ST9 | 892,748 | 88,876 | 25,776 | 10.045 |
| SZ1 | 1,830,471 | 158,515 | 65,814 | 11.548 |
| SZ10 | 993,668 | 104,609 | 29,636 | 9.499 |
| SZ11 | 1,765,996 | 163,498 | 66,339 | 10.801 |
| SZ12 | 1,985,442 | 170,046 | 78,912 | 11.676 |
| SZ13 | 3,356,349 | 236,630 | 116,558 | 14.184 |
| SZ14 | 1,502,496 | 133,075 | 57,044 | 11.291 |
| SZ15 | 1,865,132 | 157,878 | 68,122 | 11.814 |
| SZ2 | 1,758,543 | 159,516 | 63,082 | 11.024 |
| SZ3 | 2,676,773 | 207,161 | 94,409 | 12.921 |
| SZ4 | 3,161,698 | 229,213 | 111,943 | 13.794 |
| SZ5 | 1,940,971 | 174,698 | 76,033 | 11.11 |
| SZ6 | 1,825,097 | 164,311 | 68,403 | 11.108 |
| SZ7 | 2,908,572 | 222,319 | 103,282 | 13.083 |
| SZ8 | 2,864,727 | 220,276 | 99,601 | 13.005 |
| SZ9 | 2,337,910 | 195,294 | 88,354 | 11.971 |
| TB10 | 4,644,558 | 292,050 | 153,985 | 15.903 |
| TB11 | 1,064,036 | 106,280 | 30,346 | 10.012 |
| TB14 | 638,212 | 62,940 | 15,407 | 10.14 |
| TB15 | 487,936 | 46,158 | 10,097 | 10.571 |
| TB16 | 925,087 | 88,368 | 23,975 | 10.469 |
| TB17 | 870,093 | 85,626 | 23,096 | 10.162 |
| TB18 | 900,628 | 87,973 | 23,213 | 10.238 |
| TB2 | 3,045,180 | 233,352 | 103,341 | 13.05 |
| TB3 | 2,967,164 | 234,738 | 101,838 | 12.64 |
| TB4 | 2,905,094 | 230,837 | 99,485 | 12.585 |
| TB5 | 3,221,417 | 244,458 | 110,497 | 13.178 |
| TB6 | 2,826,398 | 233,302 | 99,623 | 12.115 |
| TB7 | 2,652,874 | 213,423 | 85,354 | 12.43 |
| TB8 | 2,618,153 | 216,257 | 88,600 | 12.107 |
| TB9 | 2,631,081 | 212,657 | 84,396 | 12.372 |
| TMS1 | 2,887,177 | 228,136 | 106,506 | 12.656 |
| TMS10 | 2,281,575 | 200,154 | 80,552 | 11.399 |
| TMS11 | 2,238,346 | 201,186 | 76,787 | 11.126 |
| TMS12 | 1,847,278 | 173,445 | 57,808 | 10.651 |
| TMS13 | 2,316,791 | 211,042 | 81,757 | 10.978 |
| TMS14 | 2,617,068 | 224,259 | 98,053 | 11.67 |
| TMS15 | 3,344,193 | 249,350 | 118,460 | 13.412 |
| TMS2 | 3,008,175 | 244,921 | 114,522 | 12.282 |
| TMS3 | 2,001,400 | 185,192 | 69,925 | 10.807 |
| TMS4 | 2,567,621 | 215,320 | 92,866 | 11.925 |
| TMS5 | 2,501,138 | 217,083 | 91,535 | 11.522 |
| TMS6 | 1,294,854 | 128,928 | 43,985 | 10.043 |
| TMS7 | 1,956,439 | 181,480 | 63,453 | 10.78 |
| TMS8 | 1,773,400 | 175,820 | 62,931 | 10.086 |
| TMS9 | 1,306,084 | 135,898 | 42,186 | 9.611 |
| TZ1 | 2,644,730 | 217,628 | 95,238 | 12.153 |
| TZ10 | 3,029,582 | 239,599 | 105,226 | 12.644 |
| TZ11 | 2,683,873 | 219,529 | 98,563 | 12.226 |
| TZ12 | 2,961,392 | 241,054 | 107,320 | 12.285 |
| TZ13 | 1,934,368 | 173,030 | 70,339 | 11.179 |
| TZ14 | 1,988,119 | 178,180 | 70,337 | 11.158 |
| TZ15 | 2,885,594 | 218,546 | 92,370 | 13.204 |
| TZ16 | 2,856,808 | 233,182 | 100,149 | 12.251 |
| TZ2 | 2,739,020 | 220,508 | 97,419 | 12.421 |
| TZ4 | 2,437,638 | 205,024 | 81,564 | 11.89 |
| TZ5 | 3,370,109 | 250,645 | 111,801 | 13.446 |
| TZ6 | 2,912,189 | 237,955 | 101,861 | 12.238 |
| TZ7 | 2,936,620 | 236,758 | 103,118 | 12.403 |
| TZ8 | 2,056,243 | 177,519 | 70,750 | 11.583 |
| TZ9 | 1,912,507 | 172,166 | 65,078 | 11.109 |
| XE1 | 1,801,027 | 153,175 | 63,648 | 11.758 |
| XE10 | 2,369,386 | 197,504 | 91,698 | 11.997 |
| XE11 | 1,107,181 | 116,094 | 40,151 | 9.537 |
| XE12 | 1,921,508 | 169,654 | 70,577 | 11.326 |
| XE13 | 1,751,435 | 150,960 | 62,403 | 11.602 |
| XE14 | 1,608,674 | 141,292 | 56,390 | 11.385 |
| XE15 | 1,928,256 | 164,257 | 71,753 | 11.739 |
| XE2 | 1,992,724 | 171,107 | 75,053 | 11.646 |
| XE3 | 3,323,957 | 262,224 | 124,412 | 12.676 |
| XE4 | 1,892,129 | 172,478 | 71,292 | 10.97 |
| XE5 | 2,354,670 | 190,886 | 88,818 | 12.335 |
| XE6 | 2,422,679 | 206,774 | 94,855 | 11.717 |
| XE7 | 2,426,407 | 192,712 | 92,970 | 12.591 |
| XE8 | 2,154,799 | 183,595 | 81,360 | 11.737 |
| XE9 | 2,507,447 | 205,360 | 95,265 | 12.21 |

**TABLE S6.** A statistical tabulation detailing the comparison between the reads and the reference genome of 348 samples of *C. kousa* sourced from 25 natural populations.

| Sample | Clean reads | Mapped reads | Mapping rate | Average depth | Coverage 1× | Coverage 4× |
| --- | --- | --- | --- | --- | --- | --- |
| AHS1 | 1,936,562 | 1,587,614 | 81.98% | 12.89 | 19.55% | 10.42% |
| AHS10 | 3,877,096 | 2,907,497 | 74.99% | 15.5 | 24.05% | 16.74% |
| AHS11 | 2,618,578 | 2,104,097 | 80.35% | 12.97 | 22.64% | 14.16% |
| AHS12 | 3,212,874 | 2,838,809 | 88.36% | 11.23 | 29.99% | 22.80% |
| AHS13 | 2,531,916 | 2,055,718 | 81.19% | 13.93 | 20.47% | 13.02% |
| AHS14 | 2,624,704 | 2,090,190 | 79.64% | 13.99 | 20.79% | 13.10% |
| AHS15 | 2,454,820 | 2,002,067 | 81.56% | 13.9 | 20.31% | 12.62% |
| AHS2 | 3,204,232 | 2,535,655 | 79.13% | 14.77 | 22.70% | 15.32% |
| AHS3 | 2,246,056 | 1,827,715 | 81.37% | 13.48 | 20.17% | 11.73% |
| AHS4 | 3,460,714 | 2,722,498 | 78.67% | 15.44 | 22.94% | 15.85% |
| AHS5 | 1,814,904 | 1,457,452 | 80.30% | 12.3 | 19.21% | 9.87% |
| AHS6 | 2,041,998 | 1,637,600 | 80.20% | 13.06 | 19.67% | 10.61% |
| AHS7 | 2,280,414 | 1,825,338 | 80.04% | 14.32 | 19.55% | 10.90% |
| AHS8 | 4,512,818 | 3,542,783 | 78.50% | 17.81 | 24.31% | 18.12% |
| AHS9 | 3,288,944 | 2,609,424 | 79.34% | 16.29 | 21.31% | 14.23% |
| AK1 | 2,990,970 | 2,266,402 | 75.77% | 14.57 | 21.17% | 13.65% |
| AK10 | 1,947,292 | 1,546,090 | 79.40% | 13.19 | 18.03% | 9.93% |
| AK11 | 1,803,280 | 1,416,947 | 78.58% | 12.96 | 17.60% | 9.13% |
| AK12 | 2,938,722 | 2,250,309 | 76.57% | 14.59 | 20.88% | 13.57% |
| AK13 | 1,888,802 | 1,510,127 | 79.95% | 13.02 | 18.03% | 9.82% |
| AK14 | 1,851,078 | 1,471,502 | 79.49% | 12.98 | 17.87% | 9.51% |
| AK15 | 2,266,456 | 1,771,865 | 78.18% | 13.71 | 19.14% | 11.07% |
| AK2 | 1,946,952 | 1,528,351 | 78.50% | 13.14 | 18.39% | 9.77% |
| AK3 | 2,103,730 | 1,597,769 | 75.95% | 12.47 | 19.05% | 10.79% |
| AK4 | 2,298,694 | 1,782,089 | 77.53% | 13.1 | 19.39% | 11.76% |
| AK5 | 1,638,800 | 1,208,063 | 73.72% | 11.62 | 17.66% | 8.27% |
| AK6 | 3,673,058 | 2,781,607 | 75.73% | 16.59 | 21.81% | 14.99% |
| AK7 | 2,013,764 | 1,577,788 | 78.35% | 12.99 | 18.51% | 10.27% |
| AK8 | 1,739,244 | 1,353,849 | 77.84% | 12.98 | 17.27% | 8.62% |
| AK9 | 1,643,972 | 1,288,946 | 78.40% | 12.3 | 17.34% | 8.60% |
| BHS1 | 2,618,602 | 1,829,297 | 69.86% | 12.66 | 21.24% | 12.02% |
| BHS12 | 2,420,148 | 1,762,827 | 72.84% | 12.45 | 20.78% | 11.88% |
| BHS13 | 2,608,146 | 1,873,018 | 71.81% | 12.84 | 21.17% | 12.36% |
| BHS14 | 2,925,062 | 2,152,011 | 73.57% | 13.54 | 21.48% | 13.79% |
| BHS15 | 2,776,336 | 2,026,235 | 72.98% | 13.13 | 21.57% | 13.20% |
| BHS2 | 2,848,752 | 2,090,980 | 73.40% | 13.35 | 21.68% | 13.45% |
| BHS3 | 2,602,274 | 1,905,725 | 73.23% | 12.94 | 21.25% | 12.43% |
| BHS4 | 2,374,570 | 1,688,830 | 71.12% | 12.09 | 20.96% | 11.51% |
| BHS5 | 2,280,810 | 1,637,791 | 71.81% | 11.83 | 20.85% | 11.40% |
| BHS6 | 2,386,608 | 1,608,867 | 67.41% | 12.2 | 20.68% | 10.64% |
| BHS7 | 3,004,472 | 2,187,234 | 72.80% | 13.63 | 21.91% | 13.80% |
| ES1 | 2,768,266 | 2,037,332 | 73.60% | 13.86 | 21.28% | 12.58% |
| ES10 | 2,971,826 | 2,232,242 | 75.11% | 14.46 | 21.60% | 13.37% |
| ES11 | 1,239,368 | 934,650 | 75.41% | 10.56 | 17.13% | 6.55% |
| ES12 | 957,104 | 741,067 | 77.43% | 10.16 | 15.15% | 5.19% |
| ES13 | 1,267,984 | 971,227 | 76.60% | 10.63 | 17.35% | 6.84% |
| ES14 | 1,337,342 | 985,124 | 73.66% | 10.72 | 17.79% | 6.79% |
| ES15 | 1,472,528 | 1,095,438 | 74.39% | 10.82 | 18.42% | 7.71% |
| ES16 | 1,190,844 | 896,817 | 75.31% | 9.85 | 17.43% | 6.63% |
| ES2 | 2,835,896 | 2,042,716 | 72.03% | 14.02 | 21.51% | 12.39% |
| ES3 | 3,241,806 | 2,369,468 | 73.09% | 14.85 | 22.14% | 13.86% |
| ES4 | 3,012,752 | 2,209,994 | 73.35% | 14.24 | 21.85% | 13.40% |
| ES5 | 1,345,354 | 1,019,847 | 75.81% | 10.57 | 17.90% | 7.26% |
| ES7 | 1,184,818 | 986,353 | 83.25% | 9.75 | 19.05% | 7.40% |
| ES8 | 1,372,614 | 1,073,428 | 78.20% | 11.14 | 17.97% | 7.41% |
| ES9 | 1,246,278 | 955,300 | 76.65% | 10.56 | 17.53% | 6.71% |
| FN1 | 1,238,796 | 899,385 | 72.60% | 10.55 | 17.07% | 6.14% |
| FN10 | 1,692,450 | 1,151,126 | 68.02% | 11.02 | 18.92% | 7.80% |
| FN11 | 1,818,612 | 1,285,938 | 70.71% | 11.08 | 19.55% | 9.00% |
| FN12 | 2,061,522 | 1,464,407 | 71.04% | 11.57 | 20.32% | 10.12% |
| FN13 | 2,491,578 | 1,868,112 | 74.98% | 12.24 | 21.32% | 13.03% |
| FN14 | 1,859,058 | 1,296,984 | 69.77% | 11.06 | 19.86% | 9.08% |
| FN15 | 2,996,638 | 2,055,281 | 68.59% | 14.14 | 21.28% | 12.29% |
| FN2 | 1,187,744 | 851,026 | 71.65% | 10.22 | 16.74% | 5.88% |
| FN3 | 1,473,698 | 1,103,050 | 74.85% | 10.79 | 18.09% | 7.91% |
| FN4 | 1,222,242 | 916,881 | 75.02% | 10.26 | 17.15% | 6.55% |
| FN5 | 1,256,350 | 935,039 | 74.43% | 10.65 | 17.09% | 6.46% |
| FN6 | 1,465,642 | 1,093,930 | 74.64% | 10.98 | 17.87% | 7.65% |
| FN7 | 1,339,238 | 1,007,766 | 75.25% | 10.7 | 17.62% | 7.07% |
| FN8 | 1,406,876 | 1,080,852 | 76.83% | 10.69 | 17.66% | 7.86% |
| FN9 | 2,078,904 | 1,499,768 | 72.14% | 11.02 | 20.82% | 11.05% |
| H4 | 2,349,692 | 1,728,966 | 73.58% | 12.22 | 21.58% | 11.72% |
| H5 | 2,654,370 | 1,844,612 | 69.49% | 12.93 | 21.33% | 11.74% |
| H6 | 2,904,662 | 2,045,044 | 70.41% | 13.32 | 21.96% | 12.91% |
| H7 | 2,992,978 | 2,222,046 | 74.24% | 14.61 | 21.29% | 13.25% |
| H9 | 4,480,110 | 3,322,623 | 74.16% | 18.13 | 23.12% | 16.52% |
| HA1 | 4,021,172 | 2,982,424 | 74.17% | 17.79 | 21.87% | 14.99% |
| HA11 | 5,327,182 | 4,045,758 | 75.95% | 21.19 | 22.85% | 17.16% |
| HA12 | 3,988,068 | 2,921,613 | 73.26% | 18.27 | 20.82% | 14.12% |
| HA13 | 3,996,808 | 2,902,618 | 72.62% | 17.17 | 21.74% | 15.00% |
| HA14 | 4,029,544 | 2,863,751 | 71.07% | 17.16 | 22.01% | 14.73% |
| HA15 | 3,384,316 | 2,597,249 | 76.74% | 15.87 | 21.32% | 14.59% |
| HA16 | 2,684,986 | 2,014,132 | 75.01% | 14.53 | 20.31% | 11.90% |
| HA2 | 3,812,252 | 2,755,791 | 72.29% | 17.06 | 21.68% | 14.08% |
| HA3 | 3,602,192 | 2,609,183 | 72.43% | 16.42 | 21.52% | 14.00% |
| HA4 | 5,176,338 | 3,823,396 | 73.86% | 20.3 | 22.89% | 16.97% |
| HA5 | 3,851,002 | 2,448,981 | 63.59% | 15.89 | 21.89% | 13.05% |
| HA6 | 5,078,338 | 3,718,115 | 73.22% | 19.94 | 22.85% | 16.85% |
| HA7 | 3,054,338 | 2,256,775 | 73.89% | 14.93 | 20.92% | 13.16% |
| HA8 | 4,704,570 | 3,522,881 | 74.88% | 19.39 | 22.37% | 16.34% |
| HA9 | 4,310,742 | 3,124,522 | 72.48% | 17.42 | 22.51% | 16.06% |
| HG12 | 3,220,146 | 2,438,530 | 75.73% | 15.37 | 21.85% | 13.87% |
| HG13 | 2,937,736 | 2,138,261 | 72.79% | 14.21 | 21.63% | 12.94% |
| HG14 | 4,504,530 | 3,242,013 | 71.97% | 17.94 | 23.52% | 16.08% |
| HG2 | 4,423,234 | 3,151,544 | 71.25% | 17.42 | 23.51% | 16.03% |
| HG3 | 4,583,616 | 3,461,174 | 75.51% | 18.79 | 23.14% | 16.52% |
| HG4 | 3,746,346 | 2,757,904 | 73.62% | 16.08 | 22.53% | 15.23% |
| HG5 | 2,629,152 | 1,889,473 | 71.87% | 13.27 | 21.38% | 11.92% |
| HG6 | 2,708,456 | 1,901,974 | 70.22% | 12.81 | 21.62% | 12.28% |
| HG7 | 2,925,818 | 2,078,832 | 71.05% | 13.74 | 21.40% | 12.91% |
| HG9 | 2,886,786 | 2,174,419 | 75.32% | 14.23 | 21.49% | 13.24% |
| HS1 | 3,477,134 | 2,635,624 | 75.80% | 14.98 | 21.85% | 15.78% |
| HS11 | 2,638,760 | 1,905,136 | 72.20% | 12.51 | 21.53% | 12.88% |
| HS12 | 3,267,426 | 2,419,254 | 74.04% | 14.41 | 21.91% | 14.78% |
| HS13 | 3,065,780 | 2,267,764 | 73.97% | 13.71 | 21.72% | 14.45% |
| HS14 | 2,690,252 | 1,981,707 | 73.66% | 12.58 | 21.59% | 13.52% |
| HS15 | 3,080,200 | 2,296,039 | 74.54% | 13.65 | 22.04% | 14.73% |
| HS16 | 2,977,934 | 2,214,362 | 74.36% | 13.27 | 22.11% | 14.50% |
| HS17 | 3,118,114 | 2,320,806 | 74.43% | 13.69 | 22.04% | 14.87% |
| HS2 | 2,972,948 | 2,255,486 | 75.87% | 13.63 | 21.90% | 14.52% |
| HS3 | 3,051,426 | 2,260,127 | 74.07% | 13.51 | 21.93% | 14.67% |
| HS4 | 3,206,162 | 2,419,982 | 75.48% | 14.18 | 22.17% | 15.03% |
| HS5 | 3,211,658 | 2,420,346 | 75.36% | 13.86 | 22.23% | 15.45% |
| HS6 | 3,128,436 | 2,121,328 | 67.81% | 13.55 | 21.31% | 13.54% |
| HS7 | 3,094,530 | 2,228,505 | 72.01% | 14.61 | 21.67% | 13.10% |
| HS8 | 2,653,194 | 1,937,124 | 73.01% | 12.37 | 21.67% | 13.34% |
| JG1 | 2,454,318 | 1,821,196 | 74.20% | 11.86 | 21.58% | 13.05% |
| JG11 | 3,509,108 | 2,456,069 | 69.99% | 15.09 | 22.70% | 14.05% |
| JG12 | 2,088,486 | 1,493,368 | 71.50% | 10.99 | 21.03% | 10.95% |
| JG13 | 2,324,338 | 1,671,361 | 71.91% | 11.42 | 21.66% | 12.06% |
| JG14 | 2,593,578 | 1,940,837 | 74.83% | 12.39 | 21.72% | 13.43% |
| JG15 | 2,605,510 | 1,951,700 | 74.91% | 12.23 | 21.63% | 13.74% |
| JG17 | 2,726,784 | 1,995,569 | 73.18% | 12.26 | 21.98% | 14.03% |
| JG18 | 1,715,836 | 1,184,752 | 69.05% | 10.56 | 19.38% | 8.49% |
| JG2 | 2,250,554 | 1,676,428 | 74.49% | 11.72 | 21.04% | 11.98% |
| JG3 | 2,074,274 | 1,549,772 | 74.71% | 11.55 | 20.76% | 10.97% |
| JG5 | 2,599,654 | 1,960,113 | 75.40% | 12.55 | 21.53% | 13.45% |
| JG6 | 2,407,414 | 1,785,195 | 74.15% | 12.01 | 21.41% | 12.56% |
| JG7 | 2,409,494 | 1,811,482 | 75.18% | 12.12 | 21.45% | 12.66% |
| JG8 | 2,076,864 | 1,525,833 | 73.47% | 11.86 | 19.86% | 10.63% |
| JG9 | 1,843,874 | 1,244,636 | 67.50% | 10.79 | 19.52% | 8.89% |
| JHS1 | 2,786,282 | 2,140,468 | 76.82% | 12.95 | 22.09% | 14.41% |
| JHS10 | 2,630,448 | 1,982,305 | 75.36% | 12.29 | 22.12% | 13.86% |
| JHS11 | 2,084,170 | 1,521,806 | 73.02% | 11.39 | 21.09% | 10.77% |
| JHS12 | 2,694,874 | 1,985,739 | 73.69% | 12.53 | 21.83% | 13.56% |
| JHS13 | 2,919,562 | 2,175,121 | 74.50% | 12.65 | 22.57% | 14.98% |
| JHS14 | 2,375,060 | 1,716,161 | 72.26% | 11.59 | 21.73% | 12.28% |
| JHS15 | 3,048,824 | 2,323,819 | 76.22% | 13.48 | 22.50% | 15.19% |
| JHS2 | 3,533,368 | 2,561,121 | 72.48% | 15.14 | 23.08% | 14.72% |
| JHS3 | 2,317,068 | 1,742,349 | 75.20% | 11.82 | 21.43% | 12.38% |
| JHS4 | 3,345,150 | 2,582,715 | 77.21% | 15.65 | 22.30% | 14.64% |
| JHS5 | 2,602,026 | 1,961,215 | 75.37% | 12.22 | 22.09% | 13.81% |
| JHS6 | 2,853,132 | 2,156,427 | 75.58% | 13.87 | 22.03% | 13.39% |
| JHS7 | 2,502,762 | 1,926,446 | 76.97% | 13.37 | 21.38% | 12.25% |
| JHS8 | 2,360,670 | 1,771,080 | 75.02% | 11.65 | 21.80% | 12.83% |
| JHS9 | 2,736,824 | 2,007,126 | 73.34% | 12.24 | 22.37% | 14.05% |
| JLS1 | 2,049,448 | 1,486,240 | 72.52% | 11.17 | 20.21% | 10.90% |
| JLS10 | 2,323,750 | 1,655,701 | 71.25% | 12.15 | 20.19% | 11.34% |
| JLS11 | 3,840,070 | 2,842,527 | 74.02% | 16.11 | 22.74% | 15.71% |
| JLS12 | 2,158,486 | 1,550,555 | 71.84% | 11.07 | 21.03% | 11.43% |
| JLS13 | 2,248,240 | 1,642,655 | 73.06% | 11.4 | 21.20% | 11.93% |
| JLS14 | 2,474,320 | 1,819,521 | 73.54% | 12.3 | 21.16% | 12.51% |
| JLS15 | 2,399,466 | 1,787,526 | 74.50% | 11.61 | 21.42% | 13.07% |
| JLS2 | 2,641,370 | 1,938,116 | 73.38% | 12.35 | 21.55% | 13.52% |
| JLS3 | 2,584,370 | 1,929,293 | 74.65% | 12.04 | 21.78% | 13.79% |
| JLS4 | 1,319,198 | 931,908 | 70.64% | 9.71 | 18.32% | 6.88% |
| JLS5 | 3,833,930 | 2,703,983 | 70.53% | 15.52 | 22.70% | 15.45% |
| JLS6 | 3,618,578 | 2,557,925 | 70.69% | 14.71 | 22.79% | 15.26% |
| JLS7 | 2,255,384 | 1,691,368 | 74.99% | 11.56 | 20.88% | 12.35% |
| JLS8 | 3,522,200 | 2,469,232 | 70.10% | 15 | 22.29% | 14.30% |
| JLS9 | 2,521,114 | 1,921,643 | 76.22% | 12.37 | 21.44% | 13.38% |
| JZJ1 | 3,265,018 | 2,262,220 | 69.29% | 12.79 | 22.58% | 15.54% |
| JZJ2 | 2,718,232 | 1,938,471 | 71.31% | 12.47 | 21.12% | 13.41% |
| JZJ3 | 3,522,132 | 2,531,557 | 71.88% | 13.99 | 22.65% | 16.05% |
| JZJ4 | 3,382,454 | 2,404,695 | 71.09% | 13.31 | 22.79% | 15.96% |
| JZJ5 | 3,666,238 | 2,612,304 | 71.25% | 14.07 | 22.98% | 16.52% |
| LH10 | 3,929,958 | 2,874,939 | 73.15% | 16.13 | 22.85% | 15.85% |
| LH11 | 4,036,418 | 3,063,028 | 75.88% | 17.08 | 22.50% | 16.13% |
| LH12 | 2,158,680 | 1,698,736 | 78.69% | 13.74 | 18.75% | 10.55% |
| LH13 | 3,290,182 | 2,420,986 | 73.58% | 14.83 | 21.91% | 14.40% |
| LH14 | 3,435,502 | 2,486,211 | 72.37% | 14.66 | 22.68% | 14.80% |
| LH15 | 1,781,030 | 1,390,969 | 78.10% | 12.67 | 18.01% | 9.11% |
| LH16 | 1,617,906 | 1,262,549 | 78.04% | 12.02 | 17.48% | 8.58% |
| LH2 | 2,973,878 | 2,236,291 | 75.20% | 13.77 | 21.63% | 14.16% |
| LH3 | 3,085,252 | 2,268,019 | 73.51% | 13.74 | 22.05% | 14.33% |
| LH4 | 3,006,412 | 2,226,267 | 74.05% | 14.16 | 21.33% | 13.68% |
| LH5 | 1,692,326 | 1,321,045 | 78.06% | 12.3 | 17.50% | 8.87% |
| LH6 | 2,101,486 | 1,642,500 | 78.16% | 13.56 | 18.62% | 10.25% |
| LH7 | 3,344,826 | 2,518,900 | 75.31% | 15.37 | 21.81% | 14.49% |
| LH8 | 3,045,888 | 2,230,542 | 73.23% | 14.55 | 21.82% | 13.24% |
| LH9 | 3,797,704 | 2,866,757 | 75.49% | 16.16 | 22.62% | 15.90% |
| LN1 | 3,409,950 | 2,573,891 | 75.48% | 15.06 | 21.11% | 15.27% |
| LN10 | 3,131,448 | 2,348,639 | 75.00% | 14.1 | 21.18% | 14.66% |
| LN11 | 2,350,214 | 1,673,866 | 71.22% | 12.21 | 20.09% | 11.38% |
| LN12 | 3,337,588 | 2,475,314 | 74.16% | 14.53 | 21.19% | 15.19% |
| LN13 | 3,693,852 | 2,765,701 | 74.87% | 15.55 | 21.63% | 15.96% |
| LN14 | 3,346,978 | 2,532,634 | 75.67% | 14.8 | 21.38% | 15.27% |
| LN15 | 3,369,286 | 2,496,664 | 74.10% | 14.82 | 21.22% | 15.05% |
| LN2 | 2,985,426 | 2,277,121 | 76.27% | 14.29 | 20.54% | 14.16% |
| LN3 | 3,664,088 | 2,779,447 | 75.86% | 15.5 | 21.66% | 16.15% |
| LN4 | 3,595,600 | 2,747,712 | 76.42% | 15.29 | 21.63% | 16.21% |
| LN5 | 3,294,306 | 2,508,617 | 76.15% | 14.45 | 21.38% | 15.50% |
| LN6 | 2,076,812 | 1,573,733 | 75.78% | 11.31 | 19.78% | 11.76% |
| LN7 | 3,509,420 | 2,644,077 | 75.34% | 15.18 | 21.64% | 15.56% |
| LN8 | 3,444,772 | 2,536,666 | 73.64% | 14.58 | 21.71% | 15.43% |
| LN9 | 3,444,316 | 2,566,143 | 74.50% | 14.9 | 21.50% | 15.33% |
| LS1 | 2,819,540 | 2,054,098 | 72.85% | 13.29 | 21.18% | 13.33% |
| LS2 | 2,954,026 | 2,159,054 | 73.09% | 13.78 | 21.35% | 13.60% |
| LS3 | 2,860,972 | 2,101,431 | 73.45% | 13.21 | 21.35% | 13.83% |
| LS4 | 2,622,568 | 1,888,904 | 72.02% | 12.85 | 21.07% | 12.47% |
| MS1 | 3,693,498 | 2,721,312 | 73.68% | 14.73 | 22.64% | 16.64% |
| MS10 | 3,934,866 | 2,905,819 | 73.85% | 15.66 | 22.58% | 16.78% |
| MS11 | 3,598,358 | 2,692,370 | 74.82% | 14.66 | 22.89% | 16.41% |
| MS12 | 3,845,810 | 2,899,388 | 75.39% | 15.6 | 22.80% | 16.72% |
| MS13 | 3,618,710 | 2,604,637 | 71.98% | 14.45 | 22.65% | 16.09% |
| MS14 | 3,769,608 | 2,782,600 | 73.82% | 14.93 | 22.88% | 16.76% |
| MS15 | 4,388,584 | 3,358,098 | 76.52% | 17.73 | 23.26% | 17.14% |
| MS2 | 3,353,508 | 2,455,620 | 73.23% | 14 | 22.29% | 15.60% |
| MS4 | 1,955,862 | 1,387,689 | 70.95% | 11.31 | 19.29% | 10.05% |
| MS5 | 3,131,688 | 2,211,371 | 70.61% | 12.86 | 22.34% | 15.05% |
| MS6 | 3,808,300 | 2,761,938 | 72.52% | 14.67 | 22.87% | 16.96% |
| MS7 | 3,516,224 | 2,568,518 | 73.05% | 14.16 | 22.50% | 16.21% |
| MS8 | 3,669,034 | 2,674,299 | 72.89% | 14.41 | 22.84% | 16.58% |
| MS9 | 3,549,370 | 2,630,391 | 74.11% | 14.7 | 22.55% | 16.04% |
| MX1 | 2,943,570 | 2,124,426 | 72.17% | 13.58 | 21.06% | 13.54% |
| MX10 | 2,765,048 | 1,977,570 | 71.52% | 13.33 | 20.88% | 12.57% |
| MX11 | 2,555,554 | 1,701,423 | 66.58% | 12.33 | 20.77% | 11.18% |
| MX12 | 2,266,386 | 1,589,206 | 70.12% | 11.96 | 20.12% | 10.87% |
| MX13 | 2,847,066 | 2,001,927 | 70.32% | 13 | 21.22% | 13.09% |
| MX14 | 2,593,692 | 1,821,558 | 70.23% | 12.63 | 20.78% | 12.04% |
| MX15 | 2,760,726 | 1,967,997 | 71.29% | 13.22 | 20.82% | 12.64% |
| MX2 | 2,554,070 | 1,741,823 | 68.20% | 12.3 | 20.84% | 11.66% |
| MX3 | 2,202,230 | 1,620,999 | 73.61% | 12.03 | 19.46% | 11.40% |
| MX4 | 2,365,926 | 1,632,836 | 69.01% | 12.4 | 19.31% | 10.89% |
| MX5 | 2,232,522 | 1,655,956 | 74.17% | 12.57 | 18.80% | 11.24% |
| MX6 | 2,973,012 | 2,206,709 | 74.22% | 13.93 | 21.06% | 13.84% |
| MX7 | 3,102,128 | 2,279,837 | 73.49% | 14.2 | 21.49% | 13.95% |
| MX8 | 2,552,208 | 1,823,432 | 71.45% | 12.47 | 20.85% | 12.34% |
| MX9 | 2,667,266 | 1,870,762 | 70.14% | 12.96 | 20.86% | 12.11% |
| SC1 | 1,759,488 | 1,358,452 | 77.21% | 12.71 | 17.40% | 8.84% |
| SC10 | 1,487,876 | 1,149,465 | 77.26% | 11.97 | 16.61% | 7.72% |
| SC11 | 1,409,892 | 1,084,032 | 76.89% | 11.85 | 16.35% | 7.24% |
| SC12 | 1,886,698 | 1,474,512 | 78.15% | 13.03 | 17.70% | 9.47% |
| SC13 | 1,593,402 | 1,226,805 | 76.99% | 12.05 | 17.09% | 8.26% |
| SC14 | 1,624,916 | 1,166,301 | 71.78% | 12.07 | 16.98% | 7.61% |
| SC15 | 1,913,382 | 1,489,209 | 77.83% | 12.84 | 18.09% | 9.75% |
| SC2 | 2,414,052 | 1,822,468 | 75.49% | 13.47 | 19.53% | 11.61% |
| SC3 | 1,804,224 | 1,378,872 | 76.42% | 12.39 | 18.01% | 9.15% |
| SC4 | 1,647,660 | 1,227,591 | 74.51% | 11.89 | 17.55% | 8.26% |
| SC5 | 1,612,200 | 1,197,244 | 74.26% | 11.87 | 17.40% | 8.01% |
| SC6 | 1,929,836 | 1,445,515 | 74.90% | 12.37 | 18.40% | 9.65% |
| SC7 | 1,638,500 | 1,186,565 | 72.42% | 11.67 | 17.50% | 8.01% |
| SC8 | 1,849,074 | 1,378,727 | 74.56% | 12.34 | 18.09% | 9.14% |
| SC9 | 2,050,746 | 1,598,805 | 77.96% | 13.03 | 18.41% | 10.41% |
| SN1 | 2,931,392 | 2,032,236 | 69.33% | 12.62 | 21.67% | 13.86% |
| SN10 | 2,508,864 | 1,717,691 | 68.46% | 11.72 | 20.71% | 12.38% |
| SN11 | 3,002,126 | 2,128,027 | 70.88% | 12.83 | 21.82% | 14.44% |
| SN12 | 3,096,112 | 2,146,059 | 69.31% | 13.1 | 21.90% | 14.11% |
| SN13 | 1,967,830 | 1,337,915 | 67.99% | 11.17 | 18.83% | 9.62% |
| SN14 | 2,872,582 | 2,029,012 | 70.63% | 12.64 | 21.42% | 13.87% |
| SN2 | 2,743,710 | 1,998,970 | 72.86% | 12.88 | 21.48% | 13.30% |
| SN3 | 2,783,474 | 2,055,022 | 73.83% | 12.76 | 21.54% | 13.98% |
| SN4 | 2,980,922 | 2,096,249 | 70.32% | 12.7 | 21.93% | 14.19% |
| SN5 | 2,959,394 | 2,151,613 | 72.70% | 13.15 | 21.69% | 14.29% |
| SN6 | 2,691,654 | 1,869,362 | 69.45% | 12.25 | 21.59% | 12.88% |
| SN7 | 2,045,730 | 1,427,951 | 69.80% | 11.32 | 19.53% | 10.32% |
| SN8 | 3,084,270 | 2,204,644 | 71.48% | 13.34 | 21.77% | 14.41% |
| SN9 | 2,912,544 | 2,092,628 | 71.85% | 12.87 | 21.67% | 14.10% |
| ST1 | 1,509,744 | 1,097,553 | 72.70% | 10.78 | 18.04% | 7.87% |
| ST10 | 789,332 | 538,352 | 68.20% | 9.3 | 13.29% | 3.71% |
| ST11 | 1,228,810 | 930,849 | 75.75% | 10.36 | 17.25% | 6.62% |
| ST12 | 1,100,572 | 787,186 | 71.53% | 10.3 | 16.20% | 5.27% |
| ST13 | 1,079,394 | 811,632 | 75.19% | 9.92 | 16.12% | 5.90% |
| ST14 | 1,156,954 | 874,739 | 75.61% | 10.27 | 16.84% | 6.17% |
| ST15 | 1,315,348 | 944,871 | 71.83% | 10.51 | 17.39% | 6.61% |
| ST2 | 3,118,486 | 2,128,092 | 68.24% | 14.14 | 21.79% | 12.68% |
| ST3 | 3,440,254 | 2,292,849 | 66.65% | 14.39 | 22.26% | 13.61% |
| ST4 | 3,299,516 | 2,050,271 | 62.14% | 13.68 | 21.22% | 12.85% |
| ST5 | 955,658 | 733,051 | 76.71% | 9.68 | 15.45% | 5.32% |
| ST6 | 1,557,802 | 1,189,405 | 76.35% | 10.88 | 18.54% | 8.64% |
| ST7 | 1,308,130 | 995,960 | 76.14% | 10.3 | 17.83% | 7.27% |
| ST8 | 1,370,568 | 1,010,642 | 73.74% | 10.77 | 17.52% | 7.10% |
| ST9 | 1,411,630 | 1,076,760 | 76.28% | 10.86 | 17.84% | 7.66% |
| SZ1 | 2,391,778 | 1,840,703 | 76.96% | 13.48 | 19.29% | 11.79% |
| SZ10 | 1,572,234 | 1,151,842 | 73.26% | 10.64 | 18.15% | 8.53% |
| SZ11 | 2,310,092 | 1,733,191 | 75.03% | 12.77 | 19.26% | 11.71% |
| SZ12 | 2,505,502 | 1,943,530 | 77.57% | 14.03 | 19.39% | 12.11% |
| SZ13 | 4,307,832 | 3,246,783 | 75.37% | 18.06 | 22.46% | 16.20% |
| SZ14 | 1,967,626 | 1,517,261 | 77.11% | 13.18 | 17.58% | 9.78% |
| SZ15 | 2,380,916 | 1,865,606 | 78.36% | 13.72 | 19.27% | 11.79% |
| SZ2 | 2,257,536 | 1,755,401 | 77.76% | 12.81 | 19.38% | 11.81% |
| SZ3 | 3,751,572 | 2,699,541 | 71.96% | 16.49 | 21.89% | 14.43% |
| SZ4 | 4,090,504 | 3,052,092 | 74.61% | 17.61 | 22.21% | 15.54% |
| SZ5 | 2,499,242 | 1,887,825 | 75.54% | 13.24 | 19.78% | 12.42% |
| SZ6 | 2,346,022 | 1,810,276 | 77.16% | 13.06 | 19.61% | 12.00% |
| SZ7 | 3,800,702 | 2,840,995 | 74.75% | 16.6 | 22.03% | 15.28% |
| SZ8 | 3,743,992 | 2,797,344 | 74.72% | 16.32 | 22.13% | 15.27% |
| SZ9 | 2,908,076 | 2,231,281 | 76.73% | 14.28 | 20.64% | 13.77% |
| TB10 | 5,984,114 | 4,270,657 | 71.37% | 22.1 | 23.10% | 17.49% |
| TB11 | 1,651,038 | 1,241,849 | 75.22% | 11.06 | 18.93% | 8.91% |
| TB14 | 1,212,356 | 886,425 | 73.12% | 10.52 | 17.08% | 6.05% |
| TB15 | 1,051,556 | 744,438 | 70.79% | 10.68 | 15.83% | 4.71% |
| TB16 | 1,532,118 | 1,133,966 | 74.01% | 11.27 | 18.22% | 7.78% |
| TB17 | 1,469,906 | 1,093,370 | 74.38% | 10.91 | 18.18% | 7.67% |
| TB18 | 1,457,976 | 1,103,267 | 75.67% | 11.03 | 18.09% | 7.72% |
| TB2 | 4,101,730 | 2,950,458 | 71.93% | 16.89 | 22.21% | 15.57% |
| TB3 | 4,165,892 | 2,931,419 | 70.37% | 16.61 | 22.50% | 15.62% |
| TB4 | 4,111,648 | 2,859,793 | 69.55% | 16.52 | 22.34% | 15.26% |
| TB5 | 4,346,656 | 3,095,261 | 71.21% | 17.3 | 22.52% | 15.95% |
| TB6 | 4,006,472 | 2,792,350 | 69.70% | 15.95 | 22.46% | 15.42% |
| TB7 | 3,817,386 | 2,691,444 | 70.50% | 16.03 | 22.22% | 14.71% |
| TB8 | 3,954,428 | 2,669,958 | 67.52% | 15.86 | 22.25% | 14.65% |
| TB9 | 3,947,044 | 2,689,490 | 68.14% | 16.07 | 22.35% | 14.59% |
| TMS1 | 3,520,748 | 2,724,892 | 77.40% | 15.93 | 21.80% | 15.30% |
| TMS10 | 3,069,080 | 2,256,830 | 73.53% | 13.91 | 21.64% | 14.24% |
| TMS11 | 3,050,812 | 2,238,188 | 73.36% | 13.52 | 21.90% | 14.48% |
| TMS12 | 2,747,988 | 1,952,209 | 71.04% | 12.7 | 21.57% | 13.11% |
| TMS13 | 3,085,856 | 2,291,552 | 74.26% | 13.5 | 21.99% | 14.89% |
| TMS14 | 3,385,402 | 2,497,642 | 73.78% | 14.51 | 21.97% | 15.33% |
| TMS15 | 4,295,562 | 3,202,265 | 74.55% | 17.22 | 22.96% | 16.74% |
| TMS2 | 3,640,846 | 2,793,988 | 76.74% | 15.56 | 22.01% | 16.15% |
| TMS3 | 2,592,618 | 1,967,965 | 75.91% | 13.05 | 20.83% | 13.09% |
| TMS4 | 3,288,374 | 2,493,277 | 75.82% | 14.99 | 21.41% | 14.76% |
| TMS5 | 3,206,074 | 2,414,351 | 75.31% | 14.47 | 21.40% | 14.77% |
| TMS6 | 1,944,146 | 1,377,232 | 70.84% | 11.74 | 18.67% | 9.57% |
| TMS7 | 2,745,758 | 1,992,729 | 72.57% | 13.07 | 21.22% | 13.13% |
| TMS8 | 2,566,596 | 1,841,833 | 71.76% | 12.23 | 21.08% | 12.82% |
| TMS9 | 1,928,752 | 1,398,932 | 72.53% | 11.18 | 19.40% | 10.24% |
| TZ1 | 3,585,348 | 2,554,662 | 71.25% | 15.74 | 21.37% | 14.28% |
| TZ10 | 4,127,870 | 2,938,944 | 71.20% | 16.55 | 22.35% | 15.81% |
| TZ11 | 3,896,372 | 2,749,309 | 70.56% | 15.82 | 22.67% | 15.27% |
| TZ12 | 3,962,498 | 2,843,513 | 71.76% | 16.01 | 22.23% | 15.81% |
| TZ13 | 2,764,536 | 1,912,613 | 69.18% | 13.93 | 19.77% | 11.71% |
| TZ14 | 2,833,620 | 1,944,346 | 68.62% | 13.92 | 20.06% | 11.93% |
| TZ15 | 4,017,482 | 2,873,416 | 71.52% | 16.97 | 22.17% | 14.98% |
| TZ16 | 4,122,988 | 2,835,025 | 68.76% | 16.29 | 22.42% | 15.31% |
| TZ2 | 3,665,454 | 2,643,194 | 72.11% | 16.02 | 21.46% | 14.59% |
| TZ4 | 3,489,338 | 2,462,231 | 70.56% | 15.2 | 21.66% | 14.15% |
| TZ5 | 4,621,120 | 3,266,202 | 70.68% | 17.83 | 22.94% | 16.31% |
| TZ6 | 4,056,500 | 2,847,971 | 70.21% | 16.16 | 22.37% | 15.59% |
| TZ7 | 3,946,898 | 2,815,033 | 71.32% | 16.13 | 22.16% | 15.51% |
| TZ8 | 3,418,168 | 2,284,919 | 66.85% | 14.83 | 22.13% | 13.06% |
| TZ9 | 3,053,820 | 2,106,925 | 68.99% | 13.7 | 21.98% | 13.01% |
| XE1 | 2,373,322 | 1,841,727 | 77.60% | 13.55 | 19.56% | 11.68% |
| XE10 | 2,950,936 | 2,258,420 | 76.53% | 14.39 | 20.47% | 13.88% |
| XE11 | 1,566,004 | 1,156,804 | 73.87% | 11.08 | 17.01% | 8.45% |
| XE12 | 2,508,444 | 1,914,339 | 76.32% | 13.36 | 19.81% | 12.41% |
| XE13 | 2,380,046 | 1,820,645 | 76.50% | 13.43 | 19.40% | 11.58% |
| XE14 | 2,142,520 | 1,681,985 | 78.50% | 13.01 | 18.85% | 10.99% |
| XE15 | 2,464,146 | 1,922,000 | 78.00% | 13.72 | 19.47% | 12.15% |
| XE2 | 2,530,870 | 1,930,879 | 76.29% | 13.87 | 19.51% | 12.18% |
| XE3 | 4,439,202 | 3,161,147 | 71.21% | 16.8 | 23.05% | 16.91% |
| XE4 | 2,453,372 | 1,845,678 | 75.23% | 13.04 | 19.73% | 12.26% |
| XE5 | 2,945,444 | 2,247,254 | 76.30% | 14.82 | 20.16% | 13.37% |
| XE6 | 3,279,382 | 2,369,207 | 72.25% | 14.9 | 21.42% | 13.94% |
| XE7 | 2,952,136 | 2,331,302 | 78.97% | 14.96 | 20.45% | 13.81% |
| XE8 | 2,698,800 | 2,087,625 | 77.35% | 13.85 | 20.31% | 13.24% |
| XE9 | 3,182,618 | 2,438,489 | 76.62% | 15.25 | 21.37% | 14.18% |

**TABLE S7.** Evaluation indices (AUC, Kappa, TSS) for the potential distribution areas of *C. kousa* under current and future (RCP45, RCP85, 2070) climate scenarios, based on the ensemble model.

|  |  | **MaxEnt** | **SRE** | **CTA** | **RF** | **MARS** | **FDA** | **GLM** | **GBM** | **GAM** | **ANN** | **Mean** |
| --- | --- | --- | --- | --- | --- | --- | --- | --- | --- | --- | --- | --- |
| **Current** | AUC | 0.886 | 0.790 | 0.788 | 0.900 | 0.891 | 0.88 | 0.901 | 0.907 | 0.893 | 0.893 | 0.873 |
|  | Kappa | 0.425 | 0.377 | 0.369 | 0.497 | 0.459 | 0.436 | 0.462 | 0.541 | 0.469 | 0.463 | 0.450 |
|  | TSS | 0.707 | 0.579 | 0.598 | 0.772 | 0.698 | 0.688 | 0.732 | 0.745 | 0.707 | 0.730 | 0.696 |
| **RCP45** | AUC | 0.656 | 0.592 | 0.634 | 0.754 | 0.737 | 0.773 | 0.712 | 0.727 | 0.652 | 0.746 | 0.698 |
|  | Kappa | 0.899 | 0.796 | 0.805 | 0.911 | 0.921 | 0.914 | 0.914 | 0.914 | 0.855 | 0.928 | 0.886 |
|  | TSS | 0.539 | 0.399 | 0.458 | 0.491 | 0.553 | 0.465 | 0.551 | 0.525 | 0.394 | 0.578 | 0.495 |
| **RCP85** | AUC | 0.723 | 0.656 | 0.615 | 0.760 | 0.697 | 0.763 | 0.732 | 0.754 | 0.708 | 0.689 | 0.710 |
|  | Kappa | 0.914 | 0.828 | 0.788 | 0.917 | 0.913 | 0.892 | 0.916 | 0.916 | 0.897 | 0.891 | 0.887 |
|  | TSS | 0.596 | 0.463 | 0.513 | 0.541 | 0.542 | 0.432 | 0.535 | 0.541 | 0.511 | 0.541 | 0.522 |

**TABLE S8.** The Deviance Information Criterion (DIC) value and the correlation associated with the simulation of POPs.

| Label | K max | DIC | Correlation value |
| --- | --- | --- | --- |
| RUN_000001 | 2 | 131,145 | 0.963995 |
| RUN_000002 | 2 | 133,699 | 0.982028 |
| RUN_000003 | 3 | 138,743 | 0.997337 |
| RUN_000004 | 3 | 139,346 | 0.997665 |
| RUN_000005 | 3 | 143,162 | 0.999683 |
| RUN_000006 | 4 | 141,061 | 0.998991 |
| RUN_000007 | 4 | 137,862 | 0.996914 |
| RUN_000008 | 4 | 144,258 | 0.999935 |
| RUN_000009 | 5 | 140,936 | 0.998929 |
| RUN_000010 | 5 | 137,135 | 0.996229 |
| RUN_000011 | 5 | 140,210 | 0.999332 |
| RUN_000012 | 6 | 135,146 | 0.995674 |
| RUN_000013 | 6 | 129,714 | 0.985047 |
| RUN_000014 | 6 | 130,994 | 0.923916 |
| RUN_000015 | 7 | 144,783 | 0.999985 |
| RUN_000016 | 7 | 131,807 | 0.989657 |
| RUN_000017 | 7 | 132,165 | 0.991216 |
| RUN_000018 | 8 | 131,343 | 0.996315 |
| RUN_000019 | 8 | 144,903 | 0.999985 |
| RUN_000020 | 8 | 140,144 | 0.998872 |
| RUN_000021 | 9 | 144,952 | 0.999969 |
| RUN_000022 | 9 | 126,395 | 0.979378 |
| RUN_000023 | 9 | 145,105 | 0.999984 |
| RUN_000024 | 10 | 131,588 | 0.991854 |
| RUN_000025 | 10 | 145,258 | 0.999975 |
| RUN_000026 | 10 | 145,125 | 0.999974 |
| RUN_000027 | 11 | 145,154 | 0.999984 |
| RUN_000028 | 11 | 135,489 | 0.936288 |
| RUN_000029 | 11 | 130,999 | 0.992020 |
| RUN_000030 | 12 | 138,503 | 0.998351 |
| RUN_000031 | 12 | 144,542 | 0.999916 |
| RUN_000032 | 12 | 144,634 | 0.999923 |
| RUN_000033 | 13 | 127,616 | 0.979146 |
| RUN_000034 | 13 | 129,104 | 0.953923 |
| RUN_000035 | 13 | 129,523 | 0.986803 |
| RUN_000036 | 14 | 143,185 | 0.999760 |
| RUN_000037 | 14 | 139,395 | 0.998778 |
| RUN_000038 | 14 | 127,593 | 0.973633 |
| RUN_000039 | 15 | 145,361 | 0.999977 |
| RUN_000040 | 15 | 129,048 | 0.970387 |
| RUN_000041 | 15 | 141,320 | 0.999432 |
| RUN_000042 | 16 | 145,371 | 0.999979 |
| RUN_000043 | 16 | 145,006 | 0.999929 |
| RUN_000044 | 16 | 145,335 | 0.999963 |
| RUN_000045 | 17 | 144,990 | 0.999943 |
| RUN_000046 | 17 | 145,414 | 0.999982 |
| RUN_000047 | 17 | 145,391 | 0.999973 |
| RUN_000048 | 18 | 145,240 | 0.999952 |
| RUN_000049 | 18 | 136,257 | 0.938144 |
| RUN_000050 | 18 | 135,014 | 0.996715 |
| RUN_000051 | 19 | 133,373 | 0.995118 |
| RUN_000052 | 19 | 145,356 | 0.999976 |
| RUN_000053 | 19 | 132,021 | 0.993785 |
| RUN_000054 | 20 | 145,398 | 0.999975 |
| RUN_000055 | 20 | 145,416 | 0.999982 |
| RUN_000056 | 20 | 145,407 | 0.999980 |
| RUN_000057 | 21 | 144,862 | 0.999935 |
| RUN_000058 | 21 | 136,299 | 0.919160 |
| RUN_000059 | 21 | 143,479 | 0.999853 |
| RUN_000060 | 22 | 136,083 | 0.926657 |
| RUN_000061 | 22 | 145,222 | 0.999963 |
| RUN_000062 | 22 | 135,163 | 0.952124 |
| RUN_000063 | 23 | 135,931 | 0.933178 |
| RUN_000064 | 23 | 142,082 | 0.999620 |
| RUN_000065 | 23 | 136,168 | 0.918384 |
| RUN_000066 | 24 | 145,416 | 0.999978 |
| RUN_000067 | 24 | 145,245 | 0.999964 |
| RUN_000068 | 24 | 134,707 | 0.996385 |
| RUN_000069 | 25 | 145,394 | 0.999971 |
| RUN_000070 | 25 | 145,307 | 0.999972 |
| RUN_000071 | 25 | 130,558 | 0.985004 |

**TABLE S9.** Statistical table of 72 outlier loci detected by BayeScan.

| Outlier loci |
| --- |
| SNP_134，SNP_153，SNP_270，SNP_271，SNP_272，SNP_273，SNP_355，SNP_389，SNP_390，SNP_391，SNP_393，SNP_1184，SNP_1185，SNP_1186，SNP_1379，SNP_1380，SNP_1382，SNP_1418，SNP_1584，SNP_1587，SNP_1588，SNP_1589，SNP_1651，SNP_1652，SNP_1653，SNP_1656，SNP_1657，SNP_1658，SNP_1662，SNP_1663，SNP_1789，SNP_1937，SNP_2239，SNP_2240，SNP_2241，SNP_2242，SNP_2708，SNP_3102，SNP_3105，SNP_3305，SNP_3311，SNP_3405，SNP_3406，SNP_3410，SNP_3435，SNP_3437，SNP_3439，SNP_3443，SNP_4120，SNP_4462，SNP_4465，SNP_4466，SNP_4506，SNP_4889，SNP_5402，SNP_5403，SNP_5407，SNP_5408，SNP_5508，SNP_5509，SNP_5530，SNP_5534，SNP_5535，SNP_5545，SNP_5546，SNP_5547，SNP_5553，SNP_5678，SNP_5683，SNP_5684，SNP_5693，SNP_5888 |
